# Dynamic magneto-mechanical force in lysosomes induces durable macrophage repolarization for antitumor immunity

**DOI:** 10.1038/s41422-025-01217-1

**Published:** 2026-02-03

**Authors:** Yingze Li, Mengge Zheng, Zhenyan Zhu, Yajuan Zhang, Peng Ning, Haotian Chen, Rui Gao, Chang Xu, Xueyan Wei, Yali Liu, Yingying Wang, Ruimei Zhou, Yuan Li, Zhenguang Li, Cheng Lv, Chen Liu, Junfang Xu, Zihan Guo, Zhixiang Hu, Lan Fang, Ke Wei, Mengying Feng, Changshi Zhou, Yunlang She, Weiyan Sun, Erzhen Chen, Gustavo R. Plaza, Bin He, Jason Miska, Weiwei Yang, Yichao Tang, Haipeng Liu, Chang Chen, Yu Cheng

**Affiliations:** 1 Department of Thoracic Surgery, Shanghai Pulmonary Hospital, Tongji University School of Medicine, Shanghai, China; 2 Translational Research Institute of Brain and Brain-Like Intelligence, Shanghai Fourth People’s Hospital, School of Medicine, Tongji University, Shanghai, China; 3 Frontiers Science Center for Intelligent Autonomous Systems, Tongji University, Shanghai, China; 4 Collaborative Innovation Center for Brain Science, Tongji University, Shanghai, China; 5 Central Laboratory, Shanghai Pulmonary Hospital, Tongji University School of Medicine, Shanghai, China; 6 School of Mechanical Engineering, Tongji University, Shanghai, China; 7 Shanghai Institute of Thoracic Oncology, Shanghai Chest Hospital, Shanghai Jiao Tong University School of Medicine, Shanghai, China; 8 Key Laboratory of Multi-cell Systems, Shanghai Key Laboratory of Molecular Andrology, CAS Center for Excellence in Molecular Cell Science, Shanghai Institute of Biochemistry and Cell Biology, University of Chinese Academy of Sciences, Chinese Academy of Sciences, Shanghai, China; 9 Tongji University Cancer Center, Shanghai Tenth People’s Hospital, School of Medicine, Tongji University, Shanghai, China; 10 Institute for Regenerative Medicine, Shanghai East Hospital, Shanghai Institute of Stem Cell Research and Clinical Translation, Shanghai Key Laboratory of Signaling and Disease Research, Frontier Science Center for Stem Cell Research, School of Life Sciences and Technology, Tongji University, Shanghai, China; 11 National Key Laboratory of Autonomous Intelligent Unmanned Systems, Tongji University, Shanghai, China; 12 College of Electronics and Information Engineering, Tongji University, Shanghai, China; 13 Center for Biomedical Technology, Universidad Politécnica de Madrid, Pozuelo de Alarcón, Madrid Spain; 14 Department of Neurological Surgery, Lou and Jean Malnati Brain Tumor Institute, Northwestern, University Feinberg School of Medicine, Chicago, IL USA; 15 Key Laboratory of Systems Health Science of Zhejiang Province, School of Life Science, Hangzhou Institute for Advanced Study, University of Chinese Academy of Sciences, Hangzhou, Zhejiang China

**Keywords:** Tumour immunology, Lysosomes

## Abstract

Mechanical forces are emerging physical cues that regulate biochemical signals of immune cells for antitumor immunity. Owing to the lack of precise tools to impose intracellular forces, little is known about whether and how organelle-level forces trigger mechanotransduction for antitumor immunity. Here, we developed a magneto-mechanical force-triggered lysosomal membrane permeabilization (MagLMP) strategy to induce durable macrophage repolarization for in vivo applications. Self-assembled magnetic nanomotors are driven by rotational magnetic fields, facilitating dynamic damage to the lysosomal membrane by a finely tuned torque-induced vortex. Intriguingly, galectin 9 (Gal9) was found to be critical for sensing cyclic MagLMP, which dynamically activated AMP-activated protein kinase (AMPK), enhanced activation of nuclear factor kappa B (NF-κB), and induced metabolic alterations for sustained M1-like macrophage repolarization, followed by mounting of antitumor immunity. Through single-cell RNA sequencing of tumor tissues, as well as macrophage depletion-reconstitution models involving intratumoral transfer of *Gal9*-KO bone marrow-derived macrophages (BMDMs) and AMPK shRNA-transduced *Gal9*-KO BMDMs, we confirmed the Gal9-AMPK-NF-κB axis as the essential pathway by which MagLMP functions in antitumor therapy. In a mouse model of lung adenocarcinoma in situ, overall survival was extended after intravenous administration of nanomotors followed by cyclic MagLMP, and one third of mice survived for more than 300 days. Together, these results demonstrate an intracellular mechanical strategy that can dynamically manipulate innate immune responses in vivo, providing a tool for durable immunotherapy through organelle mechanotransduction.

## Introduction

Over the past decade, significant efforts have been devoted to regulating biochemical signaling to enhance antitumor immunity,^1^ including exploration of new immune checkpoints, targeted delivery of stimulatory cytokines, and development of chimeric antigen receptor immune cells.^2–5^ Biochemically based immunotherapy strategies have shown curative effects in the treatment of patients with refractory tumors such as hematological malignancies.^6^ However, it is inherently challenging in terms of efficacy and safety to extend the clinical benefits of such therapies to more patients with solid tumors.^7,8^ For instance, only ~20% of advanced non-small cell lung cancer patients showed responses to anti-PD-1/PD-L1 therapy.^9,10^ Key challenges, including immune-suppressive microenvironments and heterogeneity of tumor cells, hinder antitumor outcomes aimed at curing cancer. Thus, it is necessary to explore novel immunotherapy avenues for precisely regulating biochemical signals to overcome these barriers to cancer immunotherapy.

Mechanotransduction is a biophysical regulatory process in which cells convert mechanical forces into biochemical signals,^11^ providing a new dimension to tuning immunity. Accumulating evidence has shown that mechanical stimuli influence the behaviors of immune cells, including their activation, cytokine release, and interaction with tumor cells.^12,13^ Attention has long been focused on the extracellular manipulation of mechanical cues to regulate mechanosensitive channels and cell adhesion molecules on the plasma membranes of immune cells. Physical approaches such as optical tweezers or atomic force microscopy (AFM) can generate local forces on the cell surface and mechanically modulate immune cell behaviors at the cellular level in vitro.^14,15^ Tissue-level forces derived from the extracellular matrix and fluid pressure can also serve as important modulators for regulating signaling pathways and metabolism to tune either innate or adaptive immune cells.^16–19^ Although it has become increasingly clear that mechanosensors on the plasma membrane of immune cells play a key role in responding to extracellular forces, organelles may sense these forces via membrane deformation, alteration of intraluminal fluid flow, cytoskeletal changes, or even intracellular mechanosensitive channels.^20^ However, owing to a lack of high-precision mechanical regulation tools for in vivo studies, little is known about whether and how organelles sense and respond to intracellular forces and trigger mechanotransduction for antitumor immunity.

Targeted manipulation of organelles is emerging as an important research focus in cancer therapy. In recent years, the lysosome, previously treated mainly as an organelle for the digestion of biological macromolecules, is now recognized as a dynamic signaling center that responds to multiple forms of stress associated with various physiological and pathological processes.^21,22^ Notably, changes in lysosomal membrane permeabilization (LMP) caused by small molecules such as Leu-Leu-O-Me (LLoMe), chloroquine (CQ), and salinomycin trigger the activation of lysosomal membrane-related biochemical signals for cell death.^23–25^ Galectins such as galectin 3 (Gal3) or Gal8 are considered classical indicators of LMP.^26–28^ Recruitment of Gal3 activates the endosomal sorting complex required for transport (ESCRT) and induces lysosomal membrane repair.^29^ LMP caused by LLoMe has also been shown to recruit Gal9 and induce cell autophagy through AMP-activated protein kinase (AMPK) activation associated with enhanced ubiquitination responses.^30^ However, current molecular approaches suffer from rapid metabolism, uncontrolled toxicity, and low selectivity, hampering the targeting of organelle-dependent activation of immune cells. In contrast to the well-documented process of lysosome-dependent cell death via LMP, the role of lysosomes in biochemical signaling for antitumor immunity has been overlooked.

Owing to spatiotemporal programmability and agile manipulability, physical strategies coupled with nanomaterials associated with universal endocytosis capability provide new opportunities for the targeted regulation of lysosomes.^31,32^ Among various mechanical approaches, magneto-mechanical nanomotors show superior magnetic drive capabilities, generating pN forces under alternating magnetic fields, and have produced antitumor effects through irreversible LMP in tumor cells with deep tissue penetration.^33^ This approach has also been used to activate surface receptors such as the Piezo1 ion channel and trigger signal transduction to control neuron functions.^34^ These studies have demonstrated that magneto-mechanical tools can be tailored to control cell behaviors via force generation. However, whether intracellular magneto-mechanical forces can trigger immune responses in vivo and the threshold range of such regulatory forces remain to be clarified. How to activate immune cells by mechanical regulation of organellar signal transduction without triggering cell death remains a dilemma.

To address this challenge, we propose a dynamic magneto-mechanical force-triggered lysosomal membrane permeabilization (MagLMP) strategy based on the fluid-structure coupling of assembled magnetic nanomotors (MNMs) in lysosomes, which can reversibly regulate LMP in macrophages in a programmable fashion and trigger cyclic signal transduction for the mounting of sustainable M1-like macrophage repolarization and durable antitumor immunity. Single-cell RNA sequencing (scRNA-seq) of tumor tissues, together with macrophage depletion-reconstitution models involving intratumoral transfer of *Gal9*-KO bone marrow-derived macrophages (BMDMs) and AMPK shRNA-transduced *Gal9*-KO BMDMs, identified the Gal9-AMPK-NF-κB axis as the key molecular mechanism by which MagLMP functions in antitumor therapy. To further evaluate the translational potential of MagLMP, we established an intravenous administration model in which MNMs were systemically injected and subsequently enriched at the tumor site using a static magnetic field. Under these conditions, the antitumor efficacy of MagLMP treatment was comparable to that observed with intratumoral delivery. A mouse model of lung adenocarcinoma in situ was developed, and MNMs were intravenously delivered and guided into the lung tissue by magnetic attraction, providing an additional demonstration of the feasibility of this strategy for the treatment of orthotopic tumors. After cyclic MagLMP treatment, one-third of the mice demonstrated a long-term overall survival benefit. This strategy extends the mechanical regulation of antitumor immunity from the extracellular level to the organelle level, providing a unique mechanical tool for targeted activation of immune responses in vivo.

## Results

### Assembled MNMs regulate reversible lysosomal membrane permeabilization by MagLMP

**Fig. 1:**
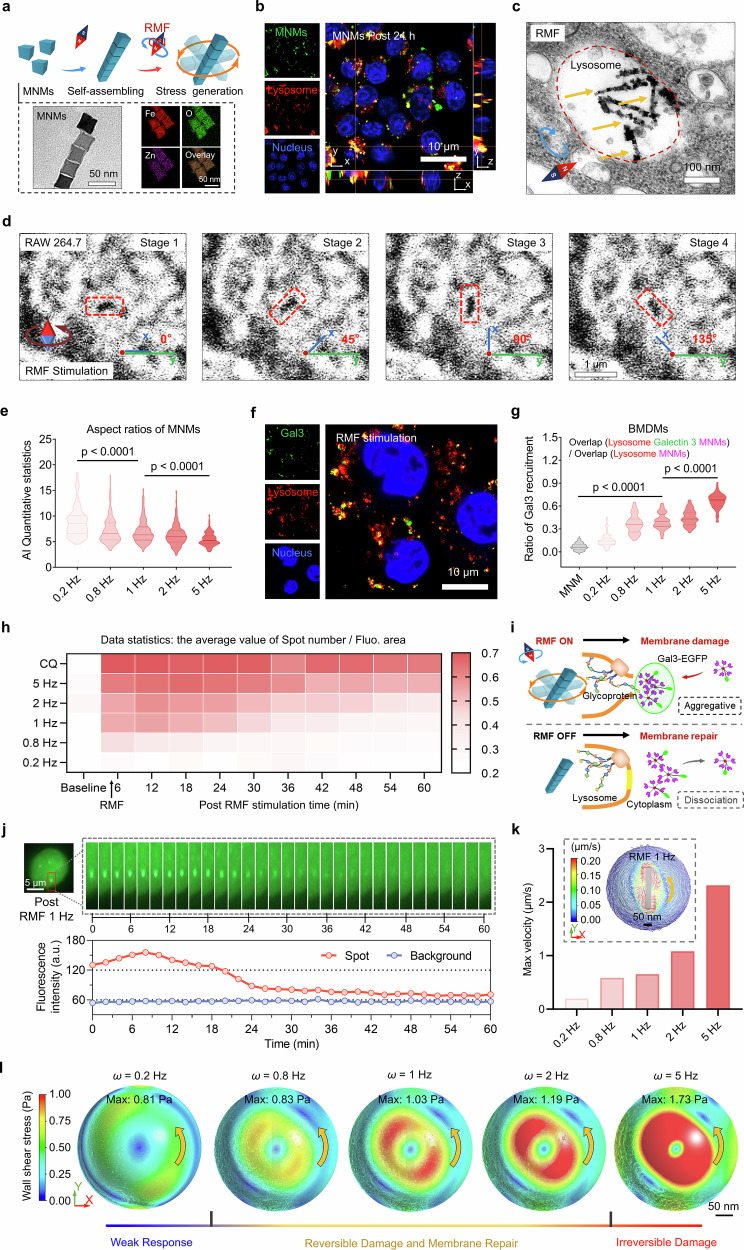
Assembled MNMs regulate reversible lysosomal membrane damage by MagLMP. **a** Schematic diagram of MNM self-assembly and stress generation by RMF stimulation. TEM images of MNM morphology and element mapping images of MNMs (purple, red, and green represent Zn, Fe and O, respectively) are shown. **b** 3D fluorescence confocal images of RAW 264.7 cells incubated with FITC-labeled MNMs (green) for 24 h. Lysosomes were stained with LysoTracker Red (red), and nuclei were stained with Hoechst (blue). **c** Bio-TEM images of RAW 264.7 cells incubated with MNMs for 24 h and then treated with 1 Hz RMF for 15 min. **d** RAW 264.7 cells were incubated with MNMs for 24 h and then treated with 1 Hz RMF. Representative continuous images of assembled MNMs under different rotational states in cells are shown. See Supplementary information, Fig. S1f and Videos S2–S4 for details. **e** Numbers of assembled MNMs at different frequencies as analyzed by a deep learning system using Bio-TEM images. Data are presented as means ± SD. Statistical significance is defined as *P* < 0.05 (360 Bio-TEM images in the training set, 40 images in the validation set, and 40 images in the testing set). **f**, **g** EGFP-Gal3-transfected BMDMs were incubated with MNMs for 24 h and then treated with 1 Hz RMF for 15 min. Lysosomes were stained with LysoTracker Red (red), and nuclei were stained with Hoechst (blue). Representative fluorescence images of these cells are shown (**f**). The recruitment ratio of Gal3 to lysosomes was calculated (**g**). Data are presented as means ± SD. Statistical significance was defined as *P* < 0.05 (*n* = 60 cells from 10 independent biological replicates). **h** EGFP-Gal3-transfected RAW 264.7 cells were incubated with MNMs for 24 h and then treated with the indicated RMF for 15 min or with CQ for 1 h. Continuous data for positive spot number (EGFP-Gal3) per fluorescent region are shown. All fluorescence-positive cells were counted at each time point in 9 wells of 96-well plates. **i** Schematic diagram of MagLMP-induced Gal3 aggregation with RMF on and subsequent Gal3 dissociation after RMF off. **j** EGFP-Gal3-transfected RAW 264.7 cells were incubated with MNMs for 24 h and then treated with 1 Hz RMF for 15 min. Representative continuous fluorescence images and fluorescence intensity of these cells are shown. **k**, **l** FEM simulation of the maximum velocity (**k**) and maximum wall shear stress (**l**) in a lysosome (with a measured radial dimension of 359.2 nm and a viscosity of 471.1 cp) under various RMF frequencies.

The vortex effect is a swirling motion of fluid or gas that creates a dynamic force in its surroundings. We hypothesized that when magnetically responsive nanomotors were submerged in a fluidic environment like the lysosome, they would induce a controllable fluid vortex within this organelle that could be actuated by a programmable magnetic field. The resulting mechanical signal might regulate lysosomal permeability in a reversible manner and initiate mechanotransduction in immune cells. We therefore designed a MagLMP strategy for immune cell activation. A cubical MNM (25.0 ± 2.6 nm) was designed to passively target lysosomes; it consisted of a magnetic nanoparticle (MNP) coated on the surface with polylysine (PLL) and had the ability to self-assemble into elongated rod-like microstructures (Fig. 1a; Supplementary information, Fig. S1a). The MNMs exhibited a high saturation magnetization (69 emu/g), demonstrating superparamagnetic-like properties for magneto-mechanical actuation (Supplementary information, Fig. S1b). Dynamic light scattering (DLS) analysis showed that the MNMs were highly dispersed in solution (Supplementary information, Fig. S1c). The zeta potential of the MNMs was 30.8 ± 0.9 mV, enabling them to bind to negatively charged cell membranes (Supplementary information, Fig. S1c). Due to their phagocytosis ability and their role as key players in the innate immune system, we selected macrophages as the immune cells for lysosome-targeted regulation. 3D confocal microscopy revealed significantly enhanced co-localization of MNMs and lysosomes in macrophages at 24 h after incubation compared with the 2-h time point (Fig. 1b; Supplementary information, Fig. S1d). Moreover, 79.6% ± 4.2% of the FITC-labeled MNMs (green) were located within lysosomes (red) after 24 h of incubation with macrophages (Supplementary information, Fig. S1e).

Given the magnetic responsiveness of the MNMs, we used a rotating magnetic field (RMF) to control their dynamic mechanical output. When exposed to RMF, the MNMs self-assembled into rod-like shapes in the lysosomes (Fig. 1c; Supplementary information, Fig. S1f), which facilitated the generation of magneto-mechanical force within these organelles. The self-assembly and rotational motion of MNMs were also visualized in vitro under RMF stimulation, and the local fluidic vortices induced by rotation of the MNM assemblies were successfully characterized (Supplementary information, Fig. S1g and Video S1). Interestingly, we could directly observe the rotational motion of the assembled nanomotors in the lysosomes (Fig. 1d; Supplementary information, Fig. S1h and Videos S2–S4). Further investigation revealed that magnetic field strength was a key parameter governing the motion efficiency of the MNM assemblies, and a significant increase in rotational activity was observed when the field strength increased from 4 mT to 20 mT (Supplementary information, Fig. S1i, j and Videos S5–S8). We used biological transmission electron microscopy (Bio-TEM) images and deep learning analysis by artificial intelligence (AI) to capture the assembled aspect ratio of MNMs located in lysosomes at different RMF frequencies. The results suggested that the self-assembly behavior of MNMs was frequency dependent, consistent with the results of mathematical models (Fig. 1e; Supplementary information, Fig. S1k).

We next explored how the mechanical force generated by assembled MNMs affected the lysosomal membrane. Previous studies have shown that Gal3 can be rapidly recruited to damaged sites on the lysosomal membrane and then diffuse into the cytoplasm when the membrane damage is repaired.^26,27^ We therefore fused Gal3 with enhanced green fluorescent protein (EGFP-Gal3) to monitor LMP. RMF stimulation resulted in the formation of Gal3 foci, which were absent in cells incubated with MNMs alone without RMF stimulation. These foci appeared to co-localize with lysosomes, indicating that MagLMP executed by the assembled MNMs effectively caused lysosomal membrane damage (Fig. 1f; Supplementary information, Fig. S2a). We calculated the recruitment efficiency of Gal3 in RAW 264.7 cells and in BMDMs isolated from C57BL/6 mice at different frequencies of RMF. The results indicated that recruitment of Gal3 was positively correlated with the frequency of stimulation (Fig. 1g; Supplementary information, Fig. S2b). Excitingly, high-content imaging analysis showed that the permeabilization and repair of lysosomes were highly dependent on the frequency of RMF (Fig. 1h; Supplementary information, Videos S9–S14). Efficient MagLMP was achieved with an RMF frequency of 1 Hz, and the membrane was repaired rapidly within 30 min post-treatment (Fig. 1i, j; Supplementary information, Fig. S2c, d). Increasing the RMF frequency beyond 1 Hz delayed lysosomal membrane repair. When RMF frequency increased to 5 Hz, swift and permanent membrane rupture was observed, similar to the irreversible damage caused by CQ (Fig. 1h). On the other hand, frequencies below 1 Hz were less efficient in producing LMP. These results demonstrated that the optimal stimulation frequency for reversible LMP was ~1 Hz, as this frequency produced the desired permeabilization effect without causing permanent damage to the lysosomes. The results also showed no damage to cell membranes or membranes of other organelles, including the mitochondria, nuclei, and endoplasmic reticulum, after RMF stimulation (Supplementary information, Fig. S2e–j), indicating that MagLMP could be precisely regulated by selective lysosomal targeting of the MNMs.

To clarify the effect of RMF stimulation on lysosome damage, we investigated the association between the rotating assembled nanomotors and the lysosomal membrane. Given the high fluid viscosity within the lysosomes, the rotational motion of the assembled nanomotors was expected to generate fluid flow and thus apply anisotropic hydrodynamic shear stress to the lysosomal membrane. The statistical results of Bio-TEM revealed that most MNM assemblies under different frequencies of RMF were not in contact with the lysosomal membranes, suggesting that hydrodynamic shear stress was the primary contributor to MagLMP (Supplementary information, Fig. S3a, b).

To quantitatively determine the reversible permeabilization effect of lysosomes caused by the rotating nanomotors, we analyzed fluid dynamics within the lysosomes and the shear stress distribution on the lysosomal membrane through finite element method (FEM) simulation using measured lysosomal dimensions and the viscosity of the enclosed fluid. We labeled lysosomes with FITC-Dextran to determine the average radial dimensions of lysosomes in macrophages, which averaged 379 nm in macrophages and 439 nm after culture with MNMs (Supplementary information, Fig. S3c). DCVJ, a viscosity-sensitive fluorescent probe, exhibited an ~12-fold increase in fluorescence intensity as the viscosity of the solution rose from 40 cP to 402 cP (Supplementary information, Fig. S3d), confirming its suitability for assessment of lysosomal fluid viscosity. After staining macrophages with DCVJ, lysosomal fluorescence intensity in the MNM and RMF groups was higher than that in the PBS group, indicating that lysosomal fluid viscosity increased following MNM co-incubation (Supplementary information, Fig. S3e, f). When the lysosomal fluid was extracted from macrophages and analyzed using DCVJ, it showed an average viscosity of 90.1 cP in untreated cells and 471.1 cP after 24 h of MNM co-incubation; these values could be used for more accurate FEM simulations (Supplementary information, Fig. S3g). We next analyzed the spatial coordinates of the assembled MNMs within lysosomes through FEM simulations. Given that the magnetic system we used only produced magnetic torques, the spatial displacements of the MNMs were determined only by the hydraulic force exerted by the lysosomal fluid. When the center of the MNMs coincided with the center of the lysosome, the pressure around the MNMs became radially symmetrical (Supplementary information, Fig. S3h). Consequently, the cyclic hydraulic force acting on the MNMs was balanced, enabling them to maintain rotation in that particular position. However, if the MNMs were disturbed and shifted away from the lysosomal center, the pressure increased on the side of the MNMs closer to the lysosomal membrane (Supplementary information, Fig. S3i, j), prompting them to return to the energy-favorable state in which their center aligned with the lysosomal center. We then simulated the effect of magneto-mechanical regulation on lysosomes with different radial dimensions (Supplementary information, Fig. S3k). The simulation results showed that under a 260-mT magnetic field, both the maximum fluid velocity and the maximum wall shear stress increased monotonically with RMF frequency (Fig. 1k, l). This trend also held for lysosomes with larger radial dimensions (Supplementary information, Fig. S4a, b). In addition, as the actuation frequency increased from 0.2 Hz to 5 Hz, we observed a > 2-fold increase in the maximum wall shear stress on the membrane, indicating that the adjustment of magnetic signals could regulate the shear stress applied to the targeted lysosomal membrane induced by the assembled MNMs. For example, the maximum wall shear stress reached 1.03 Pa when the nanomotors rotated at 1 Hz, consistent with previous reports.^35–37^ These studies showed that shear stress at ~1 Pa could cause significant changes in membrane permeability and induce lysosomal membrane damage. Notably, during rotation of the MNMs, particular membrane regions (highlighted by the red contour in Fig. 1l) experienced higher shear stress due to the higher fluidic shear rate in these areas (Fig. 1k), providing insight into which regions of the membrane are most susceptible to MagLMP.

To further confirm the regulation of LMP by magneto-mechanical force, we constructed an in vitro model of a lysosomal-like small lipid vesicle (SUV) encapsulating a high concentration of Rhodamine B, with or without MNMs (Supplementary information, Fig. S4c–e). Owing to fluorescence quenching at high intravesicular concentrations, rhodamine exhibited a low signal within intact vesicles. Upon membrane disruption, dilution of rhodamine into the surrounding solution led to a marked increase in fluorescence intensity.^38^ As the frequency of RMF stimulation increased, the intensity of fluorescence also increased owing to the release of Rhodamine B, confirming frequency-dependent membrane permeabilization by MagLMP (Supplementary information, Fig. S4f). Combining the results of high-content imaging analysis and FEM simulation, we concluded that a range of 0.8 to 2 Hz under RMF stimulation could mildly regulate lysosomal membrane damage and then allow the repair of lysosomal membranes in macrophages.

### MagLMP induces efficient and persistent macrophage repolarization

**Fig. 2:**
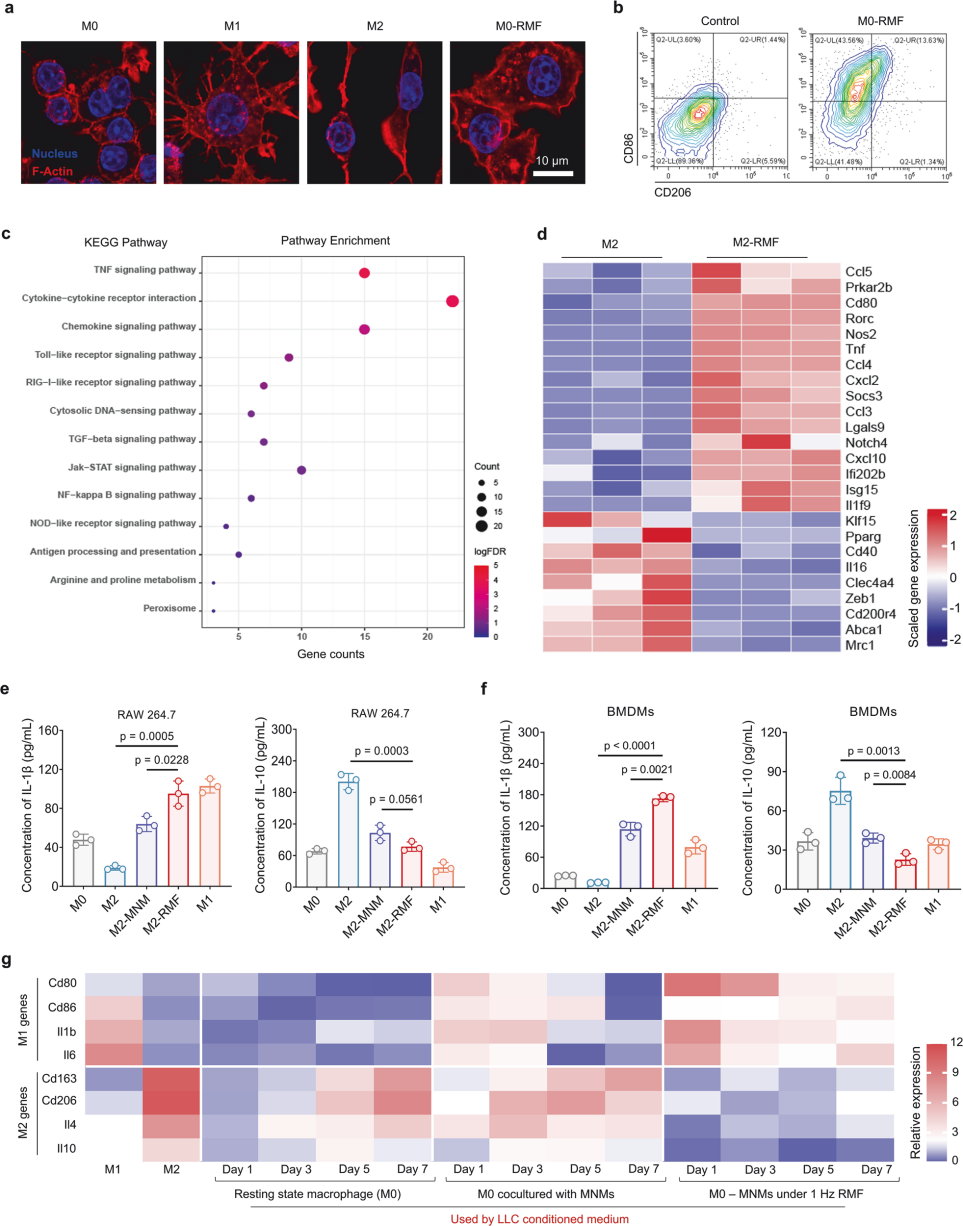
MagLMP induces efficient and persistent macrophage repolarization. **a** RAW 264.7 cells were polarized into M0, M1 (induced by IFN-γ and LPS), or M2 (induced by IL-4 and IL-13) macrophages. M0 macrophages were incubated with MNMs and then treated with 1 Hz RMF for 15 min. Macrophages were stained with ActinRed (red), and nuclei were stained with Hoechst (blue). **b** Polarized M0 RAW 264.7 cells were incubated with MNMs and then treated with 1 Hz RMF for 15 min. Flow cytometry analysis of CD86 and CD206 expression was performed. **c**, **d** RNA-sequencing analyses of polarized M2 RAW 264.7 cells treated with or without 1 Hz RMF for 15 min. KEGG enrichment analysis and heat map analysis were performed (*n* = 3 independent biological replicates). **e**, **f** RAW 264.7 cells or BMDMs were polarized into M0, M1, or M2 macrophages. M2 macrophages were incubated with MNMs and then treated with or without 1 Hz RMF for 15 min (M2-MNM represents M2 macrophages incubated with MNMs, and M2-RMF represents M2 macrophages incubated with MNMs and treated with RMF). Concentrations of M1-associated proteins (IL-1β) and M2-associated proteins (IL-10) were examined in the conditioned medium of these cells. Data are presented as means ± SD. Statistical significance was defined as *P* < 0.05 (*n* = 3 independent biological replicates). **g** RAW 264.7 cells were polarized into M0, M1, or M2 macrophages. M0 macrophages were cultured in conditioned medium derived from LLC and incubated with or without MNMs, then treated with or without 1 Hz RMF for 15 min. mRNA levels of M1-associated genes (*Cd80*, *Cd86*, *Il1b*, *Il6*) and M2-associated genes (*Cd163*, *Cd206*, *Il4*, *Il10*) were examined. Data are presented as means ± SD (*n* = 4 independent biological replicates).

Having demonstrated that an RMF of 1 Hz for 15 min induced recoverable lysosomal membrane damage, we further explored its regulatory effect on macrophages. We first investigated potential cytotoxicity, as minimizing cytotoxicity is a prerequisite for MagLMP activation of macrophages. Prussian blue staining and quantification of iron levels demonstrated that macrophages had a strong ability to take up MNMs (Supplementary information, Fig. S5a, b). In addition, programmable MagLMP with a lower frequency showed limited cytotoxicity. The vast majority of cells survived after MNM incubation and MagLMP treatment (Supplementary information, Fig. S5c, d). Taking advantage of the programmability of RMF, we subjected macrophages (both RAW 264.7 and BMDMs) to RMF stimulation for 7 days, during which time no long-term cytotoxicity was observed (Supplementary information, Fig. S5e, f). A fluorescence assay of the macrophage cytoskeleton showed that RMF stimulation induced pseudopodia formation,^39^ indicating that MagLMP induced M1 polarization of macrophages (Fig. 2a). We used flow cytometry to analyze the expression of the M1 marker CD86 and the M2 marker CD206 in macrophages and found that 1 Hz RMF stimulation of macrophages caused significant upregulation of CD86 rather than CD206 (Fig. 2b).

To test the repolarization potential from M2-like to M1-like subtypes by MagLMP, we treated macrophages with IL-4 and IL-13 to induce the M2-like subtype, then applied RMF stimulation. Kyoto Encyclopedia of Genes and Genomes (KEGG) pathway analysis of RNA sequencing data showed that genes upregulated in the M2-RMF group were enriched in immune responses and inflammatory-associated signaling pathways, including the tumor necrosis factor (TNF), chemokine signaling, Toll-like receptor, and cytosolic DNA-sensing signaling pathways (Fig. 2c). Gene expression analysis also demonstrated that 1 Hz of RMF treatment (M2-RMF) robustly upregulated M1-related genes, such as *Tnf* and *Cd80*, as well as chemokines of the CXC and CC families (e.g., *Cxcl10* and *Ccl4*), in M2 macrophages (Fig. 2d). We next measured the expression of specific genes of the M1 and M2 subtypes in BMDMs and RAW 264.7 cells by real-time quantitative PCR (RT-qPCR) and confirmed that the MagLMP strategy induced repolarization of the M2-like subtype to the M1-like subtype (Supplementary information, Fig. S6a, b). Consistent with these results, MagLMP promoted secretion of the inflammatory factors IL-1β and IL-6 in the M2 subtype of BMDMs and RAW 264.7 cells and reduced levels of anti-inflammatory cytokines such as IL-4 and IL-10 (Fig. 2e, f; Supplementary information, Fig. S6c, d).

In general, the repolarization of macrophages towards the M1-like phenotype is inhibited by the tumor microenvironment, which tends to favor tumor-associated macrophages of the M2-like subtype that promote tumor development.^40^ To evaluate the efficiency of MagLMP-induced macrophage repolarization in the tumor microenvironment, we used Lewis lung carcinoma (LLC)-conditioned medium to culture macrophages throughout the process. The control groups gradually changed to the M2-like subtype in the presence of tumor cell culture medium (Fig. 2g). By contrast, the RMF-treated macrophages maintained an M1-like subtype over 7 days, indicating that MagLMP could repolarize macrophages from M2-like to M1-like and thereby reshape the tumor immune microenvironment.

### MagLMP triggers lysosomal membrane damage to activate the Gal9-AMPK-NF-κB axis

**Fig. 3:**
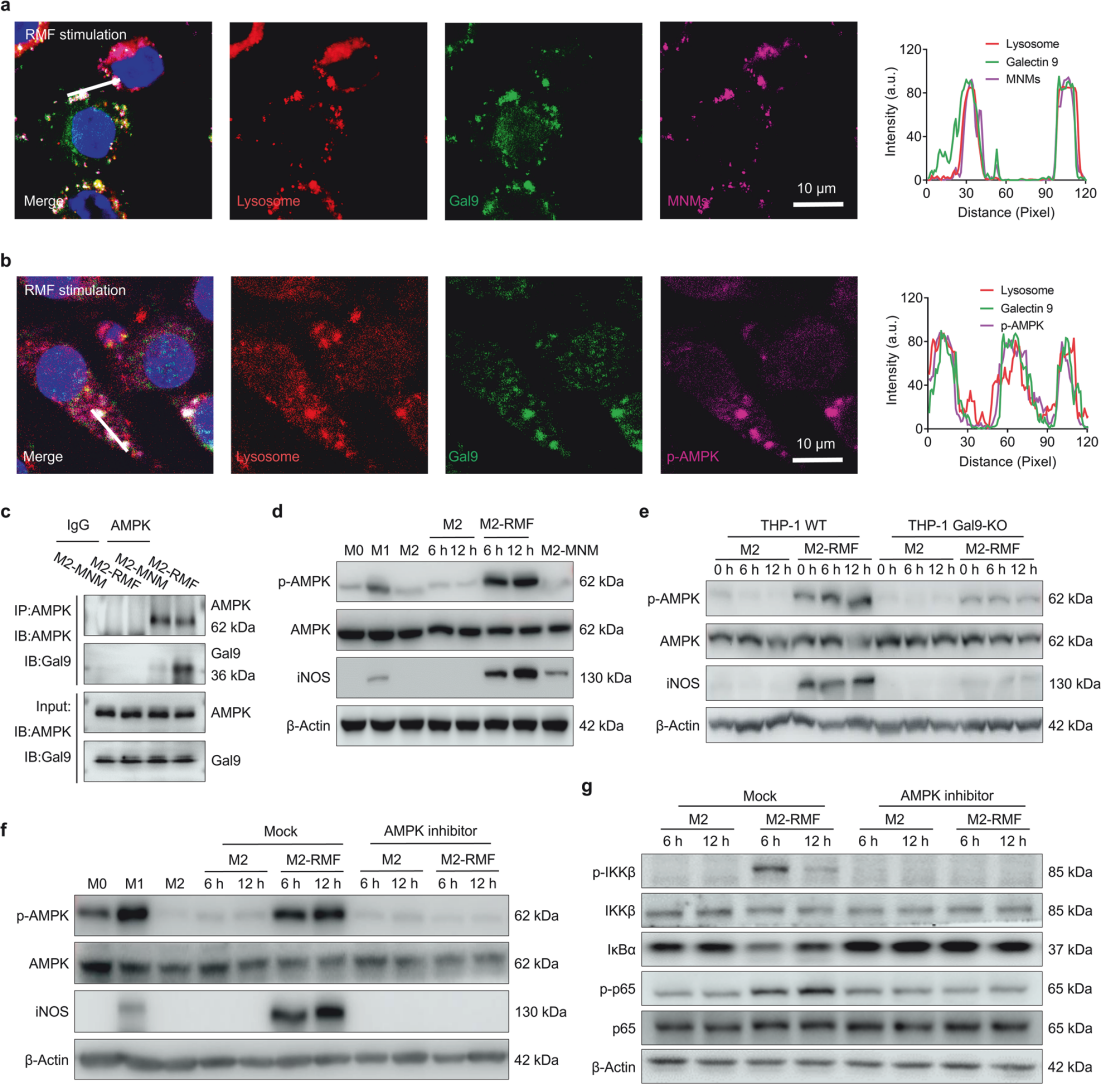
MagLMP triggers lysosomal membrane damage to activate the Gal9-AMPK-NF-κB axis. **a** YFP-Gal9-transfected RAW 264.7 cells were polarized into M2 macrophages. Cells were incubated with MNMs and then treated with 1 Hz RMF for 15 min. Lysosomes were stained with LysoTracker Red (red), nuclei were stained with Hoechst (blue), and MNMs were modified with Cy5 (purple). **b** YFP-Gal9-transfected RAW 264.7 cells were polarized into M2 macrophages. Cells were incubated with MNMs and treated with 1 Hz RMF for 15 min. Lysosomes were stained with LysoTracker Red (red), nuclei were stained with Hoechst (blue), and p-AMPK was stained with an anti-p-AMPK antibody (purple). **c** Co-IP analysis of AMPK and Gal9 was performed on RAW 264.7-derived M2 macrophages treated with MNMs or 1 Hz RMF for 15 min. IgG was used as a negative control. **d** RAW 264.7 cells were polarized into M0, M1, or M2 macrophages. M2 macrophages were incubated with or without MNMs and treated with or without 1 Hz RMF for 15 min, then analyzed by immunoblotting. **e** THP-1 cells with or without Gal9 knockout were polarized into M2 cells and treated with or without RMF for 6 h or 12 h. Levels of AMPK, p-AMPK, and iNOS were analyzed by western blotting. **f** RAW 264.7 cells were polarized into M0, M1, or M2 cells. Cells were treated with or without an AMPK inhibitor (10 μM) and with or without RMF for 6 h or 12 h. Levels of AMPK, p-AMPK, and iNOS were analyzed by western blotting. **g** RAW 264.7-derived polarized M2 cells were treated with or without an AMPK inhibitor (10 μM) and with or without RMF for 6 h or 12 h. Levels of IKKβ, p-IKKβ, IκBα, p65, and p-p65 were analyzed by western blotting.

We next investigated the mechanism underlying lysosome-targeted mechanotransduction in macrophages. Gal9 is a lysosome-related signaling molecule that has been reported to participate in the regulation of cell functions. Previous work reported that Gal9 responded to lysosomal membrane damage in response to bacterial infection and then induced autophagy by activating AMPK.^30^ We therefore constructed a yellow fluorescent protein (YFP)-fused Gal9 (YFP-Gal9) plasmid to trace the cellular distribution of Gal9. In immunofluorescence assays, Gal9 appeared to colocalize with lysosomes and MNMs within the lysosomes, as well as with phosphorylated AMPK (Fig. 3a, b), suggesting that MagLMP might activate the Gal9-AMPK signaling axis. Intriguingly, the recruitment efficiency of Gal9 was positively correlated with the frequency of RMF stimulation (Supplementary information, Fig. S7a, b), increasing from 12.4% to 57.6% in RAW 264.7 cells and from 10.9% to 58.1% in BMDMs as the RMF frequency increased from 0.2 Hz to 5 Hz, demonstrating that MagLMP could induce the recruitment of Gal9. Co-immunoprecipitation (Co-IP) assays revealed that MagLMP significantly enhanced the interaction between Gal9 and AMPK (Fig. 3c). Consistent with this result, MagLMP induced the phosphorylation of AMPK in M2-like macrophages, accompanied by increased expression of iNOS (a classically expressed protein of M1 macrophages) (Fig. 3d).

To further verify the contribution of the MagLMP-activated Gal9-AMPK axis to the induction of macrophage repolarization, we constructed a *Gal9* knockout human acute monocytic leukemia (THP-1) cell line (THP-1 *Gal9*-KO) (Supplementary information, Fig. S7c). The deletion of Gal9 inhibited MagLMP-induced phosphorylation of AMPK and expression of iNOS, indicating that Gal9 was critical for sensing mechanical-force-induced LMP and subsequent signal transduction in lysosomes (Fig. 3e). Metformin, an AMPK agonist, induced AMPK phosphorylation and iNOS expression (Supplementary information, Fig. S7d), whereas an AMPK inhibitor (Compound C) significantly inhibited iNOS expression (Fig. 3f), demonstrating that activation of AMPK via MagLMP was critical for M1-like macrophage repolarization. Consistent with these results, RT-qPCR measurement of M1/M2-related genes demonstrated that inhibition of AMPK by Compound C reduced the effects of macrophage repolarization by MagLMP (Supplementary information, Fig. S7e).

Previous work reported that phosphorylation of AMPK activates NF-κB,^41,42^ an essential process in macrophage repolarization toward the M1-like subtype. In the present study, levels of IKKβ phosphorylation (p-IKKβ) increased significantly upon MagLMP treatment and were accompanied by degradation of IκBα. Moreover, RMF stimulation also led to a significant increase in the phosphorylation level of p65, indicating activation of NF-κB after MagLMP treatment (Fig. 3g). Treatment with Compound C significantly inhibited MagLMP-induced IKKβ phosphorylation, IκBα degradation, and p65 phosphorylation, indicating that activation of NF-κB depended on activation of AMPK (Fig. 3g). Given the crucial role of AMPK in cell metabolism, we also examined metabolic changes in macrophages after MagLMP. A Seahorse assay revealed an increased level of glycolysis in macrophages, accompanied by a decreased level of oxidative phosphorylation (Supplementary information, Fig. S7f, g). We also performed metabolomics analysis of M2-like macrophages with or without MagLMP treatment. Pathway analysis showed that some metabolic pathways were significantly altered in M2-like macrophages after MagLMP stimulation compared with the M2 and M2-MNM groups, including glycolysis, the pentose phosphate pathway, and the citric acid cycle (Supplementary information, Fig. S7h, i), which have been shown to facilitate macrophage polarization toward the M1-like subtype.^43^ Heat map analysis showed that MagLMP treatment increased levels of metabolites related to glucose metabolism, such as pyruvate, citrate, and succinate, which have been reported to promote M1-like macrophage polarization (Supplementary information, Fig. S7j). Thus, the Gal9-AMPK axis plays a central role in mediating MagLMP-induced repolarization of macrophages by activating the NF-κB signaling pathway and increasing the level of glycolysis.

### Programmable MagLMP confers cyclic activation of biochemical signals and sustained repolarization of macrophages

**Fig. 4:**
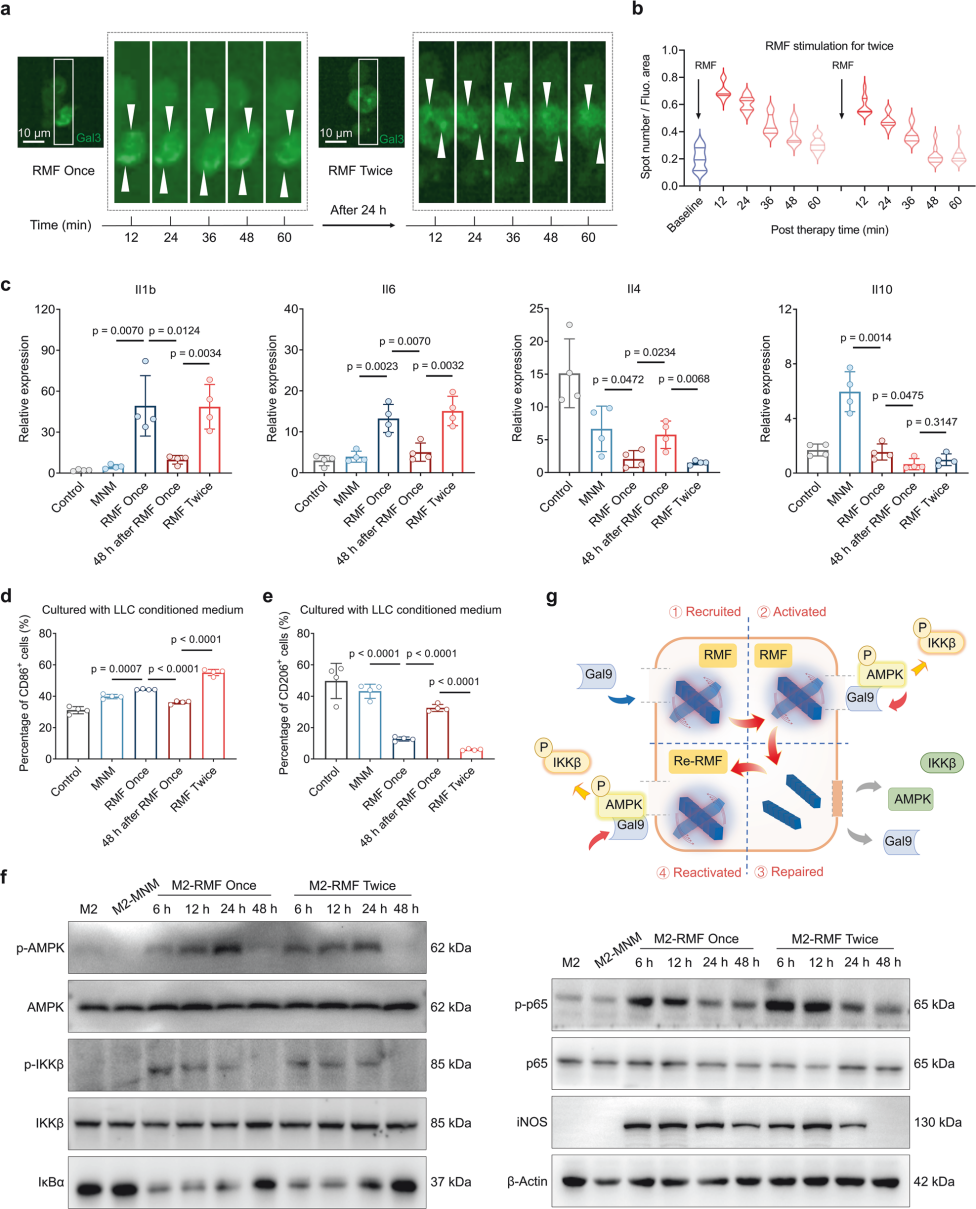
Programmable MagLMP enables cyclic activation of biochemical signals and sustained repolarization of macrophages. **a**, **b** EGFP-Gal3-expressing RAW 264.7-polarized M2 macrophages were incubated with MNMs and treated with 1 Hz RMF for 15 min (RMF once). Twenty-four hours later, the cells were treated with 1 Hz RMF again (RMF twice). Representative real-time fluorescence images (**a**) and analysis of spot number (EGFP-Gal3) per fluorescent region (**b**) are shown. At each time point, all fluorescence-positive cells in 9 wells of a 96-well plate were counted. **c**–**e** RAW 264.7-derived M0 macrophages were cultured in LLC-conditioned medium. Cells were incubated with or without MNMs and treated with or without 1 Hz RMF once or twice. mRNA levels of *Il1b*, *Il4*, *Il6*, and *Il10* were examined (**c**). CD86 (**d**) and CD206 (**e**) expression was examined by flow cytometry. Data are presented as the means ± SD. Statistical significance was defined as *P* < 0.05 (*n* = 4 independent biological replicates). **f** RAW 264.7-derived M2 macrophages were incubated with MNMs and treated with 1 Hz RMF for 15 min; 48 h later, the cells were treated with 1 Hz RMF for another 15 min. At the indicated time points, cells were harvested, and immunoblotting analysis was performed. AMPK, p-AMPK, IKKβ, p-IKKβ, p65, p-p65, and iNOS levels were analyzed by western blotting. **g** Schematic diagram of macrophage repolarization induced by programmable MagLMP, which promoted Gal9 recruitment through reversible LMP regulation, thereby activating the AMPK and NF-κB signaling pathways.

Persistent M1 repolarization of macrophages in tumor tissues is critical for mounting antitumor immunity.^40,44^ We therefore constructed a programmable MagLMP to regulate reversible lysosomal membrane damage and maintain persistent macrophage M1 repolarization. First, LysoSensor was used to characterize the acidic environment within lysosomes. Confocal imaging showed that fluorescence intensity decreased after RMF stimulation and then gradually recovered over 48 h (Supplementary information, Fig. S8a). Upon a second round of RMF stimulation, fluorescence intensity declined again, indicating that programmable MagLMP could produce cyclic membrane damage in lysosomes. Real-time fluorescence imaging of EGFP-Gal3 demonstrated that the regulatory effect of programmable MagLMP on lysosomes was repeatable (Fig. 4a, b). Moreover, a second round of RMF stimulation maintained M1-like polarization in the presence of LLC-conditioned medium, which originally induced M2-like polarization of macrophages. Both membrane-related genes of the M1 subtype (*Cd80* and *Cd86*) and inflammatory factors (*Il1b* and *Il6*) were also increased after the second round of MagLMP treatment (Fig. 4c; Supplementary information, Fig. S8b). The effects of cyclic repolarization regulated by programmable MagLMP were also confirmed by flow cytometric analysis of surface marker expression (Fig. 4d, e; Supplementary information, Fig. S8c, d).

Mechanistically, by detecting the expression of AMPK-NF-κB-related proteins, we confirmed that programmable MagLMP enabled cyclic activation of biochemical signaling pathways. In general, intracellular signaling cascades activated by external stimuli tend to diminish over time after the stimulus is withdrawn, thus avoiding prolonged activation that might disrupt cellular homeostasis. Consistent with this scenario, levels of p-AMPK, p-IKKβ, p-p65, and iNOS increased after RMF stimulation and gradually returned to baseline over time, whereas IκBα levels showed the opposite trend (Fig. 4f). Strikingly, cells treated twice with RMF induced AMPK phosphorylation and iNOS expression comparable to those observed after a single RMF treatment, suggesting that MagLMP can cyclically induce M1-like repolarization. Activation of NF-κB was also regulated repeatedly by MagLMP, manifesting as reductions in IKKβ and p65 phosphorylation and lower IκBα expression levels after MagLMP treatment (Fig. 4f). Collectively, these results show that programmable MagLMP can maintain the M1-like repolarization of macrophages by cyclic activation of the Gal9-AMPK-NF-κB signaling axis (Fig. 4g).

### Programmable MagLMP exerts an antitumor effect by modulating macrophage repolarization

**Fig. 5:**
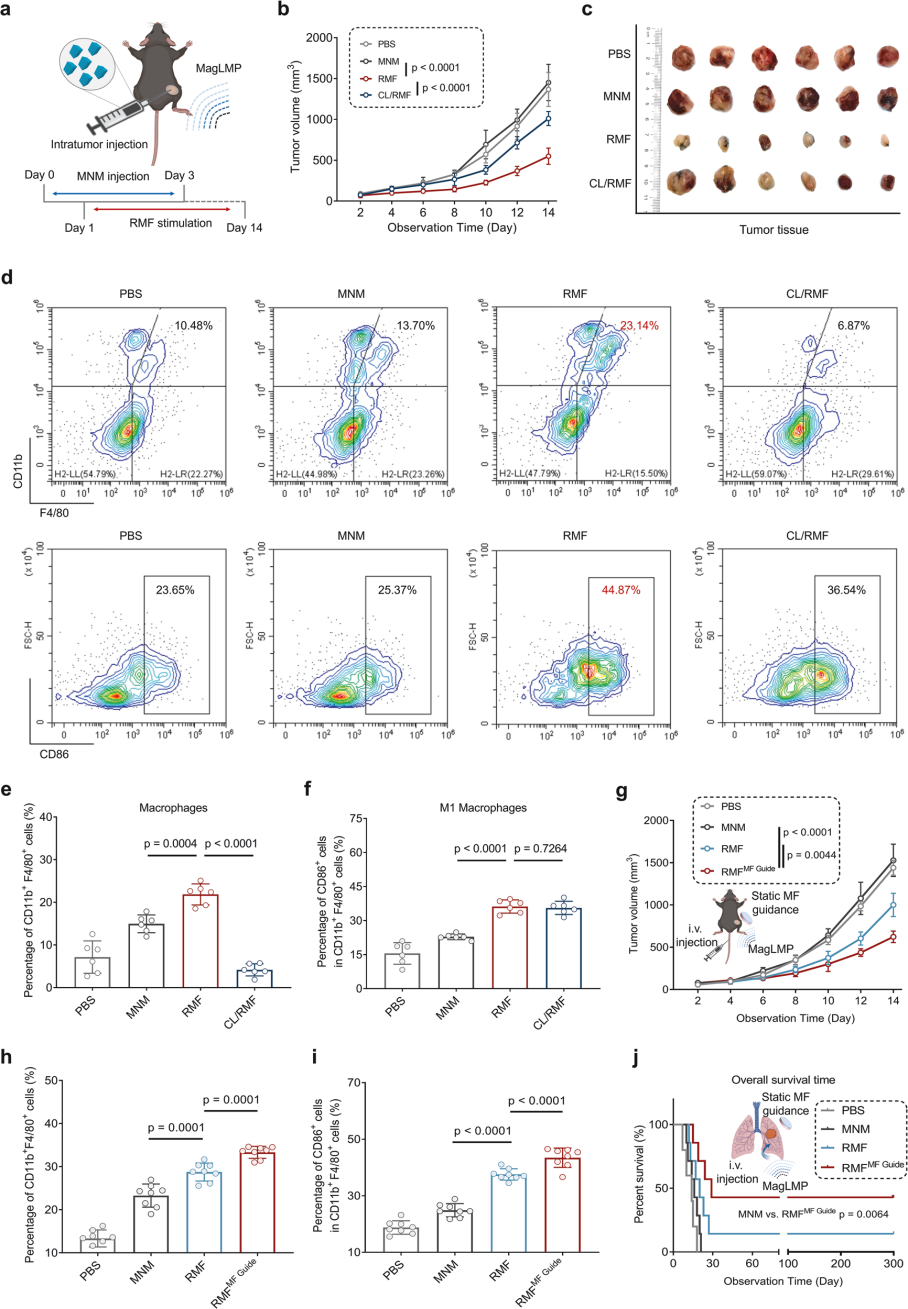
Programmable MagLMP exerts an antitumor effect by modulating macrophage repolarization. **a** Schematic diagram of the MagLMP strategy for antitumor immunity. **b**–**f** LLC cells were implanted subcutaneously into C57BL/6 mice. MNMs were injected into the tumors directly before the MagLMP strategy was applied to these mice. Mice were treated with or without CL. Tumor growth was measured over time (**b**). Fourteen days after RMF treatment (30 min per day), tumors were dissected (**c**). Flow cytometry was used to analyze macrophages (F4/80^+^CD11b^+^) and M1 macrophages (CD86^+^ in F4/80^+^CD11b^+^ cells) in tumor tissues (**d**–**f**). Data are presented as the means ± SD of six mice. Statistical significance was defined as *P* < 0.05. **g**–**i** LLC cells were implanted subcutaneously into C57BL/6 mice. MNMs were injected intravenously into the tumors, and a static magnetic field was placed over the tumor to guide MNM accumulation. The MagLMP strategy was performed on these mice for 14 days, and tumor growth was measured over time (**g**). Macrophages (F4/80^+^CD11b^+^) and M1 macrophages (CD86^+^ in F4/80^+^CD11b^+^ cells) in tumor tissues were analyzed by flow cytometry (**h**, **i**). Data are presented as the means ± SD of eight mice. Statistical significance was defined as *P* < 0.05. **j** LLC cells were implanted into the lungs of C57BL/6 mice. MNMs were injected intravenously into each tumor, and a static magnetic field was placed over the tumor to guide MNM accumulation. The MagLMP strategy was performed on these mice for 14 days. Kaplan–Meier survival analysis was performed (nine mice per group).

A murine allograft tumor model of C57BL/6 mice was established to evaluate the immunotherapeutic effects of programmable MagLMP (Fig. 5a). Importantly, a 14-day RMF treatment significantly retarded tumor growth (Fig. 5b, c). After macrophage depletion with clodronate liposomes (CLs), administered either by continuous intraperitoneal injection before treatment or by intravenous injection every three days during treatment, the antitumor effect of MagLMP was markedly impaired (Fig. 5b, c; Supplementary information, Fig. S9a–d), indicating an essential role for macrophages in MagLMP-mediated antitumor immunity. Consistent with these results, a potent antitumor effect of MagLMP was also observed in subcutaneous 4T1 and B16 tumor models (Supplementary information, Fig. S9e). To further verify the contribution of macrophages to MagLMP-induced tumor suppression, BMDMs were extracted from wild-type mice, differentiated in vitro, and co-incubated with MNMs for 24 h. These BMDMs were then intratumorally transferred into tumors that had been macrophage-depleted by three consecutive days of CL pretreatment. Tumor-associated macrophages were effectively depleted for an extended period, and the transferred BMDMs survived in vivo for over a week before gradually declining (Supplementary information, Fig. S9f, g). After 14 days of MagLMP treatment, tumors that received BMDM reinfusion exhibited a significantly restored antitumor response (Supplementary information, Fig. S9h–j). Flow cytometry analysis further demonstrated that programmed MagLMP enhanced the infiltration of macrophages and effectively repolarized macrophages to the M1-like subtype in tumor tissues. Although infiltration was significantly reduced after CL injection compared with the RMF group, a high proportion of M1-like subtype macrophages remained after MagLMP regulation (Fig. 5d–f). Detection of CD4^+^ and CD8^+^ T cell percentages in tumor and spleen tissues demonstrated that RMF stimulation increased CD8^+^ T cell infiltration, indicating that the MagLMP strategy could activate CD8^+^ T cell-mediated adaptive immunity (Supplementary information, Fig. S10a, b). This observation was further substantiated by immunofluorescence staining of tumor sections, which demonstrated upregulation of CD8^+^ T cells in the RMF-treated group (Supplementary information, Fig. S10c). OVA-overexpressing LLC tumor cells were then constructed and inoculated subcutaneously into C57BL/6 mice. Higher percentages of OVA-tetramer, CD107a, IFN-γ, and granzyme B (GZMB) in CD3^+^CD8^+^ T cells of the RMF group in the spleen demonstrated that MagLMP therapy could promote the activation of tumor-specific CD8^+^ T cells (Supplementary information, Fig. S10d). Immunofluorescence staining of tumor tissue sections showed that MagLMP resulted in a significant increase in M1-like macrophages (Supplementary information, Fig. S10e). Tumor-associated macrophages were isolated by flow cytometric sorting, and RT-qPCR revealed that RMF treatment markedly enhanced the expression of M1-related genes, indicating that MagLMP and the combination strategy could promote repolarization to the M1-like subtype (Supplementary information, Fig. S10f). We therefore concluded that programmable MagLMP exerted a powerful antitumor effect by inducing the infiltration and repolarization of macrophages and the mounting of antitumor immunity.

Next, to confirm the accumulation of MNMs in macrophages, we investigated MNM uptake efficiency in immune cells and LLC tumor cells in vitro and in vivo, as these are the major cell types that internalize MNMs in tumor tissues. Iron element quantification showed that macrophages had ~1.57-fold greater internalization of MNMs than LLC cells after 24 h of incubation in vitro (Supplementary information, Fig. S11a). Next, we evaluated the phagocytosis of MNMs in different cell types in vivo. LLC cells were implanted subcutaneously in mice to trace tumor cells, and cells containing MNMs were digested and isolated from tumors by magnetic separation, followed by flow cytometry analysis. The results showed that as the MNM injection dose increased, the proportion of MNM-loaded CD45^+^ cells increased significantly, whereas that of CD45^–^ cells progressively decreased. Among the CD45^+^ populations, macrophages and neutrophils constituted the predominant phagocytic cell types (Supplementary information, Fig. S11b, c). Further analysis of dynamic changes in cell composition over the course of treatment revealed a marked increase in CD45^+^ immune cells, with macrophages steadily increasing while neutrophils declined rapidly (Supplementary information, Fig. S11d, e), indicating that macrophages serve as the key regulatory cell population during MagLMP therapy.

We then systematically evaluated the cytotoxicity of MagLMP in mice. Element quantification and Prussian blue staining of iron in different tissues demonstrated that most MNMs remained in tumor tissues after 14 days of treatment, and few MNMs were located in the spleen and kidney (Supplementary information, Fig. S11f, g). In addition, serum levels of IL-1β and TNF-α indicated that the MagLMP strategy did not significantly influence inflammation levels (Supplementary information, Fig. S11h). Since MNMs also accumulated in tumor cells, we examined the effect of MagLMP on these cells. MagLMP showed negligible cytotoxicity for LLC tumor cells, and only frequencies above 5 Hz induced cell death (Supplementary information, Fig. S12a). A cell migration assay demonstrated that MagLMP did not significantly affect the migration of tumor cells (Supplementary information, Fig. S12b, c). RT-qPCR results suggested lower expression of genes encoding immune checkpoint proteins (*Cd47* and *Pdl1*) and invasion-related proteins (*Vegf* and *Tgfb*), reflecting a more favorable antitumor immunity environment (Supplementary information, Fig. S12d). Notably, flow cytometry analysis revealed that MHC class I expression on tumor cells was upregulated after MagLMP treatment (Supplementary information, Fig. S12e), whereas direct treatment of LLC tumor cells with MagLMP in vitro did not induce such upregulation (Supplementary information, Fig. S12f), indicating potential enhancement of tumor immunogenicity and remodeling of the tumor immune microenvironment. Furthermore, subcutaneously implanted LLC cells pre-internalized with MNMs did not show any significant effect of continuous MagLMP treatment for 14 days on tumor growth (Supplementary information, Fig. S12g).

We next examined the significant potential of MagLMP in antitumor immunity in different clinical settings. Resistance to immune checkpoint inhibitors (ICIs) (e.g., PD-1 and PD-L1 antagonists) remains a challenge in lung cancer treatment.^45^ In our constructed LLC tumor models, treatment with ICIs alone also demonstrated poor antitumor efficacy (Supplementary information, Fig. S13a, b). Interestingly, the combination of MagLMP with anti-PD-1 antibody was found to synergistically inhibit tumor growth (Supplementary information, Fig. S13a, b), suggesting that macrophage repolarization via MagLMP had a combinatory effect with immune checkpoint blockade therapy in tumor suppression. The prolonged survival time of RMF-treated mice revealed that programmable MagLMP combined with anti-PD-1 antibody (iPD-1/RMF) had a powerful antitumor effect (Supplementary information, Fig. S13c). In addition, flow cytometry analysis demonstrated that both the infiltration and the proportion of M1-like macrophages in the tumor and spleen were significantly increased in the iPD-1/RMF group (Supplementary information, Fig. S13d–f). The presence of MNMs in the spleen may contribute to the polarization of macrophages within this organ in response to RMF treatment.

We also explored the intravenous administration of MNMs, followed by application of a static magnetic field to promote their enrichment at the tumor site, thereby evaluating the feasibility and translational potential of MagLMP-mediated antitumor therapy via systemic delivery. An in vitro blood flow model using a peristaltic pump and artificial plasma showed that ~73% of MNMs could be magnetically enriched under simulated murine flow conditions at 0.5 mL/min, corresponding to large-artery flow rates (Supplementary information, Fig. S14a, b and Video S15). A Transwell assay with a confluent endothelial monolayer further confirmed that MNMs could effectively traverse the endothelial barrier under static magnetic attraction for 4 h, showing a 3.9-fold higher translocation compared with the no-MF-guide group (Supplementary information, Fig. S14c–e). After intravenous injection of MNMs in mice, iron levels in the blood returned to baseline within 4 h, while application of the static magnetic field markedly increased iron accumulation in tumor tissues (Supplementary information, Fig. S14f). In vivo and ex vivo imaging of Cy5.5-labeled MNMs revealed progressive enrichment in tumors under static magnetic guidance, with significantly stronger fluorescence signals at 4 h compared with the no-MF-guide group (Supplementary information, Fig. S14g–j). We next observed the antitumor therapeutic effect of the MagLMP strategy after intravenous injection of MNMs (Fig. 5g). Importantly, after the static magnetic field was used to guide the enrichment of MNMs at tumor sites, the inhibition of tumor growth was significantly improved by MagLMP (Fig. 5g).

The activation of macrophages was also confirmed in the intravenous injection and MF-guided MagLMP treatment model. Flow cytometry analysis showed that the levels of macrophages and M1-like macrophages increased after magnetic field guidance combined with MagLMP treatment (Fig. 5h, i). In addition, a time-dependent increase in CD45^+^ immune cells was observed in the intravenous injection model, among which macrophages accounted for over 30%, indicating that they served as the predominant effector cells in the MagLMP response (Supplementary information, Fig. S14k, l). M1-like macrophages also increased progressively with continued MagLMP treatment (Supplementary information, Fig. S14m). To further investigate whether macrophages internalized MNMs after MF guidance, tumors were harvested 4 h post-injection and magnetic separation, followed by flow cytometric sorting of tumor-associated macrophages (CD45^+^CD11b^+^F4/80^+^). Inductively coupled plasma mass spectrometry (ICP-MS) quantification revealed significant MNM accumulation within the sorted macrophages (Supplementary information, Fig. S14n). Moreover, RT-qPCR analysis confirmed that RMF stimulation of these macrophages ex vivo led to marked upregulation of M1 marker genes, including *Cd80*, *Cd86*, *Il1b*, and *Il6*, in the intravenous injection-RMF^MF guide^ group, comparable to the response seen in the intratumoral injection-RMF group (Supplementary information, Fig. S14o). We constructed an adenocarcinoma in situ (AIS) of the lung in the mouse model by injecting LLC cells into the lung tissues. MNMs were injected intravenously and then guided into the lung tissue by magnetic fields. After programmable MagLMP, the overall survival of mice was significantly prolonged. One third of the mice showed a survival benefit of over 300 days, compared with a maximum survival time of only 21 days for the MNM group (Fig. 5j), demonstrating the effectiveness of MagLMP for the treatment of in situ lung cancer. Together, these results suggest that MagLMP has immense potential to provide powerful antitumor immunity effects through different routes of MNM administration.

### MagLMP directs macrophage repolarization by activating the Gal9-AMPK axis for antitumor therapy

**Fig. 6:**
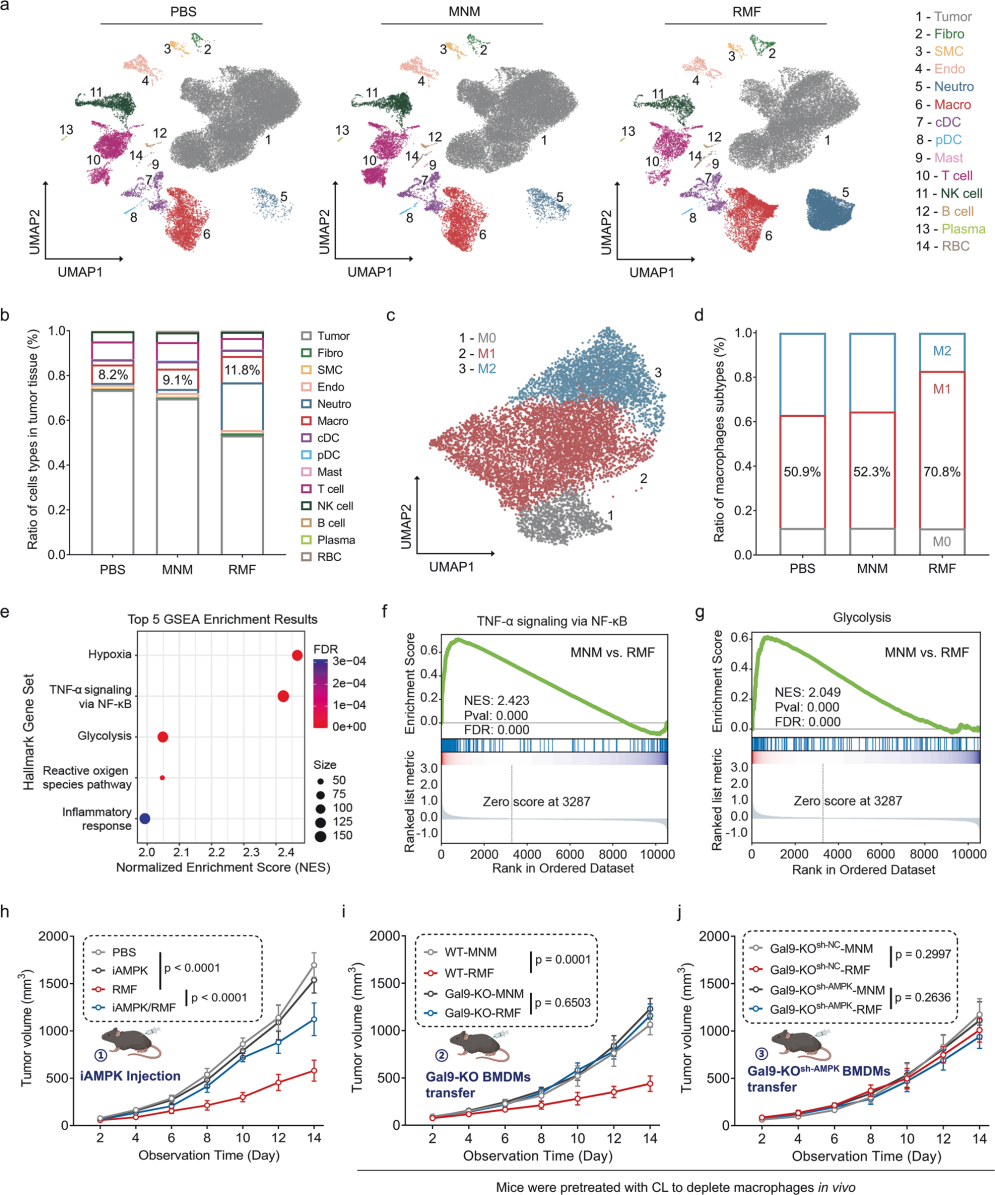
MagLMP induces macrophage repolarization by activating the Gal9-AMPK axis for antitumor therapy. **a**–**g** LLC cells were implanted subcutaneously into C57BL/6 mice. MNMs were injected into the tumor directly before the MagLMP strategy was performed. scRNA-seq was performed on these tumors. UMAP plots and the ratios of major cell types in tumor tissues are shown (**a**, **b**). A UMAP plot and the ratios of different macrophage subtypes are shown (**c**, **d**). Pathway enrichment analysis of differentially expressed genes in macrophages from tumor tissues with RMF treatment compared with MNM treatment (**e**). Ranked list metric and enrichment scores of the NF-κB signaling pathway and glycolysis in macrophages of tumor tissues (**f**, **g**). Data are presented as the means of three mice per group. **h** Mouse-derived allografts of LLC cells were dissected and implanted subcutaneously into wild-type C57BL/6 mice. Mice were treated with or without RMF (30 min per day) and/or an AMPK inhibitor (20 mg/kg per mouse every two days). Tumor growth was measured over time. Data are presented as the means ± SD of six mice. Statistical significance was defined as *P* < 0.05. **i** Mouse-derived allografts of LLC cells were dissected and implanted subcutaneously into C57BL/6 mice. BMDMs were isolated and differentiated from wild-type and *Gal9*-KO mice, then co-incubated with MNMs for 24 h before adoptive transfer into tumor tissues. Mice were pretreated with CL and then stimulated with or without RMF (30 min per day). Tumor growth was measured over time. Data are presented as the means ± SD of six mice. Statistical significance was defined as *P* < 0.05. **j** Mouse-derived allografts of LLC cells were dissected and implanted subcutaneously into C57BL/6 mice. BMDMs were isolated and differentiated from *Gal9*-KO mice, then transduced with lentiviruses encoding either NC- or AMPK-shRNA. Cells were co-incubated with MNMs for 24 h before adoptive transfer into tumor tissues. Mice were pretreated with CL and then stimulated with or without RMF (30 min per day). Tumor growth was measured over time. Data are presented as the means ± SD of six mice. Statistical significance was defined as *P* < 0.05.

To confirm the effect of MagLMP-induced macrophage repolarization in the tumor microenvironment, we performed scRNA-seq on all cells within tumors isolated from C57BL/6 mice treated with PBS, MNM, or MagLMP. Cells were clustered into 30 subsets by Seurat (Fig. 6a). Compared with PBS or MNM treatment, MagLMP treatment dramatically reduced the tumor cell population and increased the population of macrophages (*Cd68*, *Apoe*, *C1qc*, *Ctss*) (Fig. 6b). Macrophages are comprised of M0, M1, and M2 subtypes. M1-like macrophages are predominantly involved in pro-inflammatory responses, whereas M2-like macrophages primarily participate in anti-inflammatory responses.^46^ Therefore, the macrophages in tumors were further clustered into 3 subsets: M0 (*Adgre5*, *Cd300a*, *Itgal*, *Mgst1*), M1 (*Tnf*, *Cd80*, *Il1b*, *Ifitm1*), and M2 (*Ctsb*, *Mrc1*, *Trem2*, *Cd81*) (Fig. 6c). The data showed that MagLMP treatment markedly increased the proportion of the M1 subtype and reduced that of the M2 subtype (Fig. 6d). Notably, immune subtype profiling of T cells and neutrophils revealed a significant increase in effector CD8^+^ T cells (*Cd8a*, *Gzma*, *Gzmk*) and antitumor-phenotype neutrophils (*S100a8*, *Irf1*) in the RMF-treated group (Supplementary information, Fig. S15a, b). Gene set enrichment analysis (GSEA) of differentially expressed genes in macrophages revealed that the NF-κB signaling pathway and glycolysis were significantly upregulated after MagLMP treatment (Fig. 6e–g).

We next isolated tumor-associated macrophages by flow cytometric sorting and measured AMPK and iNOS protein levels. The results showed that both MagLMP and the combination strategy promoted AMPK phosphorylation and iNOS expression (Supplementary information, Fig. S15c–e). To confirm the contribution of the Gal9-AMPK axis to the antitumor effect of MagLMP in vivo, we blocked AMPK and Gal9. Intraperitoneal injection of Compound C significantly impaired the antitumor effect of RMF in vivo, suggesting that the antitumor effect of programmable MagLMP was dependent on AMPK activation (Fig. 6h; Supplementary information, Fig. S15f, g). To further assess the functional relevance of Gal9 in vivo, bone marrow cells from *Gal9*-KO mice were extracted and differentiated into BMDMs. After co-incubation with MNMs in vitro, these Gal9-deficient macrophages were adoptively transferred into tumors in which endogenous macrophages had been depleted. In contrast to BMDMs derived from wild-type mice, *Gal9*-KO BMDMs failed to suppress tumor growth after 14 days of RMF treatment, indicating that Gal9 was essential for the antitumor effect of MagLMP in vivo (Fig. 6i; Supplementary information, Fig. S15h, i). When *Gal9*-KO BMDMs were transduced with lentiviruses encoding shRNA targeting AMPK in vitro prior to reinfusion (Supplementary information, Fig. S15j, k), no further reduction in tumor suppression was observed compared with *Gal9*-KO alone, supporting the notion that both Gal9 and AMPK are indispensable mediators of MagLMP-induced antitumor effects (Fig. 6j; Supplementary information, Fig. S15l–n). In summary, we conclude that the Gal9-AMPK axis plays a critical role in the antitumor effect of MagLMP by directing macrophage repolarization.

## Discussion

### Dynamic mechanical regulation of biochemical signals at the organelle level

Extracellular mechanical signals can be transduced into biochemical signals by regulating mechanosensitive channels and cell adhesion molecules on the plasma membrane to modulate immune cell functions.^16–19^ Conventional extracellular mechanical stimulation techniques, such as optical tweezers and AFM, are limited in their ability to achieve mechanical regulation in vivo.^14,15^ Furthermore, the precision of subcellular-level mechanical regulation remains limited, and the mechanisms underlying signal transduction are unclear. Considering the roles of organelles in mechanosensing and signal transduction,^20,22^ we developed the MagLMP strategy to extend mechanical regulation from the extracellular level to the organelle level for in vivo applications, enabling precise mechanical stimulation within lysosomes through natural lysosomal targeting of MNMs.

The self-assembly of magnetic nanoparticles under static or dynamic magnetic fields has been reported, demonstrating efficacy in molecular or biological identification, antibody delivery, and antitumor therapy.^47–50^ In our study, we leveraged the advantages of self-assembling magnetic nanomotors, using them to respond to RMF and generate frequency-dependent dynamic magneto-mechanical forces within lysosomes. In addition, we confirmed that magnetic field strength is critical for promoting the rotational motion of MNM assemblies. Only when the magnetic field strength is large enough can the magnetic torque exerted by the magnetic field on the MNMs effectively overcome the resistance torque exerted by the lysosomal fluid, driving the MNMs to generate continuous and stable rotational motion. In our experiment, a field strength of 260 mT was sufficient to drive efficient intracellular rotation of MNMs within lysosomes under different frequencies, thereby enabling the generation of cyclic mechanical force. More importantly, this approach enabled reversible regulation of lysosomal damage to mediate Gal9-AMPK activation and thus induce sustained macrophage repolarization. Notably, several studies have linked AMPK to anti-inflammatory responses in macrophages,^51^ and our results support its context-dependent role: under lysosomal stress triggered by magneto-mechanical force, AMPK phosphorylation promotes NF-κB activation and M1-like macrophage repolarization. This highlights a distinct immune regulatory mechanism that acts through organelle-specific mechanical cues. Furthermore, dynamic MagLMP enables precise manipulation of Gal9-AMPK-NF-κB and cyclic activation of NF-κB signals, providing an innovative approach for the manipulation of biochemical signals through intracellular mechanical force.

The magnetic field frequency-dependent regulation of lysosomal membrane damage is another important finding of this study. We discovered a strong link between the frequency-dependent vortex effect and lysosomal membrane damage through continuous observation of Gal3 fluorescent foci, which serve as markers of lysosomal membrane damage.^26,27^ We then performed simulation predictions on the magnetic mechanical forces within lysosomes. The lysosome has a complex molecular structure that affects the rotational motion of the assembled MNMs, making precise modeling in simulations challenging. To reflect the lysosomal environment, we attempted to extract lysosomal contents and measured their average viscosity to enable more accurate simulation results. We observed that a rotation frequency from 0.8 to 2 Hz induced the assembled MNMs to produce a shear stress of ~1 Pa on the lysosomal membrane. Within this range, MagLMP not only triggered signal transduction mediated by LMP but also produced reversible damage to the lysosomal membrane, thus minimizing cytotoxicity and enabling dynamic regulation of mechanical forces at the organelle level.

### Dynamic and sustained immune regulation for antitumor treatment

In the immunosuppressive tumor microenvironment, immune cells are constantly stimulated by inhibitory signals.^40^ Therefore, dynamic and sustained immune regulation is necessary to enhance the effects of antitumor treatment. Small molecules have shown the ability to regulate immune cells,^23,52^ and iron-based magnetic nanoparticles enhanced intracellular levels of reactive oxygen species through the Fenton reaction to induce macrophage reprogramming.^53^ However, these regulatory approaches are insufficient to sustain macrophage polarization, making it difficult to generate durable antitumor effects. The metabolism of biochemical drugs or ions can reduce their ability to induce macrophage polarization. The MagLMP strategy proposed in our study avoids these limitations. Through the use of active mechanical forces to induce reversible lysosomal membrane damage, efficient and sustained M1 repolarization effects could be achieved compared with those obtained with MNMs alone (as shown in Fig. 2g). For instance, intravenous administration of the Food and Drug Administration (FDA)-approved iron oxide nanoparticle ferumoxytol to treat liver metastases from lung cancer resulted in only ~1% increase in M1-like macrophages after 21 days.^53^ By contrast, MagLMP combined with magnetic guidance increased the content of M1-phenotype macrophages by ~20% in a subcutaneous tumor-bearing model. We also clearly demonstrated the potent antitumor efficacy of MagLMP in an intravenous injection model. By strategically using the static magnetic field at the tumor site, we significantly enhanced the local accumulation of MNMs within tumor tissue. This approach also demonstrated a long-term survival benefit in treating AIS of the lung in the mouse model. The effects of this controlled immune-regulation strategy surpass the transient effects of conventional regulation by biochemical drugs or nanomedicines, thus showing substantial promise for use in durable antitumor immunity.

### Potential benefits of a combined mechanical–biochemical therapeutic strategy

The MagLMP strategy shows significant potential for use in antitumor immunotherapy. However, relying solely on magneto-mechanical force to activate immune antitumor responses has yet to achieve optimal therapeutic efficacy. Notably, the effects of intracellular mechanical forces and biochemical drugs can be complementary, with the former providing controlled and sustainable immune activation while the latter offer rapid molecular-level responses. Together, they hold great promise for enhancing the efficacy of cancer immunotherapy. Here, we combined programmable MagLMP with PD-1 inhibitors for the treatment of PD-1 inhibitor-resistant lung adenocarcinoma tumor models.^9^ Our findings suggest that the combination of MagLMP and PD-1 inhibitors can lead to more effective tumor suppression.

However, the strategy did not show more significant synergistic effects, indicating that T cells may not play a primary role in MagLMP-induced antitumor therapy. The results of scRNA-seq suggested that there was no overall increase in total CD8^+^ T cells after RMF stimulation, although there was a higher proportion of effector CD8^+^ T cells. Flow cytometry analysis and immunofluorescence staining revealed a moderate upregulation of CD8^+^ T cells, which differed slightly from the scRNA-seq results. We attribute this discrepancy to differences between protein-level and mRNA-level detection, which are not contradictory. Because protein expression more closely reflects cellular function, we regard the results of flow cytometry and immunofluorescence staining as the gold standard for assessing T cell function. We observed recruitment and significant repolarization of macrophages, as well as greater numbers and activation (Irf1-positive) of neutrophils. It is known that neutrophils can be widely recruited by chemokine-secreting macrophages.^54^ Recent reports suggest that neutrophils may also exert unique anti-tumor effects by secreting IFN-γ, enabling them to eliminate tumor cells that evade T cell immune recognition.^55^ The substantial recruitment of neutrophils suggests that they may have a prominent role in the MagLMP strategy for antitumor therapy, a possibility that warrants further investigation.

The therapeutic benefits could presumably be improved by enhancing the penetration and specific macrophage targeting of the nanomotors in tumor tissues. To promote deep penetration, our team developed a three-dimensional rotating field device that combines translational and rotational modes, increasing the degrees of freedom from 2 to 5. This allows for targeted movement and deep tissue penetration in tumors. With respect to targeting specificity, we clearly demonstrated the predominant enrichment of MNMs in macrophages and the capacity of MagLMP to repolarize macrophages and exert antitumor effects in vitro and in vivo. As a next step, modification of nanomotors with macrophage-targeted antibodies would be a practical approach to promote specific recognition by macrophages. We will also attempt to optimize the intrinsic properties of the nanomotors, such as by constructing a bacteria-mimicking morphology, to enhance macrophage recognition and internalization. Nonetheless, it should be noted that approximately 10% of the MNMs were internalized by dendritic cells (DCs). As another essential type of antigen-presenting cell, DCs may have important functional roles in tumor growth, and the potential effect of MagLMP on DCs and other immune cell subsets warrants further investigation.

### Further development of the MagLMP strategy

**Fig. 7:**
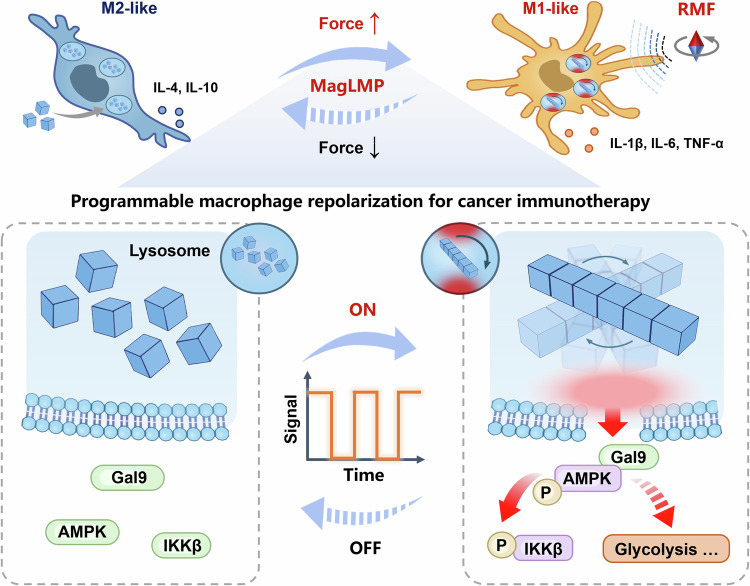
The MagLMP strategy induces programmable macrophage repolarization for cancer immunotherapy. Magnetic nanomotors exhibit intrinsic lysosomal targeting and respond to magnetic fields, enabling them to self-assemble within lysosomes and precisely generate magneto-mechanical forces under a rotating magnetic field. Our proposed cyclic MagLMP strategy enables reversible induction of lysosomal membrane damage and cyclic activation of Gal9-AMPK-NF-κB signaling, as well as metabolic alterations, by precisely controlling the mechanical force output. This strategy efficiently induces sustained M1-like macrophage repolarization, followed by the mounting of antitumor immunity in vivo.

We have developed a dynamic regulation platform capable of inducing programmable mechanotransduction at the organelle level. This platform causes reversible damage to lysosomal membranes and enables cyclic regulation of biochemical signals in macrophages. It also offers insights into the underlying mechanical regulation and clarifies the mechanisms of signal transduction at the organelle level (Fig. 7). We successfully applied this mechano-regulated antitumor approach in vivo for the first time, paving the way for sustainable tumor immunotherapy. Looking forward, we anticipate that this tool will enable the precise regulation of other organelles and intracellular biochemical signaling molecules beyond lysosomes. It holds promise as a pioneering strategy for modulating intracellular signals effectively following biochemical drugs.

## Materials and methods

### Cell lines

For macrophages, the RAW 264.7 cell line was purchased from Shanghai Yu Chun Biotechnology. The THP-1 and *Gal9*-KO THP-1 cell lines were provided by Prof. Haipeng Liu at Shanghai Pulmonary Hospital.^56^ Murine BMDMs were generated from bone marrow cells with DMEM (Biosharp, BL304A) complete medium plus 20 ng/mL M-CSF (Novoprotein, CB34) for 7 days. To obtain different subtypes of macrophages, RAW 264.7, THP-1, or BMDMs were incubated with 10 pg/mL LPS (Beyotime Biotech, ST1470) and 20 ng/mL IFN-γ (Peprotech, 210-13-10) to polarize them into M1 subtypes or incubated with 20 ng/mL IL-4 (Peprotech, 214-14-20) and 20 ng/mL IL-13 (Peprotech, 315-05-20) for 48 h to polarize them into M2 subtypes. For tumor cells, the LLC cell line (mouse, male) was provided by Prof. Haipeng Liu. OVA-overexpressing LLC cells (OVA-LLC) were provided by Prof. Weiwei Yang. All cells were cultured in a 5% CO_2_ incubator at 37 °C with complete medium (glucose-supplemented DMEM with 10% FBS, L-glutamine, and penicillin/streptomycin) and tested annually for mycoplasma contamination.

### Mice

C57BL/6 mice (male, 18–20 g) were purchased from Slake Experimental Animal Company (Shanghai, China). *Gal9*-KO C57BL/6 mice were provided by Prof. Haipeng Liu.^56^ All mice were bred in the specific pathogen-free (SPF) animal facility of the Laboratory Animal Center of Tongji University in individually ventilated cages (room temperature, 21 ± 1 °C; relative humidity, 40%–70%, and a 12-h light–dark cycle) and had access to food and water ad libitum. Tumor-bearing mice were constructed by subcutaneous injection of tumor cells (1 × 10^6^ cells per mouse) into the flanks. All mice were selected and grouped randomly and observed within ethical limits. All animal experiments were approved and performed under the guidance of the Institutional Animal Care and Use Committee at Tongji University (Shanghai, China).

### Magnetic field device

The RMF device was independently developed by our team. The RMF was generated by two rotating NdFeB magnets (the device was fabricated by Chuanshanjia Co., Ltd., Shanghai, China), which were controlled by a motor to adjust the rotation at different frequencies. In addition, the MFG-100 magnetic field system (MagnebotiX AG), controlled by electromagnetic coils and integrated with an inverted fluorescence microscope (Olympus), was used for in vitro video recording of MNM rotational motion. Finally, a custom-built electromagnetic coil system with a field strength of 20 mT, combined with a high-resolution microscope (Nikon), was used for real-time imaging of MNM rotational motions inside cells.

### Preparation of MNMs and fluorescent molecule-labeled MNMs

#### Synthesis of 25-nm zinc-doped iron oxide MNPs

MNPs were synthesized in the organic solution phase by the thermal decomposition method based on previous work.^57^ Iron (III) acetylacetonate (Fe(acac)_3_, 97%, 282.5 mg), zinc acetylacetonate hydrate (Zn(acac)_2_, 97%, 316.3 mg), and 4-phenylbenzoic acid (99%, 400 mg) were dissolved in a mixture of dibenzyl ether (> 98%, 10.4 mL) and oleic acid (AR, 1.2 mL). The solution was sonicated for 10 min for dispersal, followed by argon aeration for 30 min with stirring at 400 rpm. The solution was then heated to 290 °C and held for 30 min with aeration and stirring. After the solution had cooled to room temperature, 10 mL of ethanol was added to promote the precipitation of MNPs. The products were washed sequentially with ethanol and toluene three times each. Finally, the MNPs were dispersed in ethanol.

#### Synthesis of MNMs (MNPs coated with PLL)

MNMs were synthesized by modifying the surfaces of MNPs with PLL to transform them from hydrophobic to hydrophilic, thus improving biocompatibility and cell internalization. In brief, 100 mg of PLL was dissolved in 4 mL of ddH_2_O and mixed with 8 mL of ethanol containing 10 mg of MNPs. The mixture was sonicated using an ultrasonic probe (model 120, Fisher Scientific, USA) with 40 W of power (working time 5 s, interval time 2 s) for 120 min to promote coating of PLL onto the MNP surfaces. The MNMs were then washed with ddH_2_O three times and finally dispersed in ddH_2_O.

#### Synthesis of MNM-FITC and MNM-Cy5

MNMs were labeled by chemically coupling the fluorescent molecule fluorescein isothiocyanate (FITC) onto their surfaces to facilitate detection of their intracellular localization. First, 2 mg of MNMs were dispersed in 2 mL ddH_2_O, and 2 mL of ethanol containing 0.02 mg of FITC was added. After ultrasonic sonication for 30 min, the mixture was stirred overnight. The resulting MNM-FITC particles were collected by magnetic separation and washed with ethanol and ddH_2_O three times each to remove free FITC.

The fluorescent molecule Cy5 carrying a carboxyl group was also chemically coupled to the surfaces of MNMs. In brief, 10 mg of EDC and 10 mg of NHS were dissolved in 5 mL PBS (pH 5.5), followed by the addition of 100 μg Cy5 and sonication by ultrasound for 2 h. Two milligrams of MNMs were then added, and the mixture was magnetically stirred for 12 h. The resulting MNM-Cy5 particles were collected by magnetic separation and washed with ddH_2_O three times.

### Characterization of MNMs

The morphology and elemental composition of MNMs were characterized by transmission electron microscopy (TEM, JEM-1230, JEOL Ltd.). The hydrodynamic size and zeta potential of MNMs were measured by dynamic and electrophoretic light scattering (Zetasizer Nano ZS90, Malvern Ltd.) dispersed in ddH_2_O with a nanoparticle concentration of 10 μg/mL. The magnetic properties of dry MNMs were characterized by magnetization vs applied magnetic field (M vs H) curves at room temperature as measured with a vibrating sample magnetometer (VSM, Lakeshore 7407, USA).

### Biocompatibility of MNMs and RMF in vitro

Before RMF treatment, the biocompatibility of MNMs was evaluated by a CCK-8 assay (Beyotime Biotech, P0012). MNMs were first sterilized under UV-light irradiation for 30 min, then dispersed into DMEM at concentrations of 10 μg/mL, 20 μg/mL, and 40 μg/mL. Next, 5 × 10^3^ cells were seeded into a 96-well plate and incubated at 37 °C overnight. Fresh medium containing different concentrations of MNMs was added to replace the culture medium in each well. Medium without MNMs served as the control group. After incubation for 24 h, the CCK-8 assay was performed, and absorbance was measured at 450 nm using a microtiter plate reader (ELx808, BioTek).

For RMF treatment, cell viability was again measured by the CCK-8 assay. After incubation with 20 μg/mL MNMs for 24 h, the cells were washed with PBS three times to remove MNMs that had not been taken up into cells. The cells were then exposed to RMF at different frequencies and for different time intervals. After an additional incubation for 4 h, the CCK-8 assay was performed. To assess the effect of dynamic RMF stimulation on cell viability, cells were exposed to RMF (1 Hz, 15 min) once daily for 7 consecutive days. CCK-8 assays were performed on days 1, 3, 5, and 7, and absorbance was measured at 450 nm using a microtiter plate reader.

To observe the content of MNMs in macrophages, the iron concentration was detected. Macrophages were seeded onto dishes (35 mm Φ) at a concentration of 1 × 10^5^ cells per dish and co-cultured with MNMs at concentrations of 10 μg/mL, 20 μg/mL, and 40 μg/mL for 24 h. The cells were homogenized and lysed in aqua regia after digestion and collection. The amount of Fe in different groups was determined by ICP-MS.

### Theoretical calculation and finite element simulation within lysosomes

The assembled number of MNMs within lysosomes and the torque generated under RMF stimulation were first determined through theoretical calculations.^58–61^ A deep learning system was then used to quantify the number of assembled nanomotors in Bio-TEM images, thus providing experimental validation for the theoretical predictions. In parallel, finite-element simulations were performed to assess the changes in lysosomal membrane pressure induced by MagLMP at different magnetic field frequencies. To obtain more accurate lysosomal mechanical simulation parameters, lysosomal biophysical characteristics in macrophages were measured experimentally. Detailed procedures are described in Supplementary information, Materials and Methods.

### Preparation of SUVs and SUV-MNMs and sulforhodamine B (SRB) release assay under RMF stimulation

On the basis of previous work,^38^ fluorophore-filled SUVs and fluorophore-filled small magnetic lipid vesicles (SUV-MNMs) were prepared by the thin-film rehydration method. For SUV preparation, DOPC (10 mM, 700 µL) and DOPE (10 mM, 300 µL) were mixed and dissolved in chloroform, then added to a 10-mL round-bottom flask. The solution was rotated in a rotary evaporator (RV 10 Digital, IKA, Germany) at 40 °C for 20 min to remove the chloroform and form a lipid film, then dried overnight at room temperature. After 30 min of ultrasound, the solution was frozen and thawed 5 times using liquid nitrogen and a 50 °C water bath. The SUVs were extruded 25 times through a 200-nm polycarbonate membrane (Avanti Polar Lipids, USA) using an extrusion kit (Avanti Polar Lipids, USA), then purified using a PBS-filled NAP-5 column (GE Healthcare, UK). The purified SUVs were equilibrated at 4 °C overnight. SUV-MNMs were prepared as described for SUVs, except that the buffer of the lipid film was replaced with PBS containing 50 mM SRB (Rhawn, 3520-42-1) and 200 µg/mL MNMs. Both the SUVs and SUV-MNMs were stored at 4 °C in the dark and used within 48 h. The diameters of SUVs and SUV-MNMs were determined by DLS with a nanoparticle potentiometer (Zeta SIZER NANO ZS90, Malvern) and then negatively stained with 2% sodium phosphotungstate for characterization by TEM (JEM-1230, JEOL).

To observe the proportion of SRB released under different frequencies of RMF stimulation, 100-µL aliquots of SUVs and SUV-MNMs were added to a 96-well plate, and the fluorescence intensity was measured at 586 nm (after excitation at 565 nm) using a multimode microplate reader (Tecan, Germany). This measurement provided the baseline fluorescence intensity, owing to the fact that the fluorescence of high SRB concentrations inside SUVs is self-quenching. Next, we collected and stimulated the 100 µL SUVs and SUV-MNMs with RMF at frequencies of 0.2 Hz, 0.8 Hz, 1 Hz, 2 Hz, and 5 Hz for 15 min to induce the release of SRB by mechanically mediated vesicle damage and then measured fluorescence intensity again. Finally, the solutions were collected and freeze-thawed twice in liquid nitrogen and a 50 °C water bath to enable maximum SRB release, and the maximum fluorescence intensity was measured.

### Cell transfection

Two plasmids, EGFP-Gal3 (provided by Dr. Bin Liu at Novo Nordisk in Denmark) and YFP-Gal9 (provided by Prof. Haipeng Liu), were used to characterize LMP and the localization of Gal9. RAW 264.7 cells and BMDMs were first seeded onto 35-mm confocal dishes (1 × 10^5^ cells per dish), cultured for 24 h, and transfected with 2.5 μg plasmid using 4 μL jetPRIME transfection reagent (Polyplus, 1010000027) per dish for 24 h to 48 h. Two milliliters of 20 µg/mL MNMs-Cy5 were incubated with the cells for 24 h, and the cells were then stimulated under 1 Hz RMF for 15 min and washed three times with PBS. Lysosomes were stained with LysoTracker Red reagent (Beyotime Biotech, C1046) in DMEM at a dilution of 1:1000 for 30 min at 37 °C, and nuclei were stained with Hoechst (blue) in DMEM at a dilution of 1:500 for 15 min at 37 °C. Confocal laser scanning microscopy (TCS SP8, Leica) was used to capture fluorescence images after cells had been fixed in 4% PFA for 15 min. The Opera Phenix High-Content Screening System (PerkinElmer, USA) was used to continuously observe and analyze changes in the intensity of Gal3 fluorescent spots.

### Real-time observation of MNM motion

To observe the assembly and rotation of MNMs in vitro, MNMs dispersed in ddH_2_O were continuously imaged using the MFG-100 magnetic field system. To visualize the fluid vortices induced by MNM rotation, 500 nm polypropylene nanospheres were added as tracer particles to monitor flow behavior around the rotating MNM assemblies. To visualize the rotational motion of MNMs within lysosomes of macrophages, RAW 264.7 cells were transfected with the EGFP-Gal3 plasmid and incubated with 20 µg/mL MNMs for 24 h. Lysosomes and nuclei were stained using LysoTracker Red and Hoechst, respectively, as described above. A custom-built high-resolution fluorescence microscope integrated with a magnetic control system was used to observe the self-assembly and rotational motion of MNMs within lysosomes under applied RMF at 4, 8, 12, and 20 mT.

To evaluate magnetically guided enrichment of MNMs under flow conditions, a peristaltic pump (Henghe Fluid Electronics Technology) and artificial blood were used to simulate blood circulation in vitro. Flow rates were set to 0.1 mL/min, 0.3 mL/min, and 0.5 mL/min. One milliliter of artificial blood solution containing MNMs (50 µg/mL) was flowed through a silicone tube, and a 1-cm round magnet was placed adjacent to the tube. After each flow cycle, ICP-MS was performed to quantify the Fe content.

### Fluorescence imaging

Both macrophages and tumor cells were incubated with 20 µg/mL MNMs or MNMs tagged with fluorescent molecules (MNM-FITCs and MNM-Cy5s) for 24 h to characterize their uptake ability. Macrophages were first induced with 20 µg/mL IL-4 and 20 µg/mL IL-13 for 48 h to polarize them into the M2 subtype. MNMs were incubated with M2 macrophages for 4 h, 8 h, 12 h, and 24 h, and lysosomes and nuclei were stained with LysoTracker Red (red) and Hoechst (blue) as described above. Confocal laser scanning microscopy (TCS SP8, Leica) was used to photograph the co-localization of MNMs and lysosomes in macrophages. For 3D fluorescence confocal imaging, macrophages were co-incubated with MNMs for 2 h and 24 h. After staining with LysoTracker Red and Hoechst, *z*-stack imaging was performed to visualize the spatial co-localization of MNMs and lysosomes.

To detect changes in lysosome pH of macrophages stimulated by RMF, macrophages were seeded on 35-mm confocal dishes at a concentration of 1 × 10^5^ cells per dish and cultured for 24 h. As a positive control, 10 µg/mL CQ was used to stimulate cells for 1 h before stimulation with 1 Hz RMF for 15 min, then cultured for 0 h and 24 h. For the RMF-twice group, cells were stimulated again with 1 Hz RMF 24 h after the first RMF stimulation. All cells were washed three times with PBS. The LysoSensor probe (Yisheng Biotechnology) was used to measure lysosome pH at a 1:1000 dilution in DMEM for 30 min at 37 °C. Lysosomes and nuclei were stained as described above. After the cells were washed with PBS, confocal laser scanning microscopy (TCS SP8, Leica) was used to capture fluorescence images of the cells, and the images were analyzed using ImageJ.

To observe the effects of RMF stimulation on mitochondrial and nuclear membranes, fluorescent staining was performed using corresponding dyes. Macrophages were seeded on 35-mm confocal dishes, and MNMs were added and co-cultured for 24 h. After stimulation with an RMF of 1 Hz, the cells were washed three times with PBS, stained with mitochondrial staining probes (JC-1, C2006; TMRE, C2001S; Beyotime Biotech) according to the manufacturer’s instructions, and fixed in 4% PFA for 15 min. To stain the nuclear membranes of macrophages, the cells were directly fixed in 4% PFA for 15 min after RMF stimulation, then stained for immunofluorescence with anti-mouse Lamin B1 (Proteintech, 12987-1-AP). Cells were observed by confocal laser scanning microscopy (TCS SP8, Leica).

To directly observe the rotational motion of the assembled magnetic nanomotors within lysosomes, macrophages were seeded on 35-mm confocal dishes, and MNMs were added for 24 h. Lysosomes and nuclei were stained with LysoTracker Red and Hoechst (blue) as described above. The independently developed magnetic control devices and high-resolution fluorescence microscope (Nikon, ECLIPSE Ti2) were successfully assembled. Continuous images of the rotating magnetic nanomotors within lysosomes were directly observed under RMF stimulation.

### RT-qPCR detection

Macrophages were seeded on 35-mm dishes at a density of 1 × 10^5^ cells per dish and cultured for 24 h. After exposure to different treatment conditions, RNA was extracted from cells using 1 mL TRIzol reagent (Ambion, 15596018). Then, 200 μL trichloromethane was added, and the mixture was shaken for 15 s. After centrifugation at 15,000 rpm for 10 min at 4 °C, the upper layer of clear solution was collected. Isopropyl alcohol (500 μL) was used to precipitate the RNA, and the mixed solution was allowed to stand for 10 min at 4 °C. After centrifugation under the same conditions, the pellet was retained and washed with 1 mL 75% ethanol. Total RNA (1 μg) was reverse transcribed into complementary DNA using the ReverTra Ace qPCR RT Master Mix kit (TOYOBO, FSQ-201) containing a mixture of oligo(dT) and random primers. The cDNA was used for qPCR with the 2× SG Fast qPCR Master Mix kit (Sangon Biotech, B639272) in duplicate wells on a QuantStudio 7 Flex Real-Time PCR System (Tongji University Advanced Institute of Translational Medicine) with the following cycle conditions: activation at 95 °C for 10 min; 40 cycles of denaturation at 95 °C for 15 s and annealing/extension at 60 °C for 60 s; and a melting curve for assessment of amplicons. For tumor tissues, tumor pieces were mechanically disrupted with surgical scissors in PBS, then homogenized using a tissue grinder, and RNA was extracted as described above. All primer sequences used in this study are provided in Supplementary information, Materials and Methods.

### Flow cytometry analysis in vitro

To assess the effect of macrophage polarization or changes in MHC I expression on LLC cells induced by MagLMP, RAW 264.7 or LLC cells were seeded as described previously. Cells were incubated with 20 μg/mL MNMs and stimulated by 1 Hz RMF for 15 min. All groups were washed with PBS three times and then collected into 1.5-mL centrifuge tubes. Blocked Purified Rat Anti-Mouse CD16/CD32 (BD Biosciences, 553141) was used at a 1:400 dilution in flow buffer (2.5% FBS mixed into 97.5% PBS) to block non-specific sites on cells for 20 min at 4 °C. The reaction was terminated with 1 mL flow buffer, and cells were collected after centrifugation at 1400 rpm for 4 min. Cells were incubated with BV421 Rat Anti-Mouse CD86 (1:100, BD Biosciences, 565388) and Alexa Fluor 647 Rat Anti-Mouse CD206 (1:100, BD Biosciences, 565250) for 30 min to label M1 and M2 subtypes. LLC cells were incubated with PE Rat Anti-Mouse MHC I (1:100, BioLegend, 116507) for 30 min. After the reaction was terminated and the cells were centrifuged, flow buffer was used to dilute the cells, and flow cytometry was performed.

### Enzyme-linked immunosorbent assay (ELISA)

All macrophages (RAW 264.7 and BMDMs) were seeded and polarized into M1 or M2 subtypes with the corresponding inducing factors as described previously. M2 macrophages were incubated with 20 μg/mL MNMs and stimulated by 1 Hz RMF for 15 min. After culturing for 24 h, the supernatants of the different groups were collected, and the contents of IL-1β, IL-4, IL-6, and IL-10 were detected using corresponding ELISA kits (Wuhan Biofavor Biotech Service, China).

### Western blot analysis

All macrophages (RAW 264.7 and THP-1 wild-type cells or *Gal9*-KO cells) were seeded and induced to obtain M1 and M2 subtypes. After incubation with 20 μg/mL MNMs, M2 macrophages were stimulated by 1 Hz RMF for 15 min. After treatment, the M2 and M2-RMF groups were cultured for 6 h and 12 h. Compound C (10 μM; Selleck, 866405-64-3) or 1 mM metformin (Selleck, 657-24-9) was also used to stimulate the related groups for 1 h. All cells were collected using sodium dodecyl sulfate (SDS) loading buffer, lysed with RIPA buffer, separated by SDS polyacrylamide gel electrophoresis (SDS-PAGE), and transferred to PVDF membranes. After blocking with 5% milk, the membranes were incubated with the related antibodies overnight at 4 °C. The mixture was purified, and horseradish peroxidase-linked rabbit anti-goat secondary antibody was added. The signal was detected using a gel and blot imaging system, and band densities were analyzed using ImageJ.

For co-immunoprecipitation, cells from different groups were lysed after stimulation, and whole-cell lysate extracts were incubated with 3 μg AMPK (CST, 5832 T) antibody or IgG control overnight at 4 °C. Extracts were then incubated with protein A/G Sepharose beads for 2 h at 4 °C, washed five times with cell lysis buffer, and eluted in 2× loading buffer at 95 °C for 10 min. Eluates and cell lysate extracts (input samples) were resolved by SDS-PAGE, transferred to PVDF membranes (Millipore, ISEQ00010), and immunoblotted.

### Transwell migration assay

To observe the migration of tumor cells after RMF stimulation, a Transwell assay was performed. LLC cells were seeded in the upper compartment of the Transwell (8.0-µm pore polycarbonate membrane, Costar, 3428) at a density of 1 × 10^4^ cells per chamber, and 20 μg/mL MNMs were added and co-cultured for 24 h. After 1 Hz RMF stimulation for 15 min and culture for 24 h, the cells were fixed in 4% PFA. The upper layer of LLC cells was removed using a cotton swab, and cells in the lower layer were stained with 0.1% crystal violet for 20 min. After three washes with PBS, 33% glacial acetic acid was used to elute the crystal violet for 10 min. The absorbance of the solution was measured at 570 nm using a microtiter plate reader (ELx808, BioTek).

To examine the ability of MNMs to penetrate the endothelial barrier, a Transwell-based endothelial penetration assay was performed. Endothelial cells were seeded in the upper chamber at a density of 1 × 10^5^ cells per well to form a confluent monolayer. Formation of a tight monolayer was confirmed by measuring transendothelial electrical resistance (TEER) using a voltohmmeter (Millicell ERS-2, Merck Millipore), and experiments were initiated when TEER values reached approximately 200 Ω·cm^2^. Fifty micrograms of MNMs were added to the upper chamber, with or without placing a magnet beneath the culture plate to provide magnetic guidance. At 30 min, 1 h, 2 h, and 4 h, the medium from the lower chamber was collected, and the Fe content was quantified by ICP-MS.

### Seahorse extracellular flux analysis

Respiration and glycolytic rates were measured in RAW 264.7 cells using a 24-well Seahorse XF24 Extracellular Flux Analyzer. Macrophages in Control, MNM, and RMF groups were plated in XF24 microplates (2 × 10^5^ cells per well) and incubated overnight.

For mitochondrial respiration measurement, the Agilent XF Cell Mito Stress Test Kit (#103015) was used. Before the assay, cells were incubated with Seahorse XF DMEM containing 10 mM glucose, 5 mM sodium pyruvate, and 2 mM glutamine (pH 7.4) in a CO_2_-free incubator for 1 h. The oxygen consumption rate (OCR) was measured at baseline and after sequential addition of oligomycin (1 μM, ATP synthase inhibitor), carbonyl cyanide-4-(trifluoromethoxy) phenylhydrazone (FCCP, 1.6 μM, uncoupler of oxidative phosphorylation), and a combination of rotenone (0.5 μM, complex I inhibitor) and antimycin A (0.5 μM, complex III inhibitor).

For measurement of glycolytic function, the Agilent XF Glycolysis Stress Test Kit (#103020) was used. Cells were incubated with Seahorse XF Base Medium supplemented with 2 mM glutamine (pH 7.4) in a CO_2_-free incubator for 1 h. Extracellular acidification rate (ECAR) was measured at baseline and after sequential addition of glucose (10 mM), oligomycin (1 μM), and 2-deoxy-D-glucose (2-DG, 50 mM, a glycolysis inhibitor).

Each condition was performed in three biological replicates. After the assay, cells were fixed in 4% paraformaldehyde (PFA), and OCR/ECAR values were normalized to cell numbers (quantified by Hoechst staining) and to the baseline value at the initial time point.

### RNA sequencing

RNA was extracted and purified from all groups following the standard procedures described for RT-qPCR, and the purity and concentration of total RNA were measured using the NanoDrop 2000 system. For RNA sequencing, mRNA was purified by polyT selection. PCR was used to enrich the template and obtain the cDNA library. A Qubit fluorometer was used to measure the concentration of the cDNA library, and qPCR was then performed to obtain accurate quantification. The samples were sequenced on the Illumina NovaSeq 6000 system at Berry Genomics Corporation to obtain 150-bp paired-end reads. Differentially expressed genes (DEGs) between the M2-RMF and M2 groups were identified using DESeq2.^62^ GSEA was performed using clusterProfiler, and the results were plotted with enrichplot.^63^ The *P* values were corrected using the Benjamini–Hochberg approach, and genes with a *P* value < 0.05 were considered significant.

### Quantification of metabolites by liquid chromatography-mass spectrometry (LC-MS)

RAW 264.7 cells were washed with PBS and lysed with 200 μL pre-cooled extraction buffer (40:40:20 methanol: acetonitrile: water). Cells were then scraped from plates using cell scrapers and transferred into Eppendorf tubes. After 30 s of vortexing, the samples were centrifuged at 16,000× *g* for 20 min at 4 °C. The supernatants were transferred to new 1.5-mL tubes and centrifuged at 16,000× *g* for 30 min at 4 °C, and the resulting supernatants were transferred to LC-MS vials for analysis.

LC separation was performed on a Vanquish UHPLC system (Thermo Fisher Scientific) with a HILIC column (2.1 × 150 mm, 5 μm, HILICON) and a column temperature of 25 °C. Solvent A was 95% water and 5% acetonitrile with 20 mM ammonium acetate and 20 mM ammonium hydroxide (pH 9.4), and solvent B was acetonitrile. The gradient was 0–2 min, 95% B; 3–7 min, 75% B; 8–9 min, 70% B; 10–12 min, 50% B; 13–14 min, 25% B; 16–20.5 min, 0% B; and 21–28 min, 90% B, with an injection volume of 5 μL. The Q Exactive Plus mass spectrometer was operated in switching negative/positive ion mode, scanning m/z 60–900 with a resolution of 140,000 at m/z 200 (AGC target 3e6, maximum IT 200 ms). Raw data were converted to mzXML format using MSconvert and processed using EI-MAVEN for peak detection, extraction, alignment, and integration.

### scRNA-seq

scRNA-seq of tumor tissues was performed at Berry Genomics Corporation. CellRanger (Version 7.0.0) was used to align scRNA-seq reads to the mm10 reference genome and to filter and count barcodes and UMIs. Data normalization, cell clustering, and dimensional reduction were performed using the Python package Scanpy (Version 1.7.2).^64^

For quality control, genes expressed in fewer than three cells were excluded from the merged cell-gene count matrix. Cell filtering thresholds were determined on the basis of data distribution and expression patterns. Cells that expressed fewer than 200 genes and cells with greater than 10% mitochondrial gene counts were excluded to ensure cell quality. Cells that expressed more than 8000 genes were excluded to avoid potential doublets. After clustering, cell clusters that overexpressed markers of more than two cell types were also removed from the analysis.

The resulting matrix included 94,218 cells and 25,964 genes. Data were normalized using the Scanpy function pp.normalize_per_cell, and pp.log1p was used for log transformation. Highly variable genes were identified using the pp.highly_variable_genes function with default parameters, followed by data scaling with the pp.scale function. Principal component analysis was carried out using the tl.pca function for linear dimensionality reduction on highly variable genes. A neighborhood graph was computed with pp.neighbors using the top 40 PCs, and Uniform Manifold Approximation and Projection (UMAP) was performed using the tl.umap function. Leiden clustering was performed using the tl.leiden function with a resolution of 0.4. The resulting clusters were annotated on the basis of their expression of canonical markers. Macrophages, T cells, and neutrophils were extracted separately from the integrated single-cell dataset on the basis of initial cluster annotations. For each cell type, a second round of dimensionality reduction and clustering was performed. Subtype annotations were based on the expression of canonical marker genes.

Differential expression analysis of macrophage cells was performed using the GSEA Java tool.^65^ The log_2_ mean expression fold-change between treatment and control was calculated for each gene, and the log_2_ fold change rank data were used as input for GSEA preranked analysis. The hallmark gene set (mh.all.v2023.2.Mm.symbols.gmt) was used as the analysis database.

### Subcutaneous tumor implantation and antitumor therapy in vivo

Two implantation methods for mouse tumor models were used to assess whether MagLMP could inhibit tumor growth in vivo. First, 1 × 10^6^ LLC cells were directly implanted subcutaneously into the thighs of male C57BL/6 mice. Tumor volume was calculated as V = (π × L × W^2^)/6, where L is the maximum tumor diameter, and W is the minimum tumor diameter. When the tumor volume of LLC-tumor-bearing mice reached ~50 mm^3^, MagLMP antitumor therapy was performed for 14 days. Tumor volumes and body weights of all mice were measured every day. For macrophage depletion in tumor tissues, mice were treated with CL using two dosing regimens: (1) intraperitoneal injection of 200 μL (5 mg/mL, YEASEN) once daily for three consecutive days before RMF treatment, and (2) intravenous injection of 200 μL on day 1, followed by 100 μL every three days during the treatment period until completion. To ensure consistency in CL administration, except for the validation experiments comparing the efficacy of the two dosing regimens (Supplementary information, Fig. S9a–d), all other experiments used the pre-treatment intraperitoneal injection protocol.

After 14 days of observation, mice were deeply anesthetized by intraperitoneal injection of 1% sodium pentobarbital and then cardiac-perfused with 10 mL PBS followed by 10 mL 4% PFA. After tumor tissues from all groups were photographed, all tissues were stored in 4% PFA for further experiments. Four groups of mouse tumor models were established through direct implantation of LLC cells.

Group 1: observing the antitumor effect of MagLMP therapy with or without CL treatment in a subcutaneous tumor model. When the tumor volume reached the requirements described above, MNMs were injected into tumor tissues at a dosage of 50 μg MNMs/25 μL PBS per mouse for 4 consecutive days. For the control group, PBS (25 μL) was intratumorally injected for 4 consecutive days. For the RMF group, tumors were stimulated with 1 Hz RMF for 30 min on 14 consecutive days, beginning on the second day after MNM injection. For the CL/RMF group, CL was injected intraperitoneally at 200 µL once daily for three consecutive days, followed by MNM injection and RMF stimulation as described above.

To further assess the role of macrophages in MagLMP-mediated antitumor effects, BMDMs were isolated from wild-type mice and differentiated by M-CSF treatment (20 ng/mL) in vitro. Mature BMDMs were then incubated with 100 μg/mL MNMs for 24 h. The MNM-loaded BMDMs were adoptively transferred (10^6^ cells per mouse) via intratumoral injection into subcutaneous tumors of macrophage-depleted mice that had been pretreated with CL (200 µL injected intraperitoneally once daily for three consecutive days). Tumors were then treated with or without RMF stimulation (1 Hz, 30 min) for 14 consecutive days.

To evaluate the effects of MagLMP on LLC cells in vivo, LLC cells were co-cultured with MNMs (100 μg per 1 × 10^6^ cells) for 24 h. The MNM-loaded LLC cells were then directly implanted subcutaneously into the thighs of male C57BL/6 mice, followed by daily RMF stimulation (1 Hz, 30 min) for 14 consecutive days.

Group 2: observing the antitumor effect of MagLMP therapy in 4T1 and B16 subcutaneous tumor models. 4T1 and B16 cells (1 × 10^6^) were directly implanted subcutaneously in the thighs of male C57BL/6 mice. When the tumor volume reached the requirements described above, MNMs were injected into tumor tissues at a dosage of 50 μg MNMs/25 μL PBS per mouse for 4 consecutive days. For the control group, PBS (25 μL) was intratumorally injected for 4 consecutive days. For the RMF group, the tumors were stimulated with 1 Hz RMF for 30 min on 14 consecutive days, beginning on the second day after PBS (RMF (MNM–) group) or MNM (RMF (MNM + ) group) injection.

Group 3: observing the antitumor effect of MagLMP therapy by tail vein injection of MNMs in a subcutaneous tumor model. For the MNM group, MNMs were injected through the tail vein (100 μg MNMs/25 μL PBS per mouse) once a day for 4 times. For the control group, 100 μL PBS was injected through the tail vein 4 times. For the RMF group, RMF stimulation was performed for 14 consecutive days beginning on the second day. For the RMF^MF Guide^ group, a magnet was fixed to the tumor site within 4 days of MNM injection to guide MNM accumulation in the tumor tissues, and RMF stimulation was performed as described above.

Group 4: observing the antitumor effect of MagLMP by tail vein injection of MNMs in AIS of the lung in a mouse model. After being deeply anesthetized, the mice were fixed in the supine position on the operating table, and the anterior chest wall was disinfected with 75% alcohol. Next, a small incision of ~5 mm was made at ~1.5 cm above the costal arch of the left anterior axillary line. The skin and subcutaneous tissue were separated, and the chest wall was exposed. A total of 100 μL of LLC cells and Matrigel suspension (10^6^ LLC cells dispersed in 50 μL PBS and mixed with 50 μL Matrigel) were injected into the left lung. The injection depth was ~3 mm, and the incisions were closed sequentially. Two batches of in situ lung cancer mouse models were generated in parallel. In one batch, three mice were sacrificed on days 6, 9, 12, 15, and 20 according to the prescribed procedure, and lung tissues were removed to visualize the tumor volumes. Another batch of mice was randomly divided into 4 groups with 10 mice in each group. The PBS, MNM, RMF, and RMF^MF Guide^ groups received the treatments described above. After MagLMP therapy for 14 days, 1 mouse was randomly chosen for the removal of lung tissue as described above, and the tissue was fixed in 4% PFA for subsequent use. The remaining nine mice in each group were observed for 120 days to assess overall survival.

Second, a mouse-derived allograft (MDA) model was used to maintain the tumor microenvironment and tumor heterogeneity. First, 1 × 10^6^ LLC cells were implanted subcutaneously in the right thighs of several male C57BL/6 mice, which were used to produce additional tumor tissue for transplant. After the tumor volume reached 100–500 mm^3^, the tumor tissue was removed, and a tissue block of ~1 mm in diameter was cut. The small pieces of tissue were replanted subcutaneously in the thighs of C57BL/6 mice with a trocar. When the tumor volume of LLC-tumor-bearing mice reached approximately 50 mm^3^, MagLMP antitumor therapy was performed for 14 days. Tumors were observed and data recorded after MagLMP treatment as described above. Four groups of mouse tumor models were established on the basis of the MDA mouse model.

Group 1: verifying the antitumor effect of MagLMP combined with anti-PD-1 antibody (BioXcell, BE0146) in a subcutaneous tumor model. The subcutaneous MDA mouse model was created first. Then, 100 μg MNMs/25 μL PBS were injected through the tail vein once daily for 4 consecutive days, with 100 μL PBS injected similarly in the control group. For the RMF group, the tumors were stimulated with 1 Hz RMF for 30 min on 14 consecutive days, beginning on the second day after MNM injection. For the iPD-1 and iPD-1/RMF groups, anti-PD-1 antibody was injected intraperitoneally every two days at a dosage of 100 μg PD-1 inhibitor/100 μL PBS per mouse on the second day after MNM injection for 14 days, accompanied by or without MagLMP treatment for 14 days.

Group 2: verifying the effect of AMPK on the MagLMP strategy for antitumor immunity using an AMPK inhibitor. The subcutaneous MDA mouse model was created, and MNMs were then injected into tumor tissues at a dosage of 50 μg MNMs/25 μL PBS per mouse for 4 consecutive days when tumor volumes reached approximately 50 mm^3^. The tumors were stimulated with 1 Hz RMF for 30 min on 14 consecutive days, beginning 24 h after MNM injection. For the treatment group that received MagLMP combined with the AMPK inhibitor, the mice were intraperitoneally injected with an AMPK inhibitor (Compound C, 20 mg/kg per mouse) every two days.

Group 3: verifying the effect of Gal9 on the MagLMP strategy for antitumor immunity using *Gal9*-KO BMDMs. *Gal9* gene knockout was performed on C57BL/6 wild-type mice. BMDMs were isolated from both C57BL/6 wild-type and *Gal9*-KO mice and differentiated using M-CSF (20 ng/mL). Mature BMDMs were incubated with 100 μg/mL MNMs for 24 h. The MNM-loaded BMDMs were then adoptively transferred into subcutaneous tumors (10^6^ cells per mouse) of macrophage-depleted mice (pretreated with CL) via intratumoral injection. Tumors that received wild-type BMDMs were subsequently stimulated with RMF (1 Hz, 30 min) daily for 14 consecutive days.

Group 4: verifying the dependence of the MagLMP strategy for antitumor immunity on Gal9 and AMPK using *Gal9*-KO BMDMs. BMDMs were isolated from *Gal9*-KO mice and differentiated using M-CSF (20 ng/mL). Lentiviral particles encoding AMPK-targeting shRNA were used to transduce BMDMs for AMPK knockdown, and particles carrying negative control (NC) shRNA served as the control. Mature BMDMs were incubated with 100 μg/mL MNMs for 24 h. The MNM-loaded BMDMs were subsequently adoptively transferred into subcutaneous tumors (10^6^ cells per mouse) of macrophage-depleted mice (pretreated with CL) via intratumoral injection. Tumors that received these BMDMs were stimulated with RMF (1 Hz, 30 min) daily for 14 consecutive days.

### Flow cytometry analysis of in vivo tissues

After completing all treatments, mice were sacrificed by neck amputation after being deeply anesthetized by intraperitoneal injection with 1% sodium pentobarbital, and the entire tumor tissue was quickly extracted. Surgical scissors were used to cut up the tumor tissue as much as possible, and the cut tissue was placed in a mixed enzyme solution (0.05 mg/mL type I collagenase, 0.05 mg/mL type IV collagenase, 0.025 mg/mL hyaluronidase, 0.01 mg/mL deoxyribonuclease, and 0.05 mg/mL trypsin inhibitor). The mixed solution was placed in a shaker at 37 °C for 15 min. After the undigested solid tissue blocks were discarded, red blood cell lysis buffer (Beyotime Biotech, C3702) was added for 5 min, and lysis was terminated with flow buffer (2.5% FBS in 97.5% PBS). Cell blocking and staining were performed in vitro as described above. PE Rat Anti-Mouse F4/80 (BD Biosciences, 565410), PerCP-Cy5.5 Rat Anti-CD11b (BD Biosciences, 550993), BV421 Rat Anti-Mouse CD86, and Alexa Fluor 647 Rat Anti-Mouse CD206 were used to label different macrophage subtypes in order to analyze the ratio of M1 and M2 macrophages in the tumor tissue. Finally, the cells were resuspended in 300 μL flow buffer and passed through 40-μm filters.

Macrophage subtypes in the spleen were also analyzed by flow cytometry. Mouse spleens were completely removed. After mechanical trituration, cells were filtered through 40-μm filters. Red blood cell lysis buffer was added for 5 min, and the reaction was terminated with flow buffer. Cells were blocked and stained separately as described above. The data were analyzed using CytExpert Software (Version 2.4.0.28).

To analyze the ratio of different cell types that internalized MNMs in tumor tissues, LLC cells were implanted subcutaneously in the thighs of C57BL/6 mice. Once the tumors reached the appropriate size, MNMs were directly injected into the tumor once daily, followed by RMF stimulation for 14 consecutive days. In the intravenous injection model, mice were administered MNMs (100 μg per mouse) via tail vein injection. An external magnet was applied to guide the enrichment of MNMs at the tumor site, followed by RMF stimulation for 14 consecutive days. At the end of the treatment period, tumors were excised and enzymatically digested as described previously. After red blood cell lysis, all cells were magnetically separated for 20 min. All the fluid in the tubes was removed, and the remaining cells adsorbed on the tube walls by the magnet were those with internalized MNMs. Fc receptors were blocked with anti-CD16/32 antibody. BV-570 FVS was used to distinguish living cells. APC-Cy7 CD45 (BD Biosciences, 564406) was used to differentiate monocytes and tumor cells. Macrophages were screened by BV785-F4/80 and BV605-CD11b. M1-like macrophages were further distinguished by CD80 expression. Neutrophils were labeled with PE-Cy7 and PerCP-Cy5.5 CD11b. DCs were identified using BB700-conjugated CD11c and BV421-conjugated MHC-II antibodies.

To evaluate the depletion efficiency of CL on tumor-associated macrophages, mice were pretreated with CL intraperitoneally once daily for three consecutive days. To assess the in vivo persistence of intratumorally transferred BMDMs, cells were labeled with DiD dye (1:1000, Beyotime Biotech, C1039) for 20 min prior to injection into the tumor tissues. In both experiments, tumors were collected on days 1, 7, and 14 after treatment and processed as described above, and endogenous macrophages were stained for analysis. DiD labeling was used to track the adoptively transferred BMDMs.

To detect the activation of tumor-specific CD8^+^ T cells in the spleen, LLC cells with OVA-LLC were constructed and implanted subcutaneously into C57BL/6 mice. MNMs were injected into the tumor directly before MagLMP was performed on these mice. Mice were treated with or without CL. After tumors were treated with programmable MagLMP for 14 days, the spleen of each mouse was dissected and prepared into a single-cell suspension. These cells were co-cultured with OVA-LLC cells for 6 h in the presence of APC-Cy7 CD107a (BioLegend, 121616) and brefeldin A (MCE, 20350-15-6). BV650 CD45 (BioLegend, 103151) was used to differentiate monocytes. PerCP-Cy5.5 CD3 (BD Biosciences, 551163) and FITC CD8 (MBL, 553035) were used to label CD8^+^ T cells. PE OVA tetramer (MBL, TS-5001-1C), BV421 granzyme B (BioLegend, 396414), and APC IFN-γ (BD Biosciences, 554413) were used to identify the activation of tumor-specific CD8^+^ T cells.

To evaluate changes in MHC I expression on tumor cells after MagLMP treatment, GFP-labeled LLC cells were implanted subcutaneously into C57BL/6 mice. MagLMP treatment was performed for 14 consecutive days as described above. Tumors were harvested on days 1, 7, and 14 post-treatment for flow cytometric analysis. Tumor tissues were dissociated into single-cell suspensions, and PE-conjugated anti-MHC I antibody was used to detect MHC I expression on GFP^+^ LLC tumor cells.

All the processed cells were resuspended in 300 μL flow buffer and analyzed by flow cytometry. Data were processed using CytExpert software.

### Flow cytometric sorting of tumor-associated macrophages

LLC cells were implanted subcutaneously into C57BL/6 mice. MNMs were administered intratumorally as described above, followed by 14 days of MagLMP treatment. For the intravenous injection model, MNMs were injected via the tail vein and guided to accumulate in the tumor tissue using an external magnet. Tumor tissues were then dissociated into single-cell suspensions. Fc receptors were blocked with an anti-CD16/32 antibody, and BV-570 FVS was used to exclude dead cells. Tumor-associated macrophages were sorted and collected using APC-Cy7 CD45, BV785-F4/80, and BV605-CD11b markers. Subsequently, Fe levels in sorted macrophages were quantified by ICP-MS. Total RNA was extracted according to standard protocols for RT-qPCR analysis of macrophage-related genes. In parallel, proteins were extracted to assess AMPK, p-AMPK, and iNOS expression by western blotting.

In the animal experiment involving *Gal9*-KO BMDMs transduced with AMPK-targeting shRNA and adoptively transferred into tumors, tumor tissues were dissociated into single-cell suspensions after 7 days of MagLMP treatment. The transferred BMDMs were sorted on the basis of GFP positivity (as the AMPK-targeting shRNA construct carried a GFP reporter) by flow cytometry. Proteins were then extracted from the sorted cells to evaluate AMPK expression by western blotting, thus confirming the in vivo knockdown efficiency of AMPK.

### In vivo fluorescence imaging

To evaluate the accumulation of MNMs in tumors in the intravenous injection model, Cy5.5-labeled MNMs (100 μg per mouse) were administered via tail vein injection into subcutaneous tumor-bearing mice. Immediately after injection, a magnet was placed over the tumor site to guide MNM enrichment. Fluorescence intensity at the tumor site was monitored at 30 min, 1 h, 2 h, and 4 h post-injection using an in vivo imaging system (IVIS Lumina XRMS, PerkinElmer).

### Detection of serum inflammatory factors

Mice were intratumorally injected with 50 μg MNMs/25 μL PBS per mouse for 4 consecutive days, treated with MagLMP for 14 days, and sacrificed at different time points. Retro-orbital blood was collected after the mice were deeply anesthetized by intraperitoneal injection with 1% sodium pentobarbital, and the mice were then immediately sacrificed by neck amputation. After standing at room temperature for 2 h, the blood was centrifuged at 15,000 rpm for 20 min at 4 °C. The supernatants from different groups were collected, and the contents of IL-1β and TNF-α were measured using corresponding ELISA kits (Elabscience Biotechnology, China).

### Quantification of Fe in mouse blood and tissues

Mice were intratumorally injected with 50 μg MNMs/25 μL PBS per mouse for 4 consecutive days. After MagLMP therapy for 14 days, heart, liver, spleen, lung, kidney, brain, and tumor tissues were obtained using standard operating procedures after transcardiac perfusion. The isolated tissues were homogenized and lysed in aqua regia, and ICP-MS was performed to quantify the amount of Fe in the different tissues.

To evaluate the biodistribution of MNMs in the intravenous injection model, mice were intravenously administered 100 μg MNMs per mouse with or without magnetic guidance. Blood and tumor tissues were collected at 30 min, 1 h, 2 h, and 4 h after injection under anesthesia. ICP-MS was performed to quantify Fe levels in blood and tumor tissues from the different groups.

### Tissue sectioning, staining, and immunofluorescence

Tumor tissues and organs from different groups were cut into thin slices of approximately 5 mm × 5 mm × 2 mm. To observe cell morphology in tumor tissues and organs, hematoxylin and eosin (H&E) staining (Solarbio, G1120) was performed on the slices. To explore the distribution of MNMs in different organs, sections from the different groups were stained with Prussian blue (Solarbio, G1422). To explore the proportions of different macrophage subtypes in tumor tissues after MagLMP treatment, macrophages in the sections were stained by immunofluorescence with anti-mouse CD206 (R&D Systems, AF2535), anti-mouse CD80 (Proteintech, 14292-1-AP), anti-mouse F4/80 (R&D Systems, MAB5580), and DAPI (Beyotime Biotech, C1002). To explore the infiltration of CD4^+^ and CD8^+^ T cells into tumor tissues, sections were stained with anti-mouse CD4 antibody (Abcam, ab316866), anti-mouse CD8 antibody (Abcam, ab308264), and DAPI. All stained sections were observed by confocal laser scanning microscopy (Leica TCS SP8).

### Quantification and statistical analysis

Statistical details of experiments, including *n* and *P* values, can be found in the figures, figure legends, and methods. Statistical analyses were performed using GraphPad Prism 7.0 (GraphPad Software, CA), and data are presented as means ± SD. Comparisons between two groups were performed using an unpaired two-tailed Student’s *t*-test. Comparisons among multiple groups were performed using one-way ANOVA or two-way ANOVA, followed by Tukey’s honestly significant difference (HSD) post hoc test or Bonferroni’s multiple comparisons post-test, unless otherwise noted. *P* < 0.05 was considered statistically significant. For co-localization experiments, percent co-localization was determined using Manders’ co-localization coefficient (MCC).

## Supplementary information

Supplementary Information, Fig. S1

Supplementary Information, Fig. S2

Supplementary Information, Fig. S3

Supplementary Information, Fig. S4

Supplementary Information, Fig. S5

Supplementary Information, Fig. S6

Supplementary Information, Fig. S7

Supplementary Information, Fig. S8

Supplementary Information, Fig. S9

Supplementary Information, Fig. S10

Supplementary Information, Fig. S11

Supplementary Information, Fig. S12

Supplementary Information, Fig. S13

Supplementary Information, Fig. S14

Supplementary Information, Fig. S15

Supplementary Information, Video S1

Supplementary Information, Video S2

Supplementary Information, Video S3

Supplementary Information, Video S4

Supplementary Information, Video S5

Supplementary Information, Video S6

Supplementary Information, Video S7

Supplementary Information, Video S8

Supplementary Information, Video S9

Supplementary Information, Video S10

Supplementary Information, Video S11

Supplementary Information, Video S12

Supplementary Information, Video S13

Supplementary Information, Video S14

Supplementary Information, Video S15

Supplementary Information, Video legends

Supplementary Information, Materials and Methods

## Data Availability

All data are available in the manuscript or its supplementary materials. Quantitative data for cellular metabolites have been deposited at Mendeley Data (DOI: 10.17632/6h5z3x6rkm.1). RNA sequencing data (GSE273977) and scRNA-seq data (GSE275018) have been deposited in the Gene Expression Omnibus and are publicly available. Any additional information required to reanalyze the data reported in the paper is available from the corresponding authors upon reasonable request.
