# Supplementary material for: Dynamic magneto-mechanical force in lysosomes induces durable macrophage repolarization for antitumor immunity
Source: Cell Res. 2026 Feb 3;36(3):197–218. doi: 10.1038/s41422-025-01217-1 (PMC12909937; doi:10.1038/s41422-025-01217-1)
Supplement: Supplementary file 1 — Supplementary Information, Fig. S1 [file 41422_2025_1217_MOESM1_ESM.pdf]

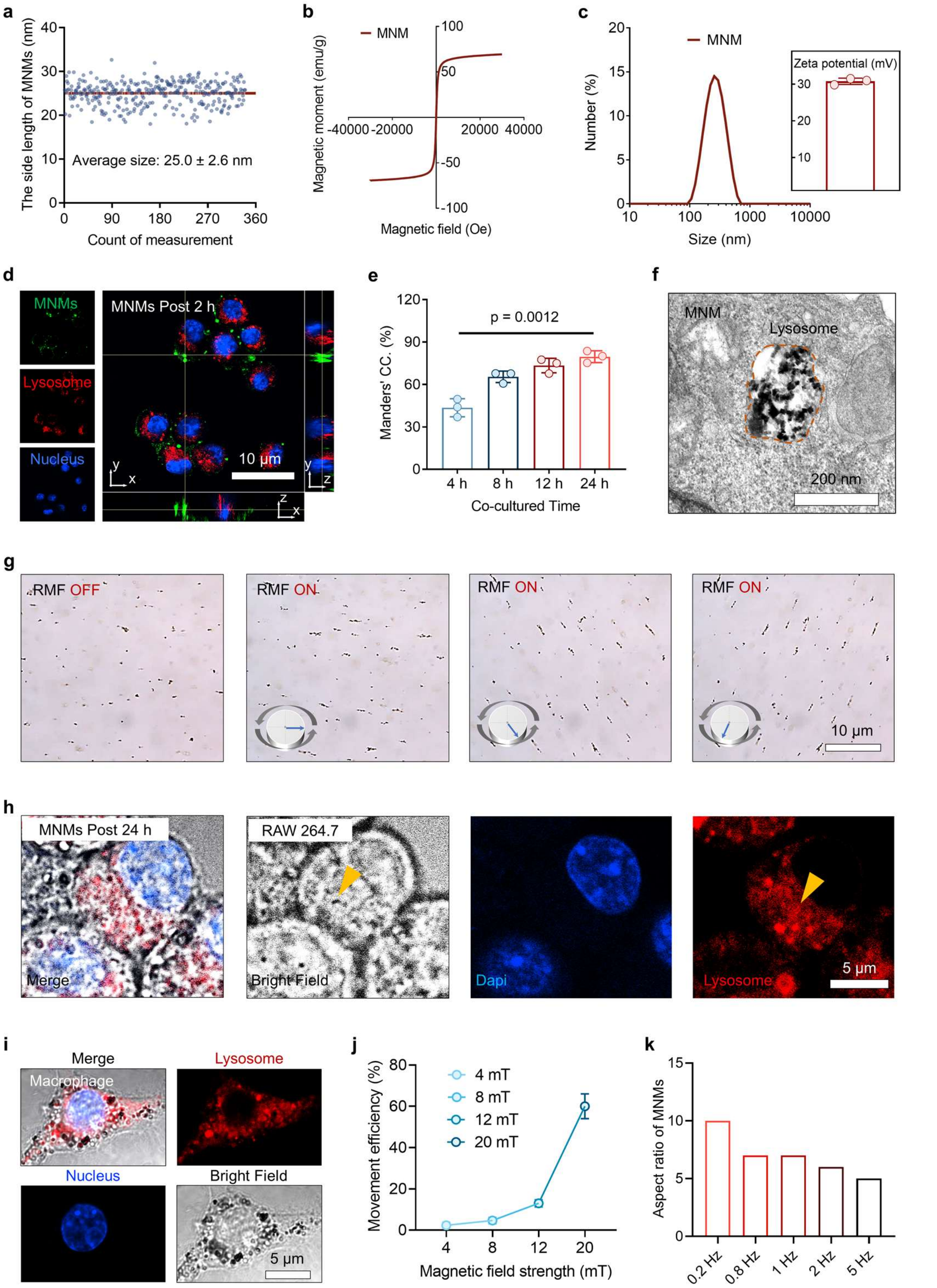

Fig. S1. Characterization of MNMs and the rotational motion of assembled MNMs.

a Statistical analysis of the side length of MNMs (n = 340 independent samples).

b Magnetization vs. magnetic field curves of dry MNMs at 298 K.

c The hydrodynamic size and Zeta potential of MNMs. Data are presented as mean ± s.d. (n = 3 independent samples).

d 3D fluorescence confocal images of RAW 264.7 cells incubated with FITC-labeled MNMs (green) for 2 h. Lysosome was stained with Lysotracker red (red) and nucleus was stained with Hoechst (blue).

e Manders' colocalization coefficient (Manders' CC) and fluorescence images of macrophages incubated with MNMs coated with FITC (green) for 4, 8, 12 or 24 h, respectively. Lysosome was stained with Lysotracker red (red) and nucleus was stained with Hoechst (blue). Data are presented as mean ± s.d. Statistical significance is defined as $p < 0.05$. (n = 3 independent biological replicates).
f Bio-TEM images of MNMs in lysosomes of RAW 264.7 cells.
g Images of self-assembled and rotation of MNMs under 1 Hz RMF stimulation.
h RAW 264.7 cells were incubated with MNMs and then were treated with 1 Hz RMF. Lysosome was stained with Lysotracker red (red) and nucleus was stained with Hoechst (blue). Arrow indicates MNMs assembled within lysosomes. See **Fig. 1d and Supplementary information, Videos S2-S4** for details.
i, j RAW 264.7 cells were incubated with MNMs and then were treated with 1 Hz RMF. Lysosome was stained with Lysotracker red (red) and nucleus was stained with Hoechst (blue). Representative images (**i**) and statistical results (**j**) of movement results under different magnetic strength stimulation (4, 8, 12, 20 mT). See **Supplementary information, Videos 4-7** for details.
k Mathematical model of assembled number of MNMs under different frequencies of RMF.
