# Supplementary material for: Dynamic magneto-mechanical force in lysosomes induces durable macrophage repolarization for antitumor immunity
Source: Cell Res. 2026 Feb 3;36(3):197–218. doi: 10.1038/s41422-025-01217-1 (PMC12909937; doi:10.1038/s41422-025-01217-1)
Supplement: Supplementary file 2 — Supplementary Information, Fig. S2 [file 41422_2025_1217_MOESM2_ESM.pdf]

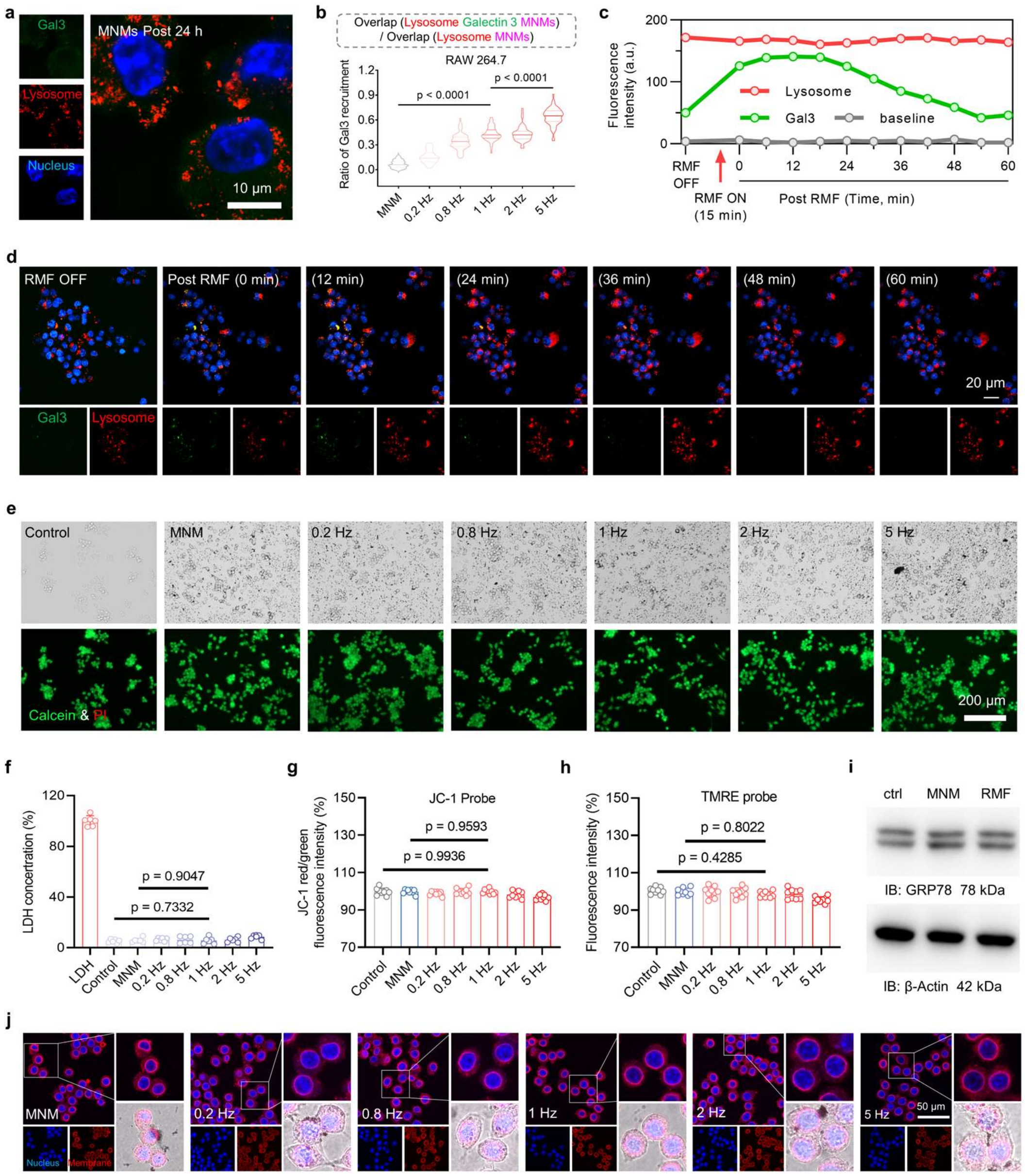

Fig. S2. The effects of RMF stimulation on different organelle membranes.

a-d EGFP-Gal3-transfected RAW 264.7 cells were incubated with MNMs for 24 h. Lysosome was stained with Lysotracker red (red) and nucleus was stained with Hoechst (blue). Representative fluorescence images of these cells without RMF stimulation were shown (**a**). After treated with different RMF frequencies for 15 min, recruitment ratio of Gal3 to lysosome was calculated (**b**). Data are presented as mean ± s.d. Statistical significance is defined as $p < 0.05$ (n = 60 cells from 10 independent biological replicates). Representative continuous fluorescence intensity (**c**) and fluorescence images (**d**) within 1 h of these cells before and after treated by 1 Hz RMF for 15 min were shown.

e-j RAW 264.7 cells were incubated with or without MNMs and then were treated with or without indicated 1 Hz RMF for 15 min. Cells were stained with Calcein (green) and Propidium iodide (PI, red) and representative images were shown (**e**). The concentration of lactic dehydrogenase (LDH) in the supernatant medium of these cells was measured (**f**). Cells were stained with mitochondrial membrane potential fluorescent probe JC-1 or TMRE. Statistical analyses of mitochondrial

membrane potential were performed (**g, h**). Western blotting analysis of GRP78 was examined (**i**). Lamin B1 (red) was stained with the related antibodies, and nucleus was stained with Dapi (blue) and representative images were shown (**j**). Data are presented as mean ± s.d. Statistical significance is defined as $p < 0.05$ (n = 6 independent biological replicates).
