# Supplementary material for: Dynamic magneto-mechanical force in lysosomes induces durable macrophage repolarization for antitumor immunity
Source: Cell Res. 2026 Feb 3;36(3):197–218. doi: 10.1038/s41422-025-01217-1 (PMC12909937; doi:10.1038/s41422-025-01217-1)
Supplement: Supplementary file 3 — Supplementary Information, Fig. S3 [file 41422_2025_1217_MOESM3_ESM.pdf]

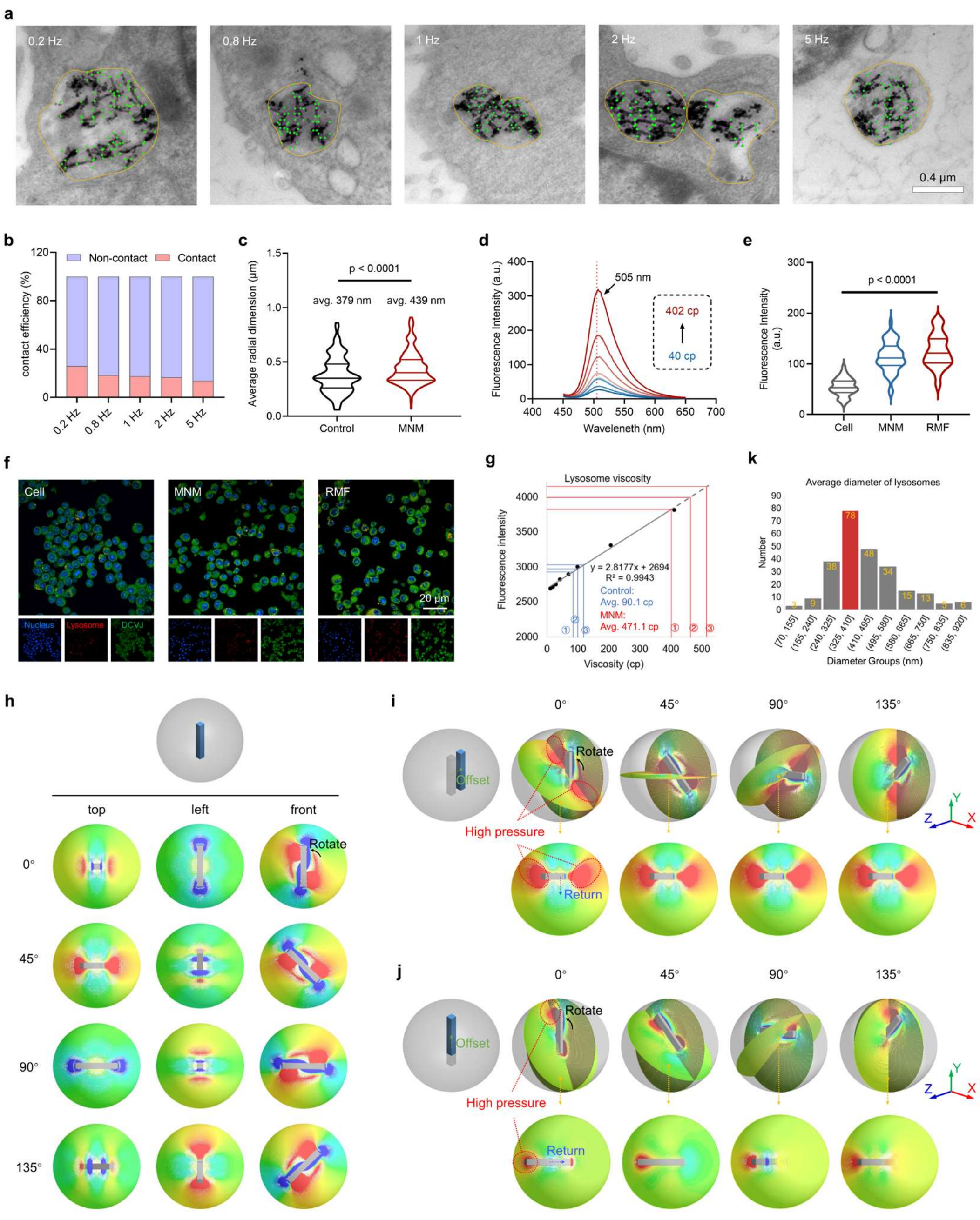

Fig. S3. The location of assembled MNMs in the lysosome during MagLMP treatment.

a, **b** Representative images and statistical results of the membrane contact ratio of assembled MNMs in lysosomes by RMF stimulation for 15 min with different frequencies. Each group collected 20 bio-TEM images using LabelImg software.

c Average lysosome radial dimension statistics of RAW 264.7 cells (Control) and cells incubated with MNMs (MNM). Cells were cultured with FITC-Dextran for 4 hours and then imaged by Z-stack imaging technique.

d Fluorescence intensity of DCVJ (10 μM) with increasing solvent viscosity (from 40 cP to 402 cP, pH 7.0, containing 1% DMSO). Excitation wavelength: 430 nm.

e, f RAW 264.7 cells were incubated with MNMs for 24 h and then treated with 1 Hz RMF for 15 min. Lysosome was stained with Lysotracker red (red) and cell was stained with DCVJ viscosity fluorescence probe (green). Statistical results (**e**) and representative fluorescence images (**f**) of these

cells were shown.

g Lysosome viscosity of Control and MNM groups. The standard curve was prepared with different proportions of glycerol and ddH_2O, and the solution was detected by DCVJ viscosity fluorescence probe. The lysosomes of macrophages were isolated by lysosome extraction kit, and the lysosomal membranes were disrupted by ultrasound at 360 W for 20 min.

h-j FEM simulation on fluidic pressure in a lysosome (with a measured radial dimension of 359.2 nm and 471.1 cp viscosity) when the assembled MNMs' center coincided with the lysosomal center (**h**), deviated from the rotation plane (**i**) or deviated from the lysosomal center, but still within the rotation plane (**j**).

k Average radial dimension of lysosomes in different groups. The ten groups were divided according to the statistical lysosome radial dimension.
