# Supplementary material for: Dynamic magneto-mechanical force in lysosomes induces durable macrophage repolarization for antitumor immunity
Source: Cell Res. 2026 Feb 3;36(3):197–218. doi: 10.1038/s41422-025-01217-1 (PMC12909937; doi:10.1038/s41422-025-01217-1)
Supplement: Supplementary file 4 — Supplementary Information, Fig. S4 [file 41422_2025_1217_MOESM4_ESM.pdf]

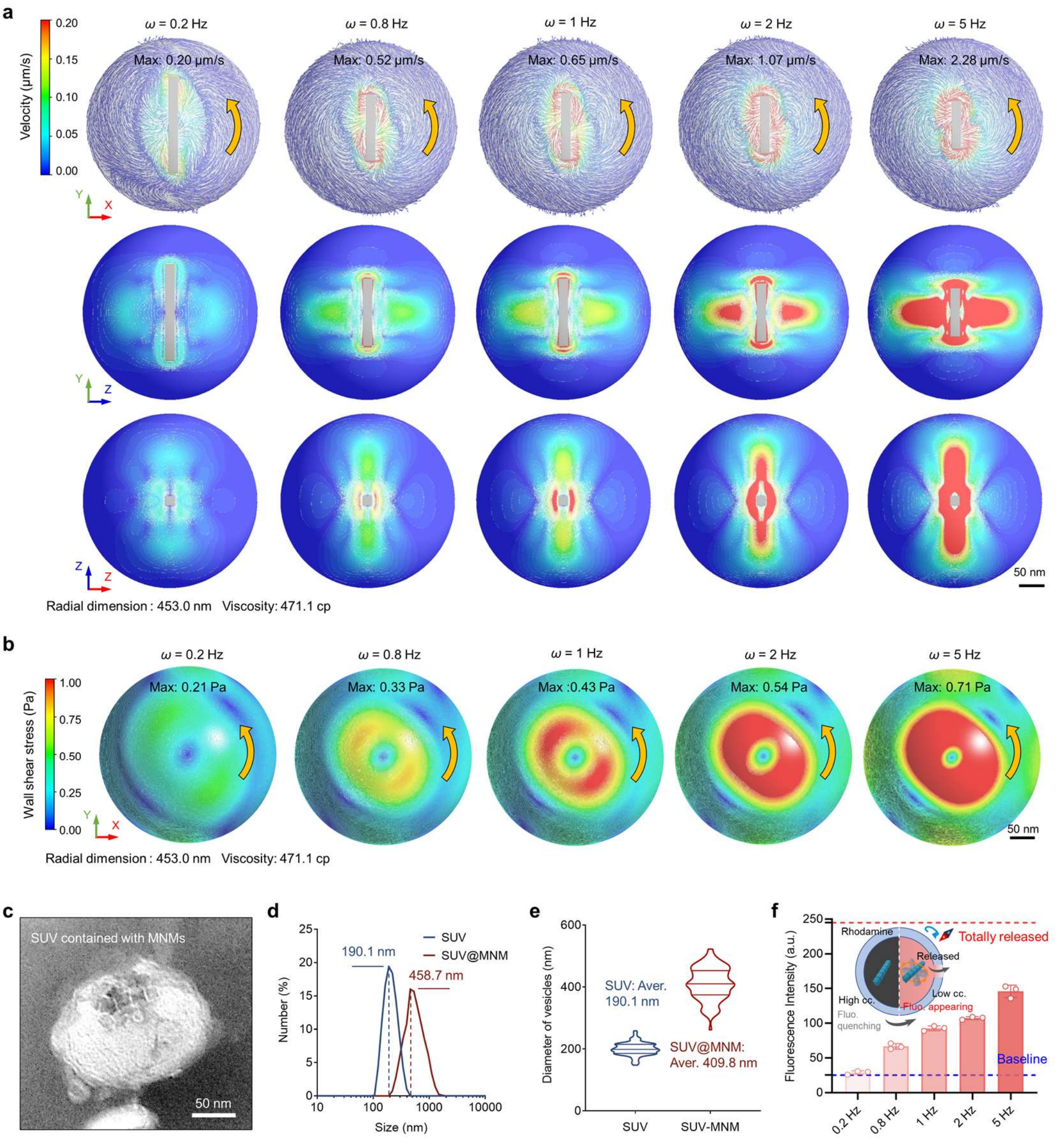

Fig. S4. MagLMP mediates the changes of lysosomal membrane damage.

a, **b** FEM simulation on the maximum velocity (**a**) and maximum wall shear stress (**b**) in a lysosome (with a measured radial dimension of 453.0 nm and 471.1 cp viscosity) under different RMF frequencies for 15 min.

c Morphology of vesicles that enclose MNMs.

d The hydrodynamic size of SUV and SUV@MNM was measured by nanoparticle potentiometer.

e Statistical results for the diameters of SUV and SUV@MNM. Data are presented as mean ± s.d. (n = 100 independent samples).

f Scheme and fluorescence intensity changes of lysosome-like vesicles after RMF stimulation in different frequencies. Data are presented as mean ± s.d. (n = 3 independent biological replicates).
