# Supplementary material for: Dynamic magneto-mechanical force in lysosomes induces durable macrophage repolarization for antitumor immunity
Source: Cell Res. 2026 Feb 3;36(3):197–218. doi: 10.1038/s41422-025-01217-1 (PMC12909937; doi:10.1038/s41422-025-01217-1)
Supplement: Supplementary file 5 — Supplementary Information, Fig. S5 [file 41422_2025_1217_MOESM5_ESM.pdf]

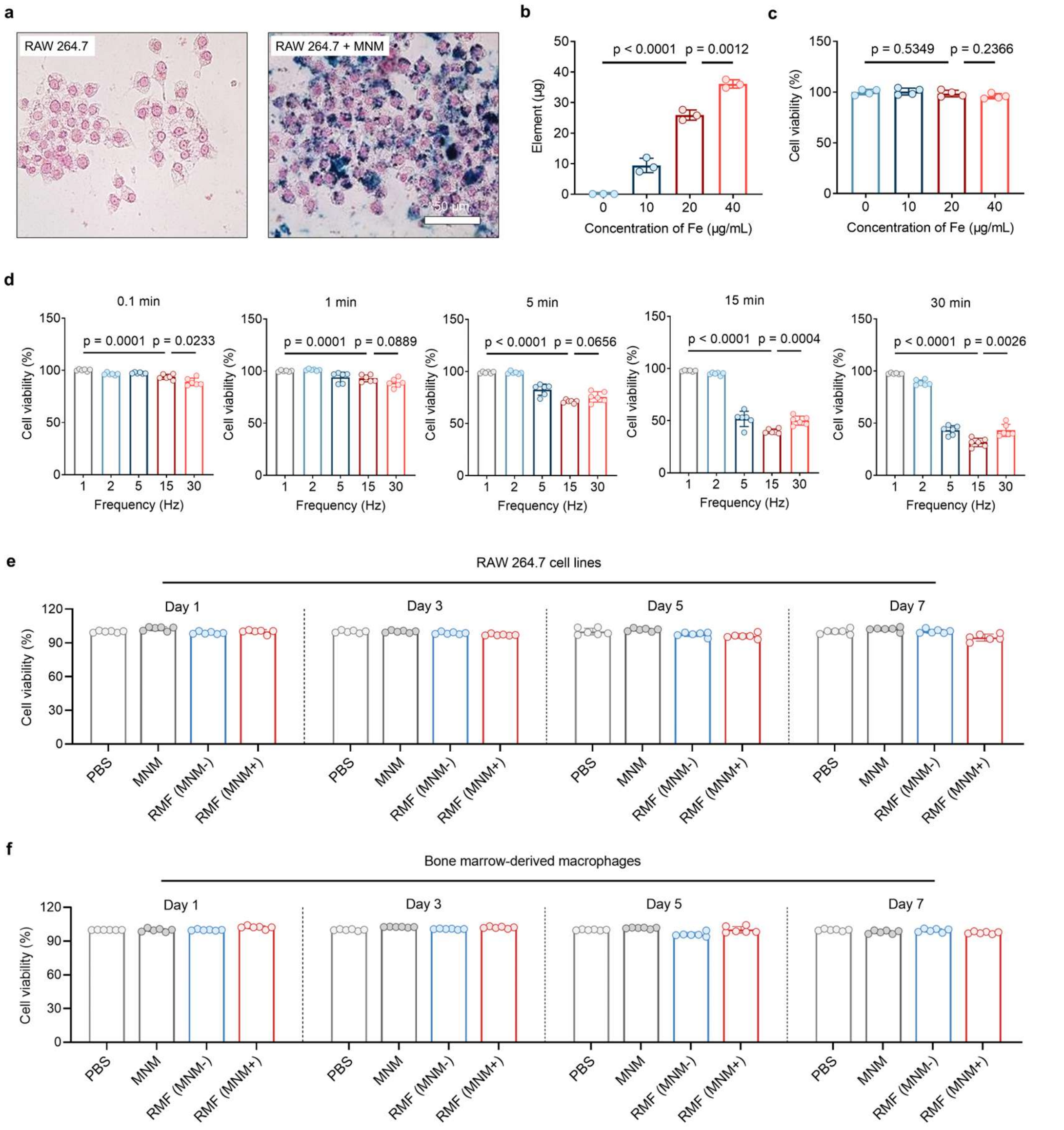

Fig. S5. Endocytosis of MNMs and cell viability of RMF stimulation with different frequencies.

a Representative image of RAW 264.7 cells incubated with or without 20 μg/mL MNMs for 24 h, followed by staining with Prussian blue.

b Content of iron in RAW 264.7 cells incubated with MNMs for 24 h at the iron concentration of 0, 10, 20 and 40 μg/mL (n = 4 independent biological replicates).

c Cell viability of RAW 264.7 cells incubated with MNMs for 24 h at the iron concentration of 0, 10, 20 and 40 μg/mL (n = 4 independent biological replicates).

d RAW 264.7 cells were incubated with 20 μg/mL MNMs for 24 h. Cells were then treated with RMF of 1 Hz, 2 Hz, 5 Hz, 15 Hz or 30 Hz for 0.1 min, 1 min, 5 min, 15 min or 30 min. Cell viability was subsequently measured (n = 6 independent biological replicates).

e, f RAW 264.7 cells (**e**) and bone marrow-derived macrophages (BMDMs) (**f**) were incubated with 20 μg/mL MNMs for 24 h. Cells were then treated with 1 Hz RMF for 15 min per day over 7 days. Cell viability was subsequently measured at day 1, 3, 5 and 7 (n = 6 independent biological replicates).
