# Supplementary material for: Dynamic magneto-mechanical force in lysosomes induces durable macrophage repolarization for antitumor immunity
Source: Cell Res. 2026 Feb 3;36(3):197–218. doi: 10.1038/s41422-025-01217-1 (PMC12909937; doi:10.1038/s41422-025-01217-1)
Supplement: Supplementary file 6 — Supplementary Information, Fig. S6 [file 41422_2025_1217_MOESM6_ESM.pdf]

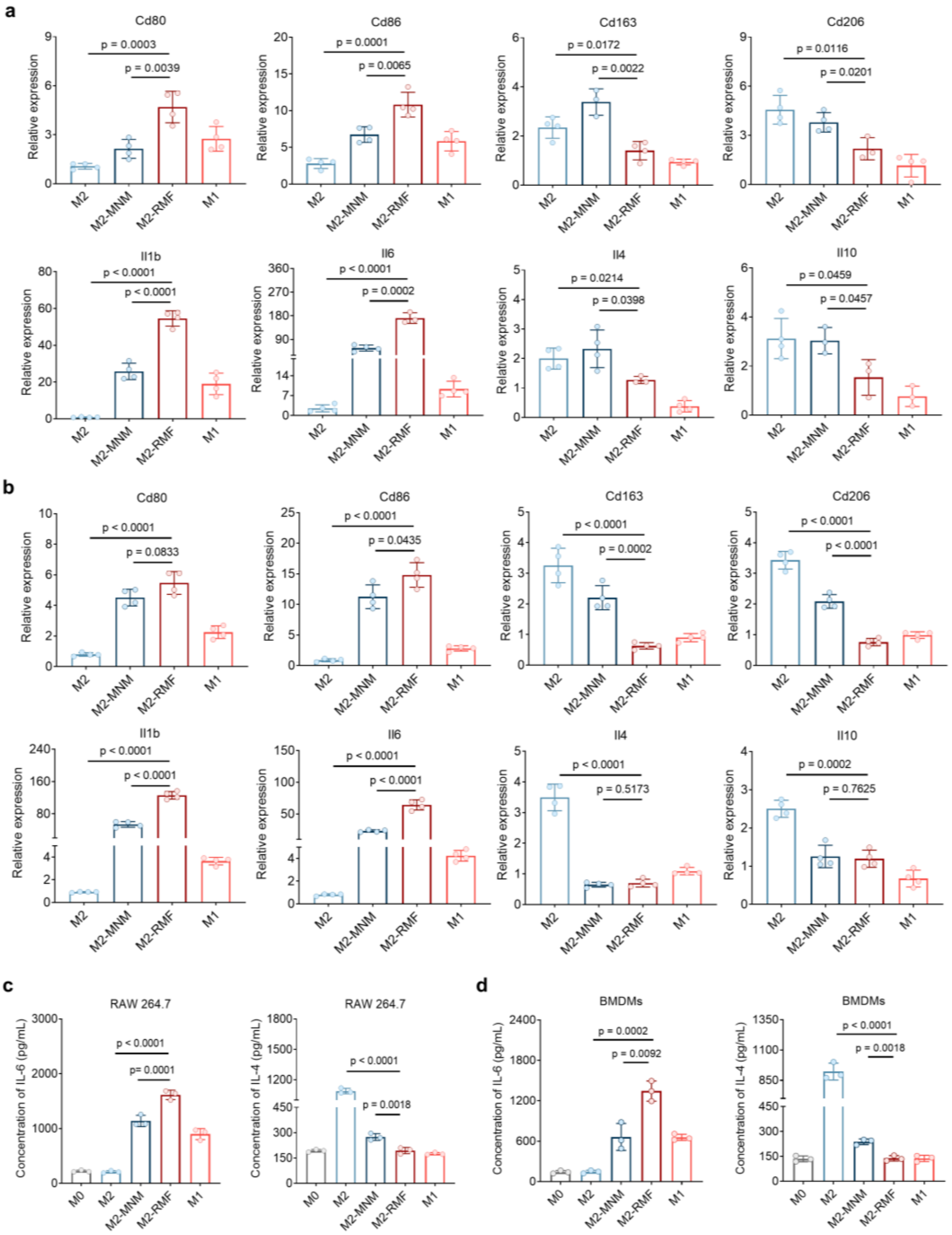

Fig. S6. Effects of the MagLMP strategy on RAW 264.7 and BMDMs macrophage repolarization.

a mRNA level of *Cd80, Cd86, Cd163, Cd206, Il1β, Il4, Il6,* and *Il10* in RAW 264.7-polarized M1 or M2 cells treated with MNM, RMF, both, or neither. (n = 4 independent biological replicates).

b mRNA level of *Cd80, Cd86, Cd163, Cd206, Il1β, Il4, Il6,* and *Il10* in BMDMs-derived M1 or M2 cells treated with MNM, RMF, both, or neither. Data are presented as mean ± s.d. Statistical significance is defined as $p < 0.05$ (n = 4 independent biological replicates).

c, **d** RAW 264.7 cells and BMDMs were differentiated into M0, M1 and M2 macrophages. M2 macrophages were incubated with MNMs and then treated with or without 1 Hz RMF for 15 min. The contents of M1-associated proteins (IL-6) and M2-associated proteins (IL-4) in the conditional medium were examined. Data are presented as mean ± s.d. Statistical significance is defined as $p < 0.05$ (n = 3 independent biological replicates).
