# Supplementary material for: Dynamic magneto-mechanical force in lysosomes induces durable macrophage repolarization for antitumor immunity
Source: Cell Res. 2026 Feb 3;36(3):197–218. doi: 10.1038/s41422-025-01217-1 (PMC12909937; doi:10.1038/s41422-025-01217-1)
Supplement: Supplementary file 7 — Supplementary Information, Fig. S7 [file 41422_2025_1217_MOESM7_ESM.pdf]

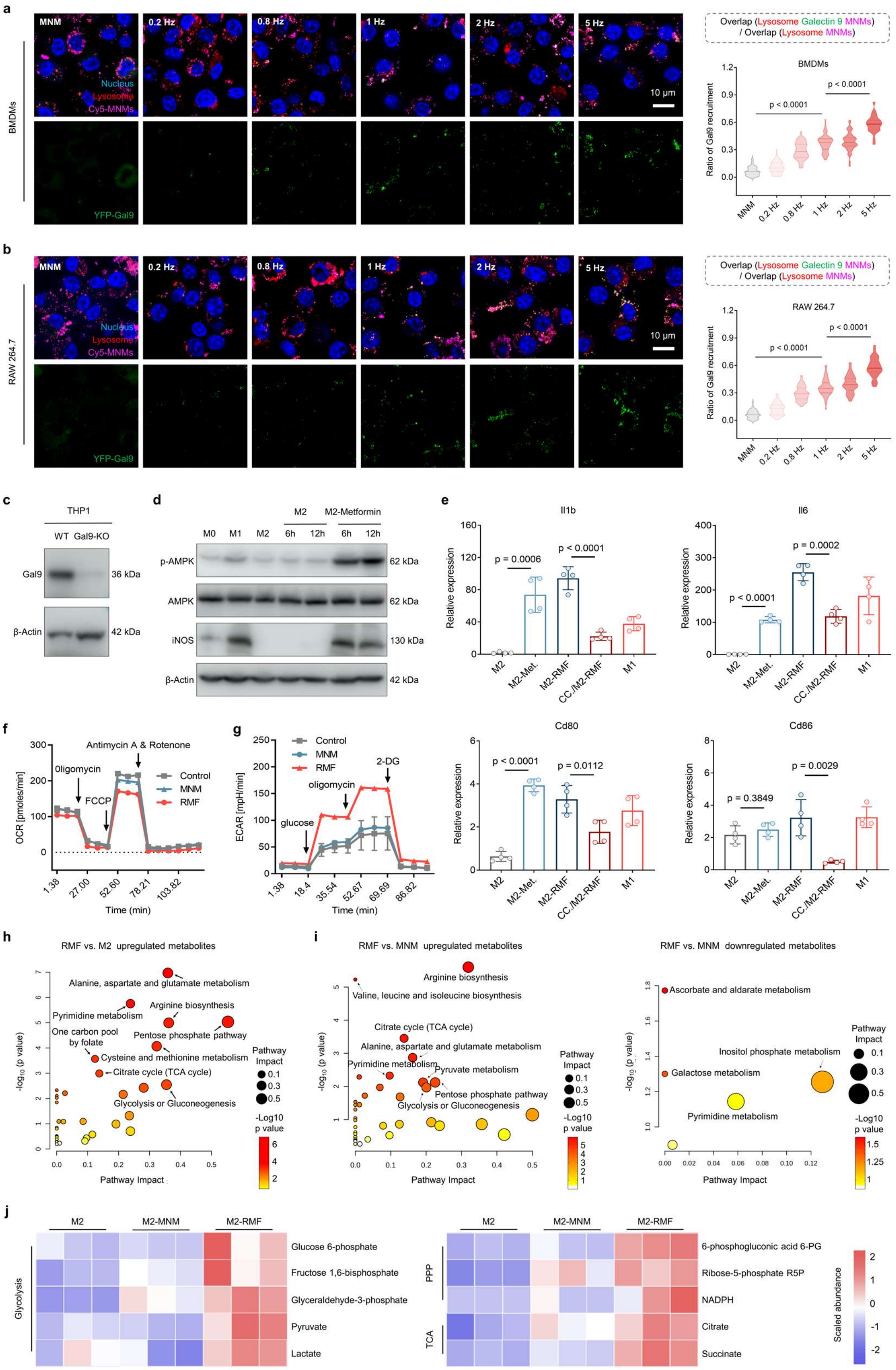

Fig. S7. Effects of Gal9/AMPK axis and metabolic levels in macrophages after MagLMP.

a, **b** YFP-Gal9-transfected BMDMs or RAW 264.7 cells were differentiated to M2 macrophages. Cells were incubated with MNMs and treated with the indicated RMF frequencies. Lysosome was stained with Lysotracker red (red), nucleus was stained with Hoechst (blue), and MNMs were modified with Cy5 (purple). Representative images and statistical analyses of Gal9 recruitment to

lysosome were shown. Data are presented as mean ± s.d. Statistical significance is defined as $p < 0.05$ (n = 60 cells from 10 independent biological replicates).

c Western blotting analysis of Gal9 in THP-1 wild type and *Gal9*-KO cells.

d RAW 264.7 cells were differentiated to M1 or M2 macrophages. M2 macrophages were incubated with or without MNMs and treated either with or without RMF of 1 Hz, Metformin, or both. Immunoblotting analysis was performed in these cells.

e RAW 264.7 cells were differentiated to M0, M1 or M2 macrophages. M2 macrophages were incubated with or without MNMs and treated with or without RMF of 1 Hz. Cells were treated with Metformin, Compound C, or neither. mRNA levels of *Cd80, Cd86, Il1b* and *Il6* were examined in these cells. Data are presented as mean ± s.d. Statistical significance is defined as $p < 0.05$ (n = 4 independent biological replicates).

f, **g** RAW 264.7 cells-polarized M0 cells were incubated with or without MNMs and treated with or without RMF of 1 Hz. Oxygen consumption rate (OCR) assay (**f**) and extracellular acidification rate (ECAR) assay (**g**) were performed by using to measure metabolic activity in these cells.

h-j RAW 264.7 cells were polarized to M2 macrophages. M2 macrophages were incubated with or without MNMs and treated with or without RMF of 1 Hz. Cells were harvested and subjected to untargeted metabolomic analyses. Pathway enrichment analyses were conducted on up- and downregulated metabolites (**h**, **i**). Heat map analysis showed differential metabolites associated with macrophage polarization (**j**). (n = 3 independent biological replicates). PPP, pentose phosphate pathway; TCA, tricarboxylic acid cycle.
