# Supplementary material for: Dynamic magneto-mechanical force in lysosomes induces durable macrophage repolarization for antitumor immunity
Source: Cell Res. 2026 Feb 3;36(3):197–218. doi: 10.1038/s41422-025-01217-1 (PMC12909937; doi:10.1038/s41422-025-01217-1)
Supplement: Supplementary file 8 — Supplementary Information, Fig. S8 [file 41422_2025_1217_MOESM8_ESM.pdf]

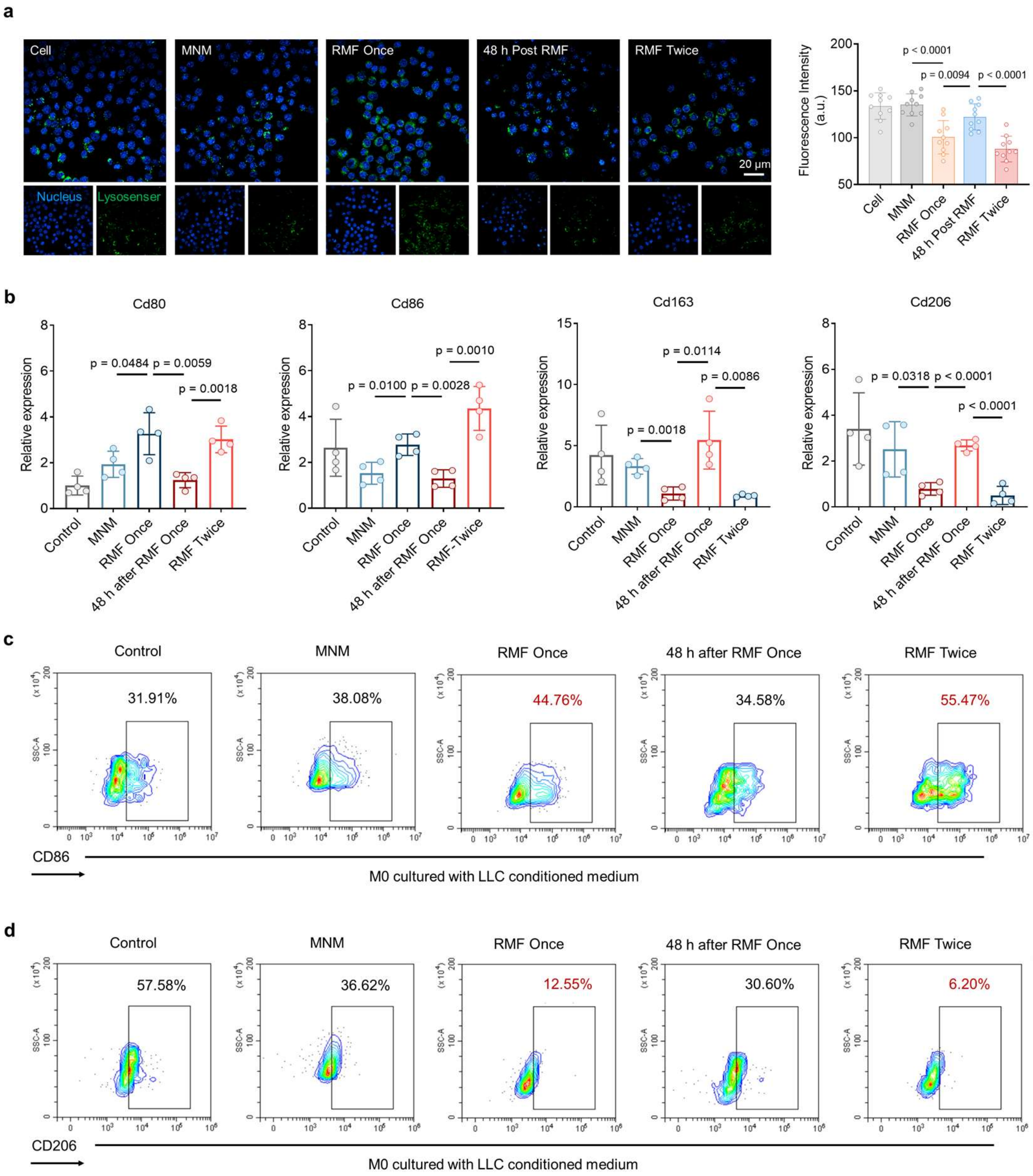

Fig. S8. Effects of sustained macrophage repolarization by the programmable MagLMP strategy.

a RAW 264.7 cells were differentiated to M2 macrophages. Cells were incubated with MNMs and treated with 1 Hz RMF for 15 min (Once). 24 h later, the cells were treated with 1 Hz RMF again (Twice). Fluorescence images of lysosomal pH and the corresponding statistics data of fluorescence intensity were shown.

b-d RAW 264.7 cells were cultured with LLC conditioned medium. Cells were incubated with or without MNMs and treated with or without 1 Hz RMF once or twice. mRNA levels of *Cd80*, *Cd86*, *Cd163*, and *Cd206* were examined in these cells (**b**). CD86 (**c**) and CD206 (**d**) expression was examined by flow cytometry analysis. Data are presented as mean ± s.d. Statistical significance is defined as $p < 0.05$ (n = 4 independent biological replicates).
