# Supplementary material for: Dynamic magneto-mechanical force in lysosomes induces durable macrophage repolarization for antitumor immunity
Source: Cell Res. 2026 Feb 3;36(3):197–218. doi: 10.1038/s41422-025-01217-1 (PMC12909937; doi:10.1038/s41422-025-01217-1)
Supplement: Supplementary file 9 — Supplementary Information, Fig. S9 [file 41422_2025_1217_MOESM9_ESM.pdf]

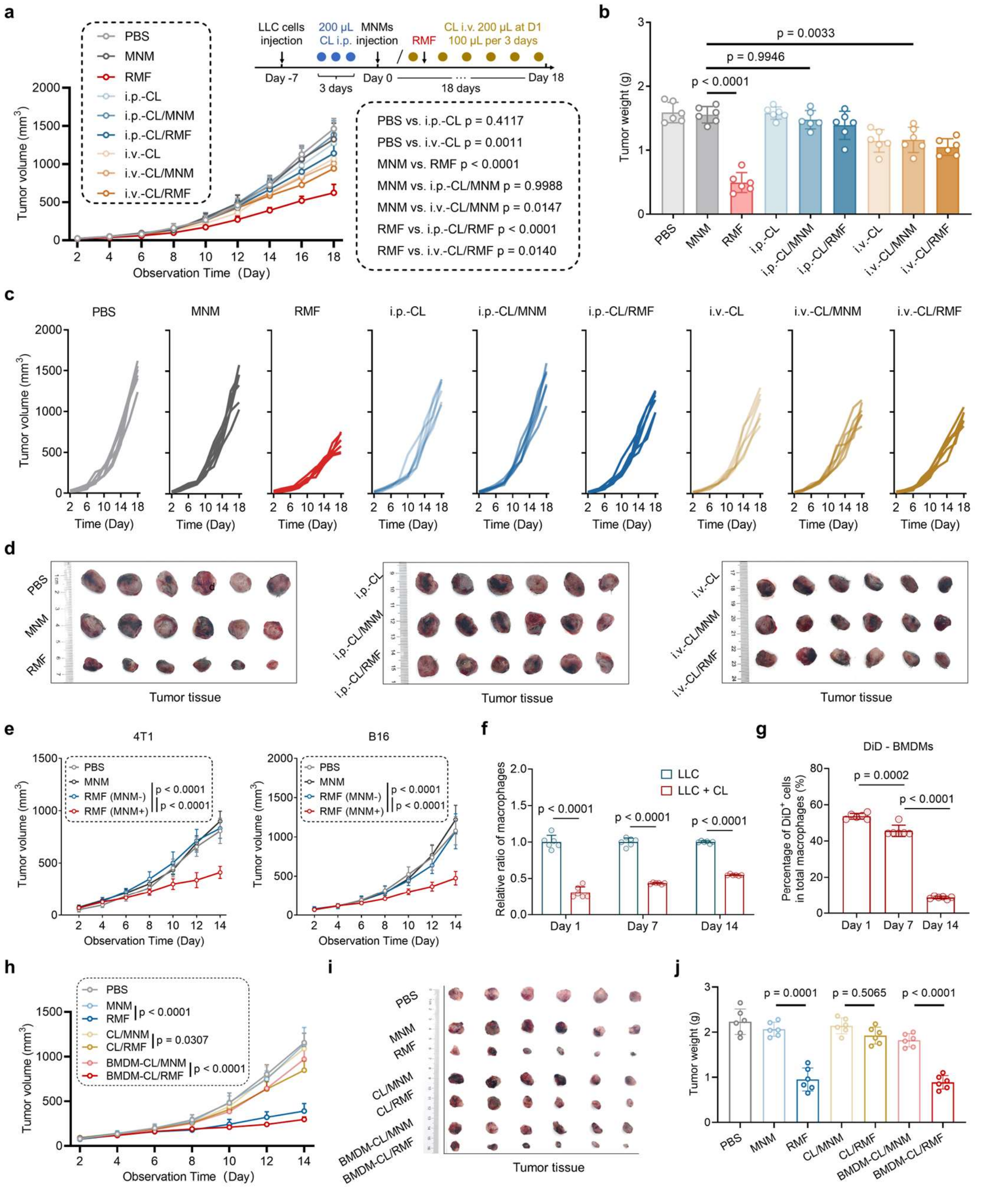

Fig. S9. Macrophage depletion verifies the essential role of macrophages in MagLMP antitumor activity.

a-d LLC cells were implanted subcutaneously into C57BL/6 mice. MNMs were injected into the tumor directly before MagLMP strategy was performed on these mice. Mice were treated with CL using two different dosing regimens: (1) i.p. injection of 200 μL once daily for three consecutive days before treatment, and (2) i.v. injection of 200 μL on day 1 followed by 100 μL every three days during the treatment period. Tumor growth over time and tumor weight were measured (**a-c**). Tumor tissue imaging was shown (**d**). Statistical significance was defined as $p < 0.05$ (n = 6 independent biological replicates).

e 4T1 and B16 cells were implanted subcutaneously into C57BL/6 mice. MNMs were injected into the tumor directly before MagLMP strategy was performed on these mice. Tumor growth over time and tumor weight were measured. Data are presented as mean ± s.d of six mice. Statistical significance is defined as $p < 0.05$.

f LLC cells were implanted subcutaneously into C57BL/6 mice. Mice were treated with or without CL, administered intraperitoneally at 200 μg once daily for three consecutive days. Flow cytometry analysis of tumor macrophages ($CD45^+$, $F4/80^+$, $CD11b^+$) was performed on days 1, 7 and 14. Data are presented as mean ± s.d of six mice. Statistical significance is defined as $p < 0.05$.
g LLC cells were implanted subcutaneously into C57BL/6 mice. BMDMs were isolated and differentiated from WT mice, stained with the DiD membrane dye for 15 min, and adoptively transferred into tumors. Flow cytometry analysis of tumor DiD-BMDMs ($CD45^+$, $F4/80^+$, $CD11b^+$, DiD^+) was performed on days 1, 7 and 14. Data are presented as mean ± s.d of six mice. Statistical significance is defined as $p < 0.05$.
h-j LLC cells were implanted subcutaneously into C57BL/6 mice. MNMs were injected into the tumor directly before MagLMP strategy was performed on these mice. Mice were treated with or without CL. BMDMs were isolated and differentiated from WT mice, and co-incubated with MNMs for 24 h before being adoptively transferred into the tumor tissue. Mice were treated with or without RMF (30 min per day). Tumor growth over time (**h**), tumors (**i**) and tumor weight (**j**) were measured. Data are presented as mean ± s.d of six mice. Statistical significance is defined as $p < 0.05$.
