# Supplementary material for: Dynamic magneto-mechanical force in lysosomes induces durable macrophage repolarization for antitumor immunity
Source: Cell Res. 2026 Feb 3;36(3):197–218. doi: 10.1038/s41422-025-01217-1 (PMC12909937; doi:10.1038/s41422-025-01217-1)
Supplement: Supplementary file 10 — Supplementary Information, Fig. S10 [file 41422_2025_1217_MOESM10_ESM.pdf]

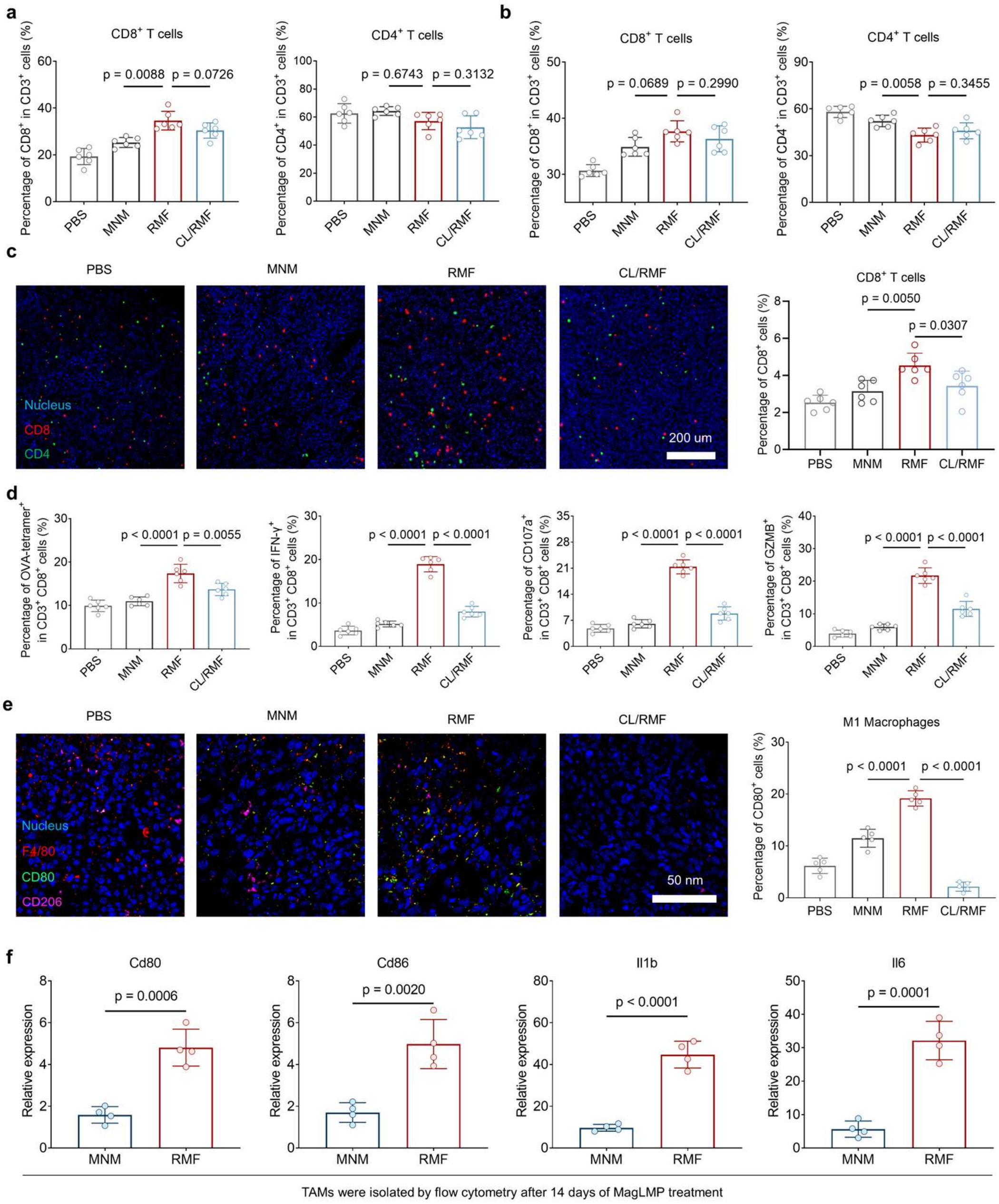

Fig. S10. Application of MagLMP in other tumor models and validation of the immune activation effects.

a-e LLC cells were implanted subcutaneously into C57BL/6 mice. MNMs were injected into the tumor directly before MagLMP strategy was performed on these mice. Mice were treated with or without CL. Flow cytometry analysis of $CD8^+$ or $CD4^+$ T cells in tumor tissues (**a**) and spleen tissues (**b**) from these mice was performed. Immunofluorescence staining of CD8 (red) and CD4 (green) was performed on tumor tissues using the corresponding antibodies. Representative images and statistical results were shown (**c**). Flow cytometry analysis of OVA-tetramer$^+$, IFN-γ^+, CD107a$^+$ and GZMB$^+$ in $CD3^+CD8^+$ T cells was performed in spleens (**d**). F4/80 (red), CD206 (purple) and CD80 (green) were stained with the related antibodies in tumor tissues. Representative images and statistical results were shown (**e**). Data are presented as mean ± s.d. of six mice. Statistical significance is defined as $p < 0.05$.

f LLC cells were implanted subcutaneously into C57BL/6 mice. After 14 days of MagLMP

treatment, TAMs ($CD45^+$, $CD11b^+$, $F4/80^+$) were isolated by flow cytometric sorting. mRNA levels of *Cd80*, *Cd86*, *Il1b* and *Il6* in tumor tissues were examined. Data are presented as means ± s.d of six mice. Statistical significance is defined as $p < 0.05$.
