# Supplementary material for: Dynamic magneto-mechanical force in lysosomes induces durable macrophage repolarization for antitumor immunity
Source: Cell Res. 2026 Feb 3;36(3):197–218. doi: 10.1038/s41422-025-01217-1 (PMC12909937; doi:10.1038/s41422-025-01217-1)
Supplement: Supplementary file 11 — Supplementary Information, Fig. S11 [file 41422_2025_1217_MOESM11_ESM.pdf]

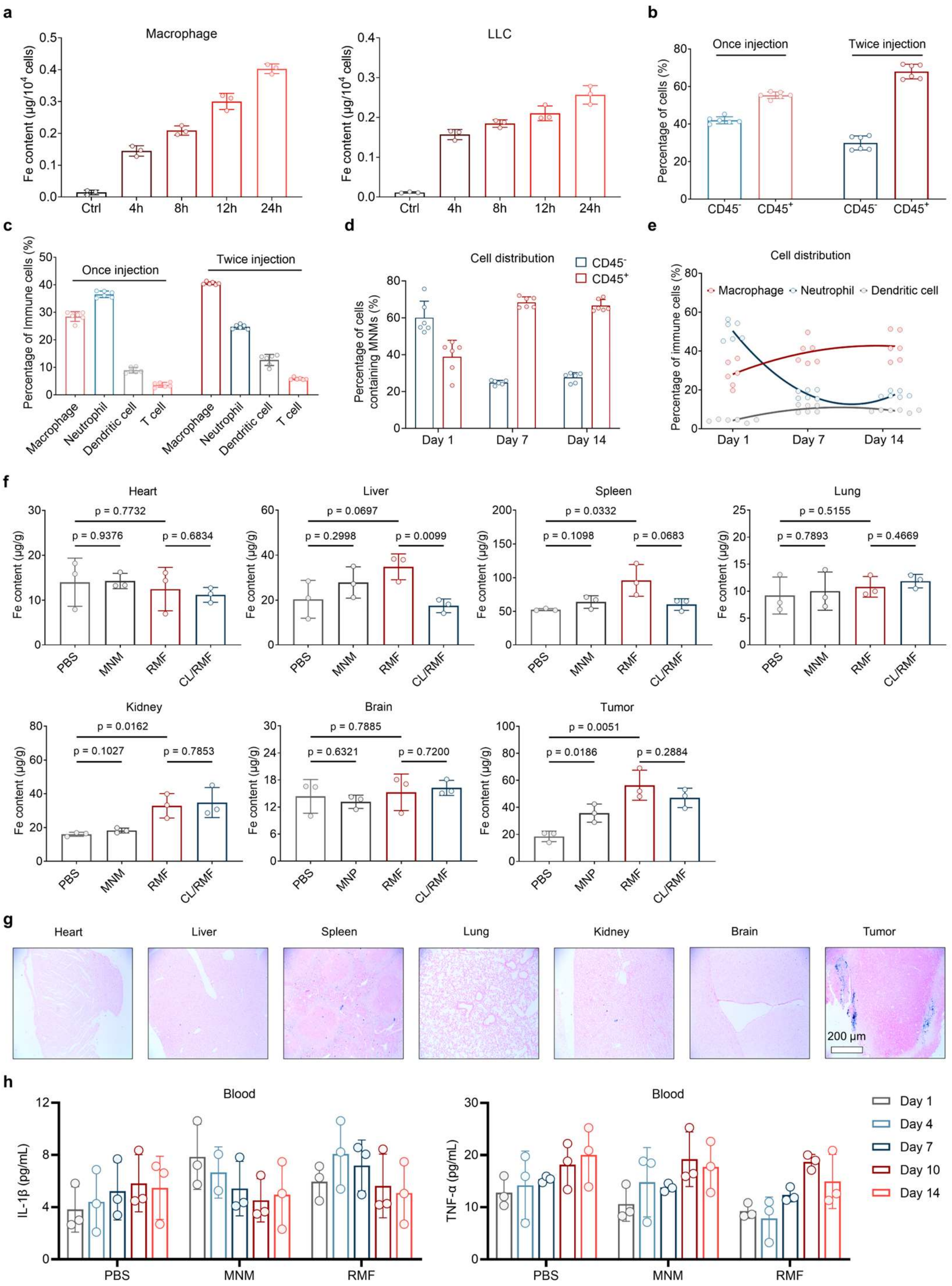

Fig. S11. Uptake efficiency of MNMs and Safety assessment of MagLMP.

a Iron content in RAW 264.7 and LLC cells incubated with 20 μg/mL MNMs for 4 h, 8 h, 12 h or 24 h was determined by ICP-MS. Data are presented as means ± s.d. Statistical significance is defined as $p < 0.05$ (n = 3 independent biological replicates).

b-h LLC cells were implanted subcutaneously into C57BL/6 mice. MNMs were injected into the tumor directly. 14 days after RMF treatment, tumors were dissected and the cells containing MNMs in tumors were isolated by magnetic separation. Flow cytometry analysis of different cell types was performed with MNM-containing cells (**b-e**). Iron content in heart, liver, spleen, lung, kidney, brain and tumor tissue in these mice was examined (**f**). Prussian blue staining of tumors and different

organs in RMF group were shown (**g**). The concentration of IL-1β and TNF-α in the blood of these mice was examined (**h**). Data are presented as mean ± s.d. Statistical significance is defined as $p < 0.05$.
