# Supplementary material for: Dynamic magneto-mechanical force in lysosomes induces durable macrophage repolarization for antitumor immunity
Source: Cell Res. 2026 Feb 3;36(3):197–218. doi: 10.1038/s41422-025-01217-1 (PMC12909937; doi:10.1038/s41422-025-01217-1)
Supplement: Supplementary file 12 — Supplementary Information, Fig. S12 [file 41422_2025_1217_MOESM12_ESM.pdf]

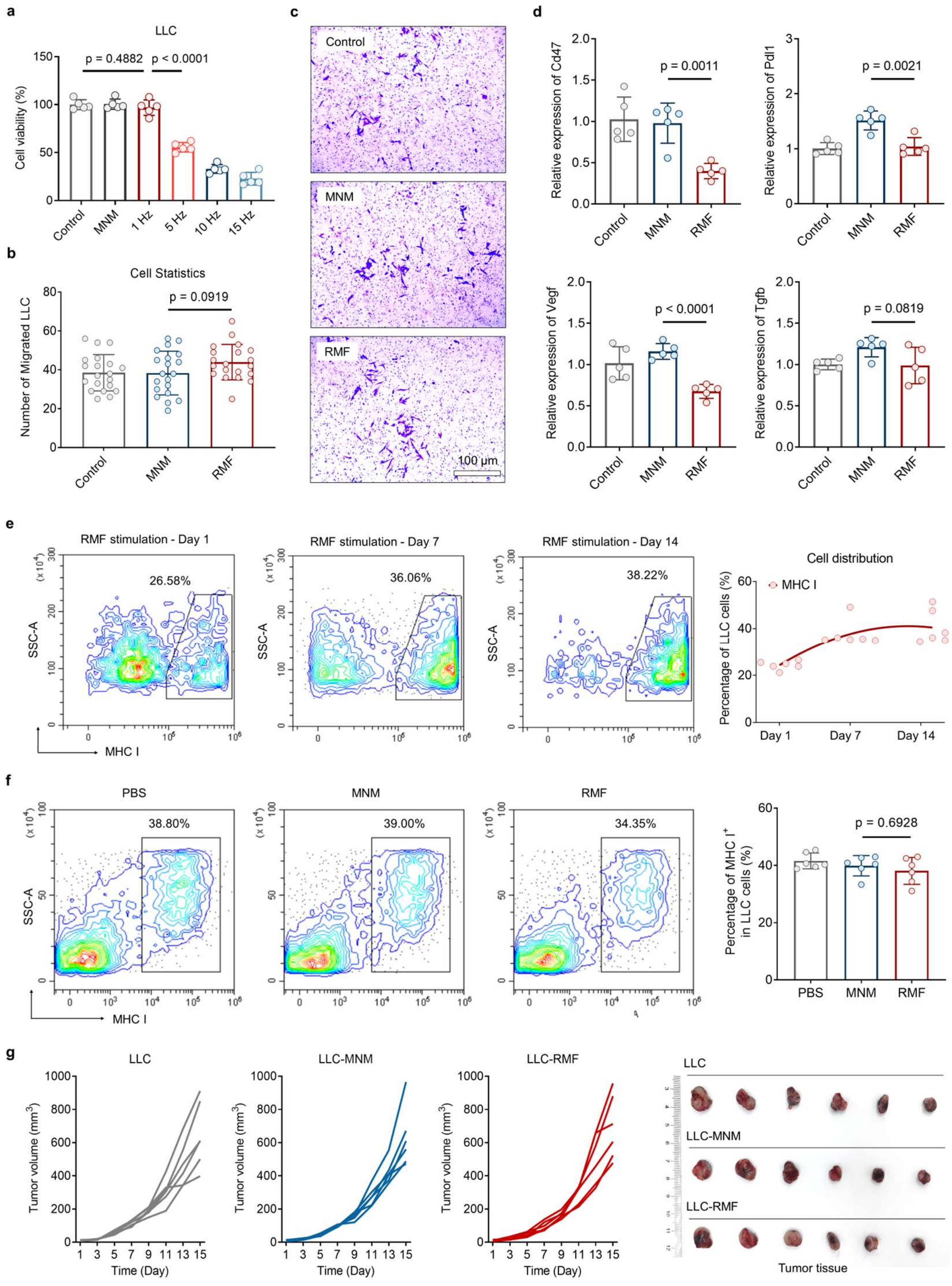

Fig. S12. Effects of LLC tumor cells by the programmable MagLMP strategy.

a LLC cells were incubated with MNMs and treated with or without RMF. Cell viability was measured under different frequencies of RMF stimulation. Data are presented as mean ± s.d. Statistical significance is defined as $p < 0.05$ (n = 5 independent biological replicates).

b, **c** LLC cells were incubated with MNMs and treated with or without RMF. Cell migration was examined using Transwell chambers (**b**). Representative images were shown (**c**). Data are presented as mean ± s.d. Statistical significance is defined as $p < 0.05$ (n = 20 independent samples in Fig. **b**).

d LLC cells were incubated with MNMs and treated with or without RMF. mRNA levels of *Cd47, Pdl1, Vegf* and *Tgfb* were examined in these cells. Data are presented as mean ± s.d. Statistical

significance is defined as $p < 0.05$ (n = 5 independent biological replicates).

e GFP-LLC cells were implanted subcutaneously into C57BL/6 mice. MNMs were injected into the tumor directly, followed by RMF treatment for 14 days. Flow cytometry analysis of GFP^+ and MHC I^+ cells in tumor tissues and statistical results were shown on days 1, 7 and 14. Data are presented as mean ± s.d. of six mice. Statistical significance is defined as $p < 0.05$.

f LLC cells were incubated with MNMs and then treated by 1 Hz RMF for 30 min. Flow cytometry analysis of MHC I^+ expression in these cells and statistical results were shown. Data are presented as mean ± s.d. Statistical significance is defined as $p < 0.05$ (n = 6 independent biological replicates).

g LLC cells were incubated with MNMs and then implanted subcutaneously into C57BL/6 mice. MagLMP strategy was performed on these mice for 14 days. Tumor growth over time was measured. 14 days after RMF treatment (30 min per day), tumors were dissected.
