# Supplementary material for: Dynamic magneto-mechanical force in lysosomes induces durable macrophage repolarization for antitumor immunity
Source: Cell Res. 2026 Feb 3;36(3):197–218. doi: 10.1038/s41422-025-01217-1 (PMC12909937; doi:10.1038/s41422-025-01217-1)
Supplement: Supplementary file 13 — Supplementary Information, Fig. S13 [file 41422_2025_1217_MOESM13_ESM.pdf]

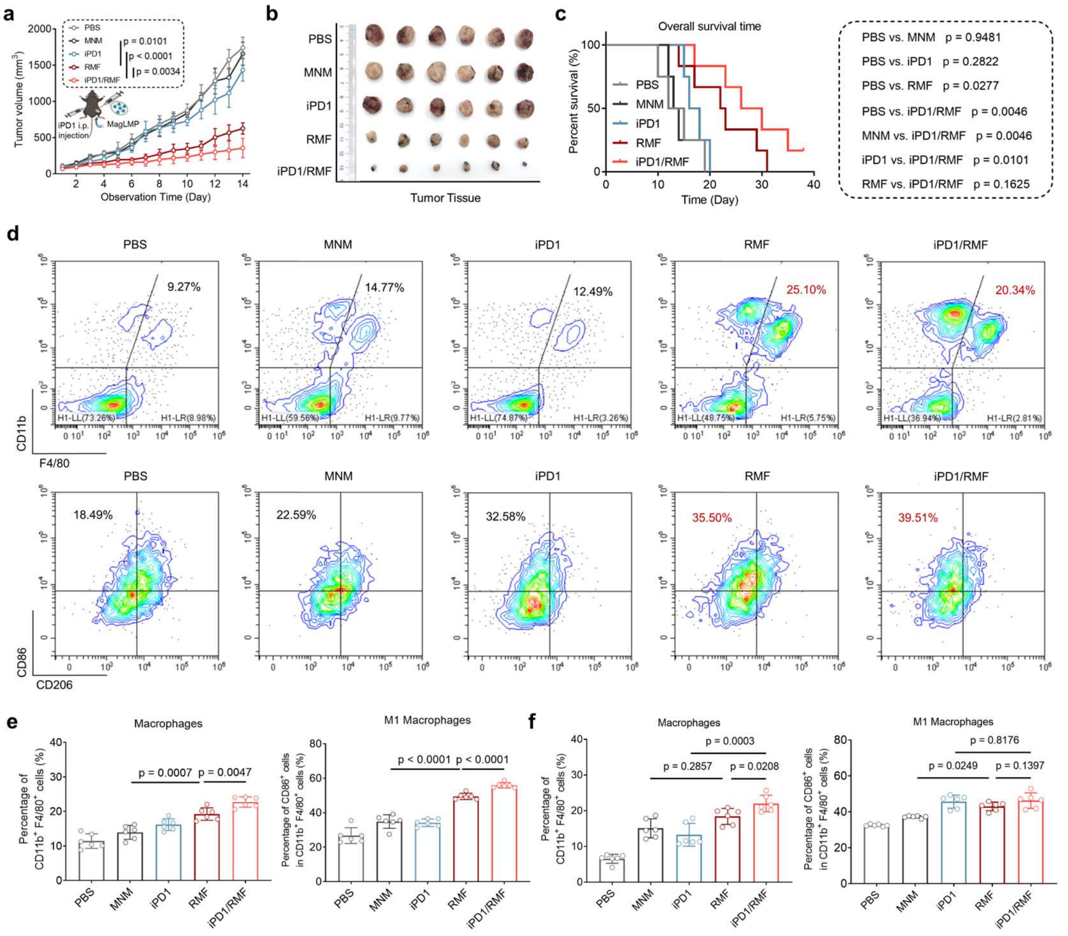

Fig. S13. The combination strategy of MagLMP and anti-PD1 antibody for antitumor immunity.

a-f Mouse-derived allograft of LLC cells was dissected and implanted subcutaneously into C57BL/6 mice. MNMs were injected into the tumor directly before MagLMP strategy was performed on these mice. Mice were treated with or without anti-PD1 antibody. Tumor growth over time was measured at 14 days after RMF treatment (**a**), tumors were dissected (**b**). Kaplan-Meier survival analysis was also performed (**c**). Flow cytometry analysis of macrophages ($F4/80^+$, $CD11b^+$) and M1 macrophages ($CD86^+$, $CD206^-$ in $F4/80^+$, $CD11b^+$ cells) was performed in tumor tissues (**d**, **e**) and spleen tissues (**f**) from these mice was performed. Data are presented as mean ± s.d. of six mice. Statistical significance is defined as $p < 0.05$.
