# Supplementary material for: Dynamic magneto-mechanical force in lysosomes induces durable macrophage repolarization for antitumor immunity
Source: Cell Res. 2026 Feb 3;36(3):197–218. doi: 10.1038/s41422-025-01217-1 (PMC12909937; doi:10.1038/s41422-025-01217-1)
Supplement: Supplementary file 14 — Supplementary Information, Fig. S14 [file 41422_2025_1217_MOESM14_ESM.pdf]

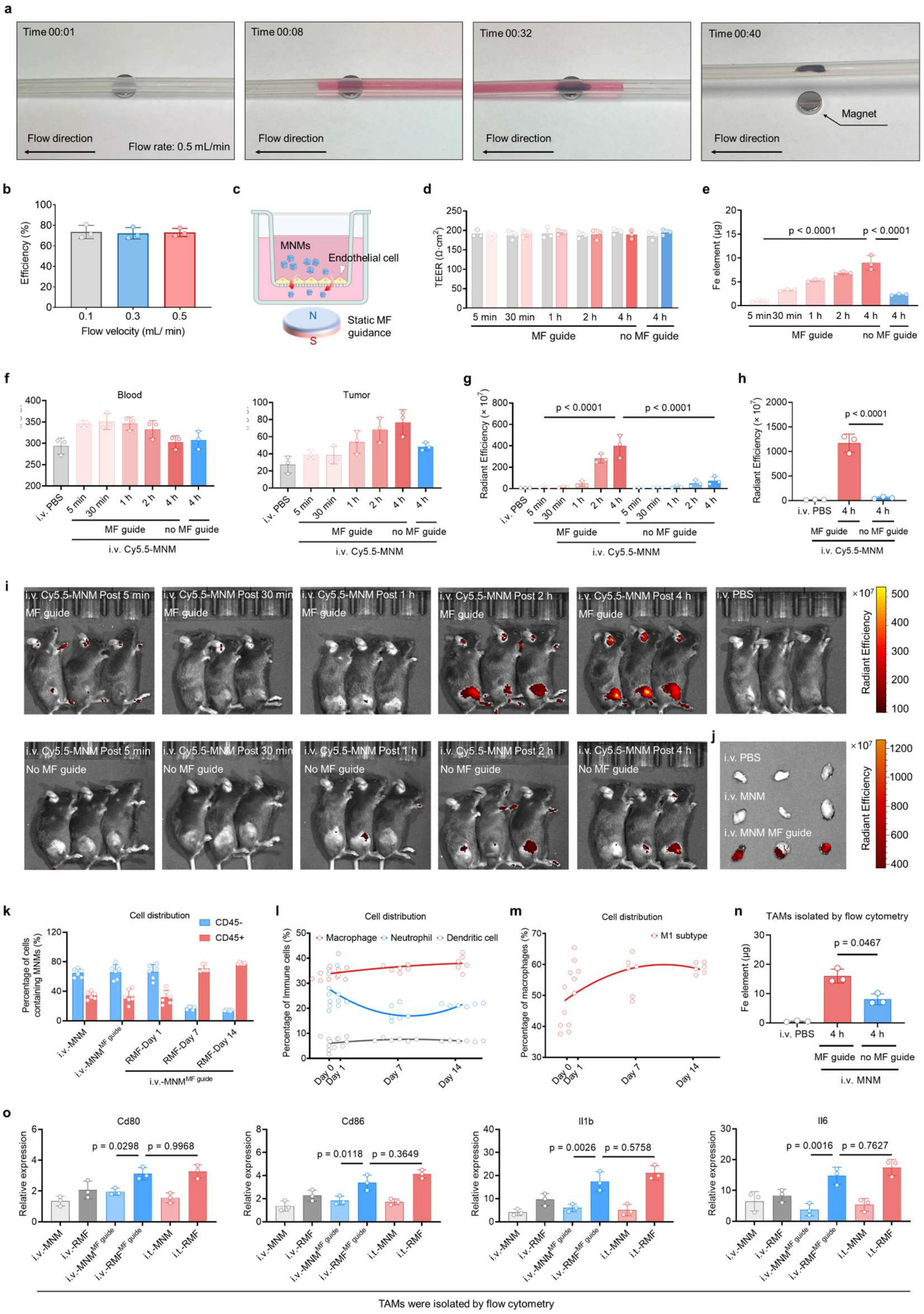

Fig. S14. Tumor enrichment of intravenously injected MNMs under MF guidance and activation of TAMs under RMF stimulation.

a, **b** Representative images of magnet-guided enrichment of MNMs at 0.5 mL/min (**a**) and the corresponding enrichment efficiencies at 0.1, 0.3, and 0.5 mL/min (**b**), which approximate the flow velocities in veins, medium-sized arteries, and large arteries, respectively. A peristaltic pump system with artificial blood was used to simulate venous flow conditions. See **Supplementary information, Video S15** for details.

c-e Schematic of a Transwell-based endothelial monolayer model and magnet-induced transendothelial transport of MNMs (**c**). Transendothelial electrical resistance (TEER) was measured to confirm monolayer integrity (**d**). Iron content in the lower chamber was quantified after different durations of magnetic exposure (**e**). Data are presented as mean ± s.d. Statistical significance is defined as $p < 0.05$ (n = 3 independent biological replicates).

f-m LLC cells were implanted subcutaneously into wild type C57BL/6 mice. MNMs were injected intravenously (i.v.) into the tumor, followed by the placement of a magnet over the tumor to guide MNM accumulation. Iron content of blood and tumor tissues at different time points were determined (**f**). Statistical analysis of signal intensity and representative *in vivo* fluorescence imaging (**g, i**) and *ex vivo* tumor tissue images (**h, j**). Flow cytometry analysis of different cell types in tumor tissues (**k-m**) were shown. Data are presented as mean ± s.d. of three mice. Statistical significance is defined as $p < 0.05$.

n, o LLC cells were implanted subcutaneously into C57BL/6 mice. MNMs were injected intravenously (i.v.) or intratumorally (i.t.) into the tumor, followed by the placement of a magnet over the tumor to guide MNM accumulation. TAMs ($CD45^+$, $CD11b^+$, $F4/80^+$) were isolated by flow cytometric sorting. Iron content of TAMs was determined (**n**). mRNA levels of *Cd80*, *Cd86*, *Il1b* and *Il6* in tumor tissues were examined (**o**). Data are presented as means ± s.d. of three mice. Statistical significance is defined as $p < 0.05$.
