# Supplementary material for: Dynamic magneto-mechanical force in lysosomes induces durable macrophage repolarization for antitumor immunity
Source: Cell Res. 2026 Feb 3;36(3):197–218. doi: 10.1038/s41422-025-01217-1 (PMC12909937; doi:10.1038/s41422-025-01217-1)
Supplement: Supplementary file 15 — Supplementary Information, Fig. S15 [file 41422_2025_1217_MOESM15_ESM.pdf]

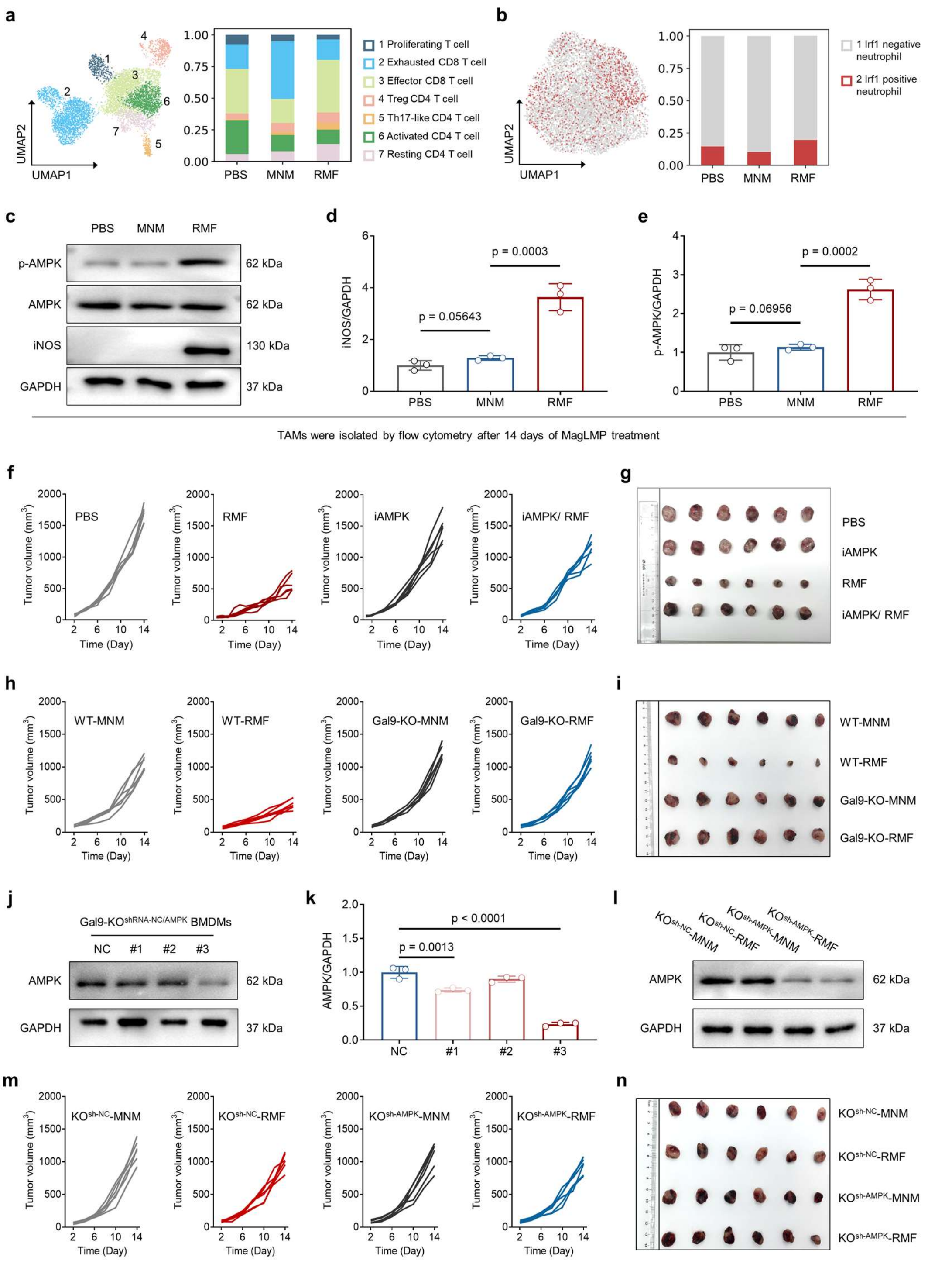

Fig. S15. Effects of MagLMP-induced macrophage repolarization and Gal9-AMPK signaling pathway for antitumor immunity *in vivo*.

a, b LLC cells were implanted subcutaneously into C57BL/6 mice. MNMs were injected into the tumor directly before MagLMP strategy was performed on these mice. Single cell sequencing was performed with these tumors. Uniform manifold approximation and projection (UMAP) plot and ratio of major T cell types (**a**) and neutrophil types (**b**) in tumor tissues were shown. Data are presented as mean (three mice per group).

c-e LLC cells were implanted subcutaneously into C57BL/6 mice. MNMs were injected into the tumor directly before MagLMP strategy was performed on these mice. TAMs ($CD45^+$, $CD11b^+$,

F4/80$^+$) were isolated by flow cytometric sorting. Western blotting analysis was performed in tumor tissues from these mice. Statistical analysis of iNOS/GAPDH and p-AMPK/GAPDH was shown. Data are presented as mean ± s.d. Statistical significance is defined as $p < 0.05$ (n = 3 technical replicates).

f, **g** Mouse-derived allograft of LLC cells was dissected and implanted subcutaneously into wild type C57BL/6 mice. Mice were treated with or without RMF and/or AMPK inhibitor. Tumor growth over time was measured (**f**). 14 days after RMF treatment, tumors were dissected (**g**).

h, **i** Mouse-derived allograft of LLC cells was dissected and implanted subcutaneously into C57BL/6 mice. BMDMs were isolated and differentiated from WT and *Gal9*-KO mice, respectively, and co-incubated with MNMs for 24 h before being adoptively transferred into the tumor tissues. Mice were pretreated with CL and then stimulated with or without RMF (30 min per day). Tumor growth over time was measured (**h**). 14 days after RMF treatment, tumors were dissected (**i**). Data are presented as mean ± s.d of six mice. Statistical significance is defined as $p < 0.05$.

j, k Western blotting analysis of AMPK in BMDMs isolated and differentiated from *Gal9*-KO mice. Cells were transduced with lentiviruses encoding either NC- or AMPK-shRNA (#1, #2, #3). Statistical analysis of AMPK/GAPDH was shown. Data are presented as mean ± s.d. Statistical significance is defined as $p < 0.05$ (n = 3 technical replicates).

l-n Mouse-derived allograft of LLC cells was dissected and implanted subcutaneously into C57BL/6 mice. BMDMs were isolated and differentiated from *Gal9*-KO mice, and subsequently transduced with lentiviruses encoding either NC- or AMPK-shRNA. Cells were then co-incubated with MNMs for 24 h before being adoptively transferred into the tumor tissues. Mice were pretreated with CL and then stimulated with or without RMF (30 min per day). Western blot analysis of adoptively transferred BMDMs (with GFP reporter) sorted from tumor tissues by flow cytometry on day 7 (**l**). Tumor growth over time was measured (**m**). 14 days after RMF treatment, tumors were dissected (**n**). Data are presented as mean ± s.d of six mice. Statistical significance is defined as $p < 0.05$.
