# Supplementary material for: Dynamic magneto-mechanical force in lysosomes induces durable macrophage repolarization for antitumor immunity
Source: Cell Res. 2026 Feb 3;36(3):197–218. doi: 10.1038/s41422-025-01217-1 (PMC12909937; doi:10.1038/s41422-025-01217-1)
Supplement: Supplementary file 31 — Supplementary Information, Video legends [file 41422_2025_1217_MOESM31_ESM.pdf]

Supplementary video legends

Video S1. Fluidic vortex generated by rotational motion of MNM assemblies, visualized using polystyrene nanospheres.

Video S2. The rotation of assembled magnetic nanomotors in lysosomes of macrophages under RMF stimulation.

Video S3, 4. The rotation of assembled magnetic nanomotors in lysosomes of macrophages under RMF stimulation. Lysosome was stained with Lyso-tracker Red (red) and nucleus was stained with Hoechst (blue).

Video S5-8. The rotation of assembled magnetic nanomotors in lysosomes of macrophages under RMF stimulation at the field strength of 4 mT, 8 mT, 12 mT and 20 mT. The lysosome was stained with Lyso-tracker Red (red) and nucleus was stained with Hoechst (blue).

Video S9-13. Changes of Gal3 in EGFP-Gal3-transfected macrophages post RMF stimulation at the frequency of 0.2 Hz, 0.8 Hz, 1 Hz, 2 Hz, 5 Hz for 15 min.

Video S14. Changes of Gal3 in EGFP-Gal3-transfected macrophages post chloroquine stimulation for 1 h.

Video S15. Magnetic field-guided local enrichment of MNMs in a fluidic environment (flow rate: 0.5mL/min).
