# Supplementary material for: Dynamic magneto-mechanical force in lysosomes induces durable macrophage repolarization for antitumor immunity
Source: Cell Res. 2026 Feb 3;36(3):197–218. doi: 10.1038/s41422-025-01217-1 (PMC12909937; doi:10.1038/s41422-025-01217-1)
Supplement: Supplementary file 32 — Supplementary Information, Materials and Methods [file 41422_2025_1217_MOESM32_ESM.pdf]

Supplementary information, Materials and Methods

Theoretical calculation and finite element simulation within lysosomes

Theoretical calculation of assembled number and the torque of assembled MNMs.

Magnetic nanoparticles would form into assemblies with the magnetic field. When the magnetic field rotates, the MNMs are subjected to the magnetic moment and resistance of environment. The number and magnetic moment of the assembly are affected by many factors, including speed, magnetic field strength and solution viscosity. It is assumed that the assembly number of MNMs is N in the rotating magnetic field with rotation frequency f, and the inertial force can be ignored in the low Reynolds number environment. Therefore, the assembly is mainly affected by the magnetic moment τ_{mag} and the friction moment τ_{fric} during rotation. In order to calculate the number of assemblies, we analyze the force of the Nth particle in the assembly to judge its stability in the system. Since the frequency of the rotating magnetic field used is lower than the out-of-step frequency of the MNMs, the material rotates at a speed synchronized with the magnetic field. Thus, the stable existence of the Nth particle in the system includes the following three conditions:

1) Attractive force balances "centrifugal force":

$$F_{ipa} - F_{cf} \geq 0 \quad (1)$$

2) Inter-particle friction force balances fluid resistance force:

$$F_{fr} \leq F_f \quad (2)$$

$$F_f = \varepsilon(F_{ipa} - F_{cf}) \quad (3)$$

3) Torque by fluid force or magnetic field is not excessive:

$$| F_{fr} - \frac{2\tau_{mag-1}}{l_c} | + F_{cf} \leq F_{ipa} \quad (4)$$

In which, F_{ipa}, F_{cf}, F_{fr}, F_f, τ_{mag-1} represent inter-particle attractive forces, centrifugal force, fluid resistance force, frictional force and magnetic torque of the Nth particle respectively. The calculation methods for these forces are as follow [58-60]:

$$F_{ipa} = \frac{3\mu_0 \rho l_c^3 M \rho (N-1) l_c^3 M}{2\pi(\frac{N}{2} l_c)^4} \quad (5)$$

$$F_{cf} = m\omega^2 r = \rho l_c^3 (2\pi f)^2 \frac{(N-1)l_c}{2} \quad (6)$$

$$F_{fr} = \frac{64\pi(\frac{l_c}{2})^3}{3} \frac{N^3}{l_n N + \frac{1.2}{N}} \eta\omega \frac{1}{(N-1)\frac{l_c}{2}} \quad (7)$$

$$\tau_{mag-1} = B_0 l_c^3 M \sin\alpha \quad (8)$$

In which, $\mu_0 = 4\pi \times 10^{-7}$ is the permeability of vacuum, ρ is the density of the particle, m is the mass of one particle, l_c is the length of one particle, M is the magnetization of the particle under external magnetic field, η is the viscosity of lysosome, B_0 is the magnetic intensity of magnetic field. For the whole stable assembly rotation system:

$$\tau_{mag} = \tau_{fric} \quad (9)$$

$$\tau_{mag} = B_0 N l_c^3 M \sin\alpha \quad (10)$$

We assume that the rotating bodies, consisting of a group of Nth magnetic cubes, molecular coating of the cubes and adhered molecules, have a geometry which is reasonably approximated by an ellipsoid. At low Reynolds number (Re << 1) condition, the friction torque exerted by viscous fluid on a rotating ellipsoidal rod may be computed using the equation obtained by Chwang and Wu in 1975 [61]:

$$\tau_{fric} = C_{MR} 8\pi\eta \frac{N l_c}{2} (\frac{l_c}{2})^2 \sin\alpha \quad (11)$$

The coefficient C_{MR} is given by:

$$C_{MR} = \frac{4}{3} e^3 (\frac{2-e^2}{1-e^2})[-2e + (1 + e^2)\ln(\frac{1+e}{1-e})]^{-1} \quad (12)$$

In which, the eccentricity e is given by:

$$e = [1 - (\frac{1}{N})^2]^{1/2} \quad (13)$$

Thus, according to formula (8), we get:

$$\sin\alpha = \frac{2\pi^2 f C_{MR} \eta}{B_0 M} \quad (14)$$

By calculating the three equilibrium formulas (1), (2) and (4), we get the maximum number of assemblies at different rotation frequencies. By calculating the formula (10), we get the magnetic torque on the assembly.

Detection of lysosomal parameters

To obtain the average radial dimension of lysosomes, FITC-Dextran (MedChemExpress, 60842-46-8) was utilized to label lysosomes in RAW 264.7 cells. After macrophages were seeded on Φ 35 mm confocal dishes with a concentration of 1×10^5 cells per dish and cultured for 24 h, 20 μg/mL MNMs were added and cocultured for 24h. Then, FITC-Dextran (5 μg/mL) were cocultured for 4 h. Cells were washed with PBS for twice with PBS and nuclei were stained with Hoechst 33342 (blue, Dojindo Molecular Technologies, 23491-52-3) in DMEM medium at a ratio of 1:500 for 15 min at 37°C, followed by fixing with 4% paraformaldehyde (PFA) for 15 min. Z-stack technique of confocal laser scanning microscopy (Leica TCS SP8) was used to construct the 3D structure of lysosomes in macrophages.

Besides, the average fluid viscosity of lysosomes in macrophages was detected by DCVJ (MedChemExpress, 58293-56-4). 1×10^8 RAW 264.7 cells were collected and lysosomes were extracted by the lysosome extraction kit (Solarbio, EX2670). Next, the membrane was destroyed by ultrasound and the inner fluid of lysosomes was collected. The solutions with different viscosities were prepared by a mixture of ddH_2O and glycerin and ddH_2O (volume ratio: 55%, 60%, 65%, 70%, 75%, 80%, 85%, 90%,) and detected by the rotary viscometer (LICHEN Co., Ltd., Shanghai, China) for obtaining the standard curve of viscosity and fluorescence intensity after mixing with DCVJ. The viscosity of both intra-lysosomal fluid and the mixed solution were stained by DCVJ at a ratio of 1:10 for 5 min at 37°C. Finally, the fluorescence intensity was detected by the multimode microplate reader (Tecan Austria GmbH, Austria) and the average viscosity of the lysosomes was obtained according to the standard curve.

Simulation of membrane pressure change by MagLMP with different frequencies

The commercial finite element software ANSYS 21.0 was utilized to investigate the effect of rotating assembled MNMs (the side length of each MNM = 25 nm) on lysosomal membrane. First, the Fluent module was used to simulate the flow fields generated by the assembled MNMs inside lysosomes with radial dimensions of 359.2 nm and 453.0 nm, respectively. The assembled MNMs rotated in a low Reynolds Number regime, where viscous forces dominate over inertial forces. Therefore, the Navier-Stokes equation, which neglects the inertia term, could be used to simulate the evolution of the fluid flow in the lysosome, assuming the lysosomal fluid to be an incompressible liquid. Based on bio-TEM images, the center of the assembled MNMs was assumed to coincide

with the center of the lysosome. Meanwhile, the assembled MNMs were treated as a rigid body due to their significantly higher stiffness than the lysosomal fluid, and the position of assembled MNMs remained fixed, which means it could only rotate around its short axis at a fixed position. The lysosomal fluid viscosity is set to the experimentally measured average value of 471.1 cp. The lysosomal fluid density was set as 1,000 kg/m^3.

The flow states of the lysosomal fluid for different magnetic frequencies and assembled numbers of MNMs were calculated (10 at 0.2 Hz, 7 at 0.8 Hz and 1 Hz, 6 at 2 Hz, and 5 at 5 Hz) by using the formulas and parameters mentioned above. To meet the requirements of computational convergence regarding the aspect ratio and skewness of elements, dynamic mesh was adopted to partition the fluid domain and capture curvature was enabled at the interface between fluid and solid. During fluid computation, the time step size was set to 0.001 s, the maximum iterations step was set to 30, and the number of time steps was set according to the convergence of the calculation result.

Second, after obtaining the flow velocity inside lysosomes, the fluid shear stress tensor was calculated using the following formula:

$$\begin{bmatrix} \tau_{xx} & \tau_{xy} & \tau_{xz} \\ \tau_{yx} & \tau_{yy} & \tau_{yz} \\ \tau_{zx} & \tau_{zy} & \tau_{zz} \end{bmatrix} = \mu \begin{bmatrix} \frac{\partial u}{\partial x} & \frac{1}{2}\left(\frac{\partial u}{\partial y}+\frac{\partial v}{\partial x}\right) & \frac{1}{2}\left(\frac{\partial u}{\partial z}+\frac{\partial w}{\partial x}\right) \\ \frac{1}{2}\left(\frac{\partial u}{\partial y}+\frac{\partial v}{\partial x}\right) & \frac{\partial v}{\partial y} & \frac{1}{2}\left(\frac{\partial v}{\partial z}+\frac{\partial w}{\partial y}\right) \\ \frac{1}{2}\left(\frac{\partial u}{\partial z}+\frac{\partial w}{\partial x}\right) & \frac{1}{2}\left(\frac{\partial v}{\partial z}+\frac{\partial w}{\partial y}\right) & \frac{\partial w}{\partial z} \end{bmatrix}$$

Where μ is the fluid viscosity, u, v, w are the components of the flow velocity vector $\boldsymbol{u}$ in the x, y, and z directions, respectively. Then the scalar shear stress τ was calculated using the following formula:

$$\tau = \sqrt{\frac{1}{6}\left[\left(\tau_{xx}-\tau_{yy}\right)^2+\left(\tau_{xx}-\tau_{zz}\right)^2+\left(\tau_{yy}-\tau_{zz}\right)^2\right]+\left(\tau_{xy}^2+\tau_{yz}^2+\tau_{xz}^2\right)}$$

Finally, all the computed results were imported into the CFD-Post module for visualization.

Statistical results of the assembled number of nanomotors in bio-TEM images by deep learning system

In the test, yolov7 was used as the object detection algorithm, and the operating system was Ubuntu 20.04 LTS. GPU was Tesla V10 and CUDA V11.1, and the deep learning framework was pytorch1.13. The stochastic gradient descent (SGD) algorithm was used to the optimizer. 300 epochs were trained by SGD. The size of the training image was set as 640 × 640 pixels, and the initial learning rate was 0.01. The minimum learning rate was 0.0001, and the batch size was set as 32. In the initial training of the model, the warm-up training was performed for 3 epochs firstly, and the momentum parameter of SGD was set as 0.8. After that, the learning rate was adjusted according to the following self-adaption: Init_lr =max (batch_size/64*Init_lr, 1e^{-4}), Min_lr = max (batch_size/64 *Min_lr, 1e^{-6}). Init_lr represented the initial learning rate, Min_lr represented the minimum learning rate, and batch-size was set as the current setup training process. The momentum parameter and weight decay were 0.937 and 0.0005, respectively.

The dataset has 480 images in our research, including 360 images in the training set, 40 images

in the validation set, and 40 images in the test set. We used Labelimg to label the assemblies in the images and obtained the corresponding images as well as the xml file of the label. The tools for data augmentation in the research were mosaic, mixup, copy_paste and paste_in. Finally, average precision (AP), mean average precision (mAP), and detection rate were used as evaluation metrics. The value of the detection accuracy of the target obtained by the training was 52.2, and the detection speed of each image was 10.8 ms. According to the length values of assembled MNMs by detection, the numbers of MNMs were then calculated (side length: 25 nm).

Real-time qPCR detection

The standard protocol using the following primers is as follows:
Cd80-F: ACCTGGGAAAAACCCCCAGAA; *Cd80*-R: GACAACGATGACGACGACTGT;
Cd86-F: AGAGCGGGATAGTAACGCTG; *Cd86*-R: CTTCACTCTGCATTTGGTTTTGCT;
Il1β-F: GACCTTCCAGGATGAGGACA; *Il1β*-R: AGCTCATATGGGTCCGACAG;
Il6-F: CCGGAGAGGAGACTTCACAG; *Il6*-R: TCCACGATTTCCCAGAGAAC;
Cd163-F: ACAAACCAAGAGTGCCGTGAG; *Cd163*-R: TCCAGTGCCTCCCAAAAATGACT;
Cd206-F: AGCCGAAGCTTAAGTCAGCA; *Cd206*-R: AGCCGAAGCTTAAGTCAGCA;
Il4-F: CCAAACGTCCTCACAGCAAC; *Il4*-R: TGCAGCTTATCGATGAATCCAGG;
Il10-F: TGAGGCGCTGTCATCGATTT; *Il10*-F: TGGCCTTGTAGACACCTTGG;
β-actin-F: ACTACATTCAATTCCATC; *β-actin*-R: CTAGAAGCACTTGCGGTG;
Pdl1-F: GCTCCAAAGGACTTGTACGTG; *Pdl1*-F: TGATCTGAAGGGCAGCATTTC;
Cd47-F: GCTTCTGGACTTGGCCTCAT; *Cd47*-R: CCTCTGGTTGGAAGCGACAA;
Egfr-F: GCCATCTGGGCCAAAGATACC; *Egfr*-R: GTCTTCGCATGAATAGGCCAAT;
Tgfb-F: CAGAGCTGCGCTTGCAGAG; *Tgfb*-R: GTCAGCAGCCGGTTACCAAG;
Gapdh-F: ATGAAGGGGTCGTTGATGGC; *Gapdh*-R: GGGTTCCTATAAATACGGACTGC.
